# Inhibition of ceramide synthesis ameliorates body wasting in a cancer cachexia model

**DOI:** 10.1172/JCI194687

**Published:** 2026-05-15

**Authors:** Pauline Morigny, Honglei Ji, Laura Cussonneau, Sabrina Zorzato, Yun Kwon, Fabien Riols, Doris Kaltenecker, Alisa Maier, Vignesh Karthikaisamy, Samantha Corrà, Tanja Krauss, Claudine Seeliger, Syed Qaaifah Gillani, Joël J. Tissink, Sandra Lacas-Gervais, Tuna Felix Samanci, Adriano Maida, Raul Terron-Exposito, Angela Trinca, Christine von Toerne, Leonardo Nogara, Melina Claussnitzer, Olga Prokopchuk, Jeannine Bachmann, Mauricio Berriel Diaz, Laure B. Bindels, Ondrej Kuda, Hans Hauner, Mark Haid, Stephan Herzig, Carlo Fiore Viscomi, Jerome Gilleron, Anja Zeigerer, Bert Blaauw, Maria Rohm

**Affiliations:** 1 Institute for Diabetes and Cancer (IDC), Helmholtz Munich, Neuherberg, Germany.; 2 Joint Heidelberg-IDC Translational Diabetes Program, University Hospital, Heidelberg, Germany.; 3 German Center for Diabetes Research (DZD), Munich, Germany.; 4 Veneto Institute of Molecular Medicine, Padua, Italy.; 5 Department of Biomedical Sciences, University of Padua, Padua, Italy.; 6 European Center for Angioscience, Medical Faculty Mannheim, Heidelberg University, Mannheim, Germany.; 7 Metabolomics and Proteomics Core Facility, Helmholtz Munich, Neuherberg, Germany.; 8 Else Kröner Fresenius Center for Nutritional Medicine, School of Life Sciences, and; 9 ZIEL Institute for Food and Health, Technical University of Munich, Freising-Weihenstephan, Germany.; 10 Université Côte d’Azur, Centre Commun de Microscopie Appliqué, Nice, France.; 11 Department of Pharmaceutical Sciences, University of Padua, Padua, Italy.; 12 Novo Nordisk Foundation Center for Genomic Mechanisms of Disease, Broad Institute of MIT and Harvard, Cambridge, Massachusetts, USA.; 13 Diabetes Unit and Center for Genomic Medicine, Massachusetts General Hospital, Boston, Massachusetts, USA.; 14 Department of Medicine, Harvard Medical School, Boston, Massachusetts, USA.; 15 Department of Surgery, Klinikum rechts der Isar, School of Medicine, Technical University of Munich, Munich, Germany.; 16 Metabolism and Nutrition Research Group, Louvain Drug Research Institute, Université Catholique de Louvain, Brussels, Belgium.; 17 Welbio Department, WEL Research Institute, Wavre, Belgium.; 18 Laboratory of Metabolism of Bioactive Lipids, Institute of Physiology of the Czech Academy of Sciences, Prague, Czechia.; 19 Institute of Nutritional Medicine, School of Medicine and Health, and; 20 Chair Molecular Metabolic Control, Technical University Munich, Munich, Germany.; 21 Department of Biosciences, University of Milano, Milano, Italy; 22 Université Côte d’Azur, INSERM, Adipo-Cible Research Study Group, Mediterranean Center of Molecular Medicine (C3M), Team “Insulin Resistance in Obesity and Type 2 Diabetes”, Nice, France.; 23 German Center for Cardiovascular Research (DZHK), partner site Munich Heart Alliance, Munich, Germany.

**Keywords:** Metabolism, Oncology, Cancer, Lipidomics, Mitochondria

## Abstract

Cachexia is a metabolic wasting syndrome affecting many patients with cancer, with poor survival outcomes. Disturbed lipid metabolism is a hallmark of cachexia, and our previous work has identified increased levels of circulating ceramides, which are bioactive lipids with adverse effects in metabolic diseases, as biomarkers for cachexia in mouse models and patients. Here, we investigated the role of ceramides on cachexia development using the well-established C26 colon carcinoma model. We demonstrated that elevated ceramides in cachexia arose from increased liver synthesis. We showed that ceramides directly contributed to impaired mitochondrial function and energy homeostasis in cachexia target tissues. Targeting ceramide synthesis using miRNA interference, or myriocin, an approved compound targeting the key synthesis enzyme serine palmitoyltransferase (SPT), improved markers of muscle atrophy in cachectic male mice. Importantly, we demonstrated that key enzymes involved in ceramide production were also elevated in livers, but not in other organs, of patients with cancer cachexia, correlating with disease severity. Our data place ceramides as contributors to metabolic dysfunction in cachexia and highlight the suitability of the ceramide synthesis pathway for therapeutic targeting.

## Abstract 2

High circulating ceramides define cancer cachexia in mice and patients. Targeting ceramide synthesis counteracts mitochondrial dysfunction, impaired energy homeostasis and muscle atrophy in cachectic mice.

## Graphical abstract

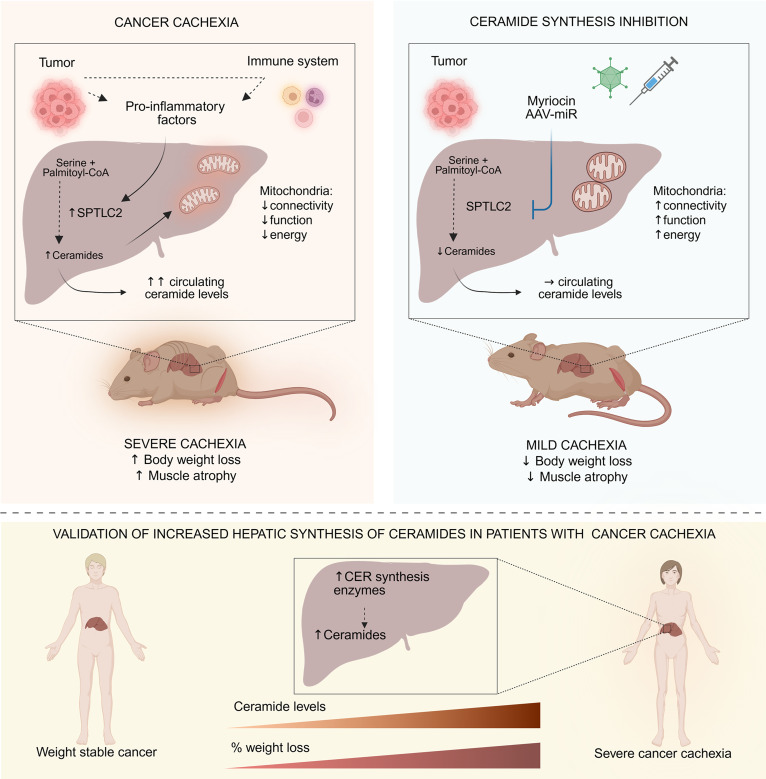

## Introduction

Cancer cachexia is a wasting syndrome affecting many patients with cancer and is characterized by an involuntary loss of body mass that cannot be reversed by nutritional supplementation (1, 2). Cachexia therefore limits the therapeutic window for patients with cancer and drastically reduces both their quality of life and overall survival. Mechanistically, it is driven by systemic inflammation, suppression of anabolic pathways, and activation of catabolic processes, leading to the loss of lean and fat body mass (1). Cardiac and skeletal muscle proteolysis, white adipose tissue lipolysis and energy expenditure, the hepatic acute-phase response (APR) and mitochondrial dysfunction, as well as anorexia are hallmarks of cancer cachexia. Currently, no standardized treatments exist for this syndrome, and although several proteins have been identified as procachectic mediators (e.g., IL-6, IL-1β, growth differentiation factor 15, leukemia-inhibitory factor), no approved therapies targeting these factors exist to date (1, 2). We therefore focused on the role of bioactive lipids in cancer cachexia, which are important signaling molecules overlooked in this context. Performing broad-range plasma lipidomics, we previously reported high circulating levels of sphingolipids, especially ceramides (CERs) and modified CERs, as a hallmark of cancer cachexia in both cachectic animals bearing tumors of different entities and cachectic patients with cancer (3).

As essential components of cellular membranes, CERs control inter-tissue communication, ER homeostasis, and mitochondria dynamics and capacity to produce energy. CERs can also be secreted and transferred between tissues, leading to systemic effects (4). Pathophysiological signaling pathways controlling CER synthesis are still not well characterized, but CER levels increase upon excessive fat intake, oxidative stress, or a proinflammatory environment (5–7). Accordingly, CERs have been implicated in many pathophysiological processes including age-induced sarcopenia (8–10), tumorigenesis of different cancer entities (11, 12), obesity-associated insulin resistance and liver diseases (4, 13–18), cardiovascular diseases (19, 20), and neurological disorders (21), among others. CERs directly interfere with insulin signaling in metabolic tissues (e.g., liver, adipose tissue, skeletal muscle), promote ER stress, mitochondrial dysfunction, and apoptosis, and interfere with feeding behavior by acting on the CNS (6, 9, 14, 22–24). CERs therefore represent promising targets, not only as biomarkers of disease progression (25), but also because of their therapeutic potential for such pathologies (12, 23, 26, 27).

CERs are mostly synthesized de novo in the ER by the sequential action of serine palmitoyltransferases (SPTs), 3-ketodihydrosphingosine reductase (KDSR), ceramide synthases (CERSs), and dihydroceramide desaturases (DEGs) (5, 7). CERS isoforms 1-6 are responsible for the synthesis of different CER species. CERS5-6 mostly synthesize CER(16:0), CERS1 synthesizes CER(18:0), while CERS2 synthesizes long-chain CER [e.g., CER(22:0)]. Different CER species have been reported to exert divergent biological effects, and while CERS6- or CERS1-derived CERs are toxic lipid species (14, 16, 22, 28, 29), CERS2-derived CERs are often considered beneficial (15, 30). CERs can also be retrieved from sphingomyelins (SMs) via the action of acid sphingomyelinase (SMPD1) in lysosomes (i.e., salvage pathway) (5, 7). CERs can be further modified in the Golgi apparatus through the incorporation of sugar moieties, leading to the formation of hexosyl-CERs (HCERs) and lactosyl-CERs (LCERs), precursors of complex glycosphingolipids (i.e., gangliosides), by action of the UDP-glucose ceramide glucosyltransferase (UGCG) and β-galactoside α-2,3-sialyltransferase 5 (ST3GAL5). While all cell types can produce CERs, liver and adipose tissues are considered the main tissues contributing to the circulating levels (4).

Given their varied biological effects and our previous findings in the cachexia context (3), CERs represent promising targets to counteract cachexia. In the present study, we show that elevated CERs in cachexia arose from increased liver synthesis. As proof of concept of the therapeutic potential of targeting CERs for cancer cachexia, we chose a pharmacological approach, treating animals with a specific SPT inhibitor. We show that CER synthesis inhibition ameliorated body wasting in cancer, especially the loss of lean mass, and have uncovered underlying mechanisms. We further validated our key results by performing gene silencing of SPT in liver. Ultimately, we provide evidence for the relevance of our findings in patients with cancer cachexia.

## Results

### The liver is responsible for the increased circulating levels of CERs in cachexia.

**Figure 1:**
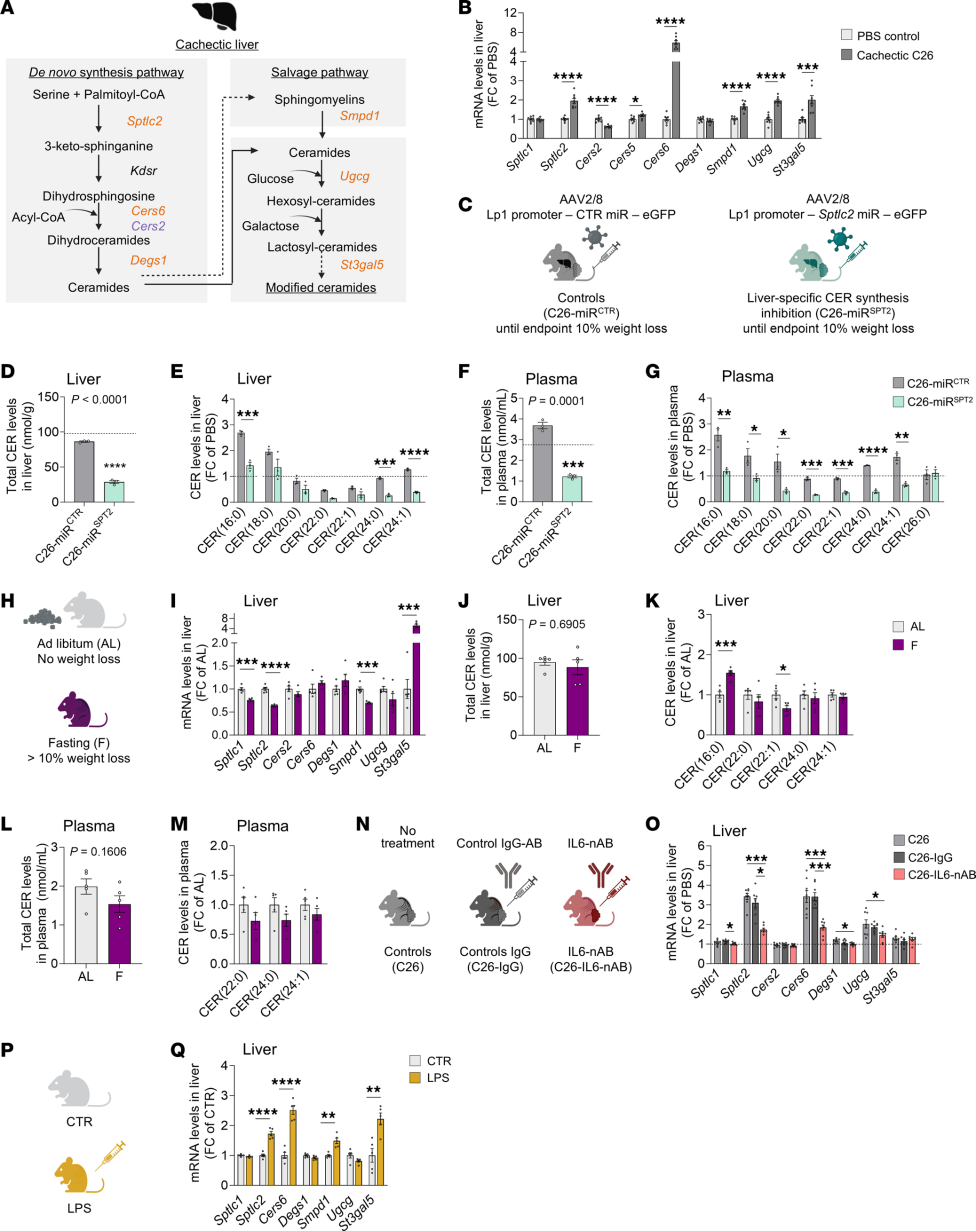
The liver is responsible for increased circulating levels of CERs in cachexia. (**A**) Regulation of CER synthesis enzymes in the livers of cachectic animals (3). Orange: Upregulated in cancer cachexia; purple: downregulated in cancer cachexia. Image created in BioRender. (**B**) Hepatic expression of CER synthesis enzymes in another cohort of healthy controls (PBS-injected) and cachectic C26 tumor–bearing mice (*n* = 8 animals per group). (**C**) Mice injected with hepatocyte-specific adeno-associated viruses (AAVs) expressing either a control miRNA (miR^CTR^) or a miRNA against *Sptlc2* (miR^SPT2^), and later with cachexia-inducing C26 carcinoma cells. The experiment ended once each animal had reached 10% weight loss for comparable cachexia severities (*n* = 3 animals per group). **D**–**G**) Total CER levels (**D** and **F**) and CER composition relative to PBS controls (**E** and **G**) in liver (**D** and **E**) and plasma (**F** and **G**). Horizontal dotted lines show reference levels of PBS controls (*n* = 3 animals per group). (**H**) Mice were fasted for 24 hours to induce a weight loss of greater than 10%, comparable to cachexia (fasting [F]) or were maintained on an ad libitum [AL] chow diet (*n* = 5 animals per group). (**I**–**M**) Hepatic mRNA expression of CER synthesis enzymes (**I**). (**J**–**M**) Total CER levels (**J** and **L**) and CER composition relative to AL controls (**K** and **M**) in liver (**J** and **K**) and plasma (**L** and **M**). (**N**) Mice injected with C26 cells were either untreated (C26), treated with a control IgG antibody (C26-IgG), or treated with an IL-6–neutralizing antibody (C26-IL6-nAB). (**O**) Hepatic mRNA expression of CER synthesis enzymes (*n* = 8 animals per group). (**P**) Mice were injected with LPS or served as untreated controls (CTR) (*n* = 5 animals per group). (**Q**) Hepatic mRNA expression of CER synthesis enzymes. Data indicate the mean ± SEM. *P < 0.05, **P < 0.01, ***P < 0.001, and ****P < 0.0001, by unpaired, 2-tailed *t* test (**B**, **D**–**G**, **I**, **K**–**M**, and **Q**), Mann-Whitney *U* test (**B**, **E**, and **I**–**K**), unpaired, 1-way ANOVA with Tukey’s post hoc test (**O**), or Kruskal-Wallis with Dunn’s post hoc test (**O**). (**A**, **C**, **H**, **N**, **P**) Image created in BioRender.

We have previously reported increased expression of CER synthesis enzymes in the livers of cachectic animals (Figure 1, A and B). To prove that liver is the main tissue to target for decreasing circulating CER levels in cachexia, we blocked the expression of SPTLC2 (i.e., key enzyme of the de novo synthesis pathway, Figure 1A) via hepatocyte-specific miRNA interference (Figure 1C) and induced cachexia in male mice via subcutaneous injection of C26 colon carcinoma cells. Mice were necropsied once they all reached a similar tumor size and weight loss of greater than 10% to allow a proper comparison of CER levels (Supplemental Figure 1, A and B; supplemental material available online with this article; https://doi.org/10.1172/JCI194687DS1). miRNAs did not lead to any liver toxicity (Supplemental Figure 1, C–F), and we confirmed the appropriate expression of the transgene via comparable levels of the EGFP reporter in livers of cachectic animals expressing a control miRNA or miRNA against *Sptlc2* (Supplemental Figure 1G). *Sptlc2* knockdown was enzyme and tissue specific (Supplemental Figure 1, G and H) and led to protein levels comparable to those in tumor-free healthy control animals injected with PBS (Supplemental Figure 1, I and J). Liver-specific inhibition of CER synthesis almost completely normalized both liver and plasma CER and HCER levels (Figure 1, D–G, Supplemental Figure 1, K–N), confirming the central role of this tissue for later targeted therapies. Interestingly, we did not observe an increase in total CER levels in liver upon cachexia but rather a shift in their profile, with an accumulation of toxic CER species [CER(16:0), CER(18:0), CER(24:1)] (Figure 1, D and E) and a decrease in the CERS2-derived CER(22:0), in accordance with reduced *Cers2* gene expression (Figure 1, A and B). Total HCER levels in liver were strongly increased, confirming the ongoing CER synthesis in cachectic livers (Supplemental Figure 1, K and L).

We checked whether increased lipolysis and fatty acid overload from adipose tissue to the liver could be the main upstream regulators of CER synthesis in cachexia, assessing CER profiles in 24-hour fasted animals (Figure 1H). These mice exhibited a body composition similar to that of cachectic animals (significant decrease in body weight, adipose tissue, skeletal muscle and liver mass) (Supplemental Figure 1, O–S). Increased lipolysis was confirmed by high circulating levels of glycerol and nonesterified fatty acids (Supplemental Figure 1, T and U). Nevertheless, neither the expression of CER synthesis enzymes (Figure 1I), nor the levels of CER in the liver and plasma were affected by the increased flux of fatty acids to the liver (Figure 1, J–M, Supplemental Figure 1, V–Y), which instead led to an accumulation of intrahepatic triacylglycerols (Supplemental Figure 1Z). Lipolysis slightly increased the levels of a few CER species but to a much smaller extent than in cachexia (Figure 1, K and E, and Supplemental Figure 1, Y and N). Proinflammatory cytokines like IL-6 are suspected upstream regulators of CER synthesis and important tumor-derived factors contributing to cachexia. We therefore assessed the expression of CER synthesis enzymes in the livers of cachectic C26 tumor–bearing mice treated or not with an IL-6–neutralizing antibody (Figure 1N) (31, 32). IL-6 inhibition almost completely normalized CER synthesis enzyme expression to healthy control levels (Figure 1O). Ultimately, we confirmed the strong induction of CER synthesis enzymes in animals with LPS-induced acute inflammation (Figure 1, P and Q).

Altogether, those data indicate that liver is the main tissue responsible for CER synthesis in cachexia and that CER synthesis is not the consequence of enhanced lipid release from adipose tissue but rather is associated with tumor- or host-derived proinflammatory factors.

### Pharmacological inhibition of CER synthesis ameliorates body wasting in advanced cachexia.

**Figure 2:**
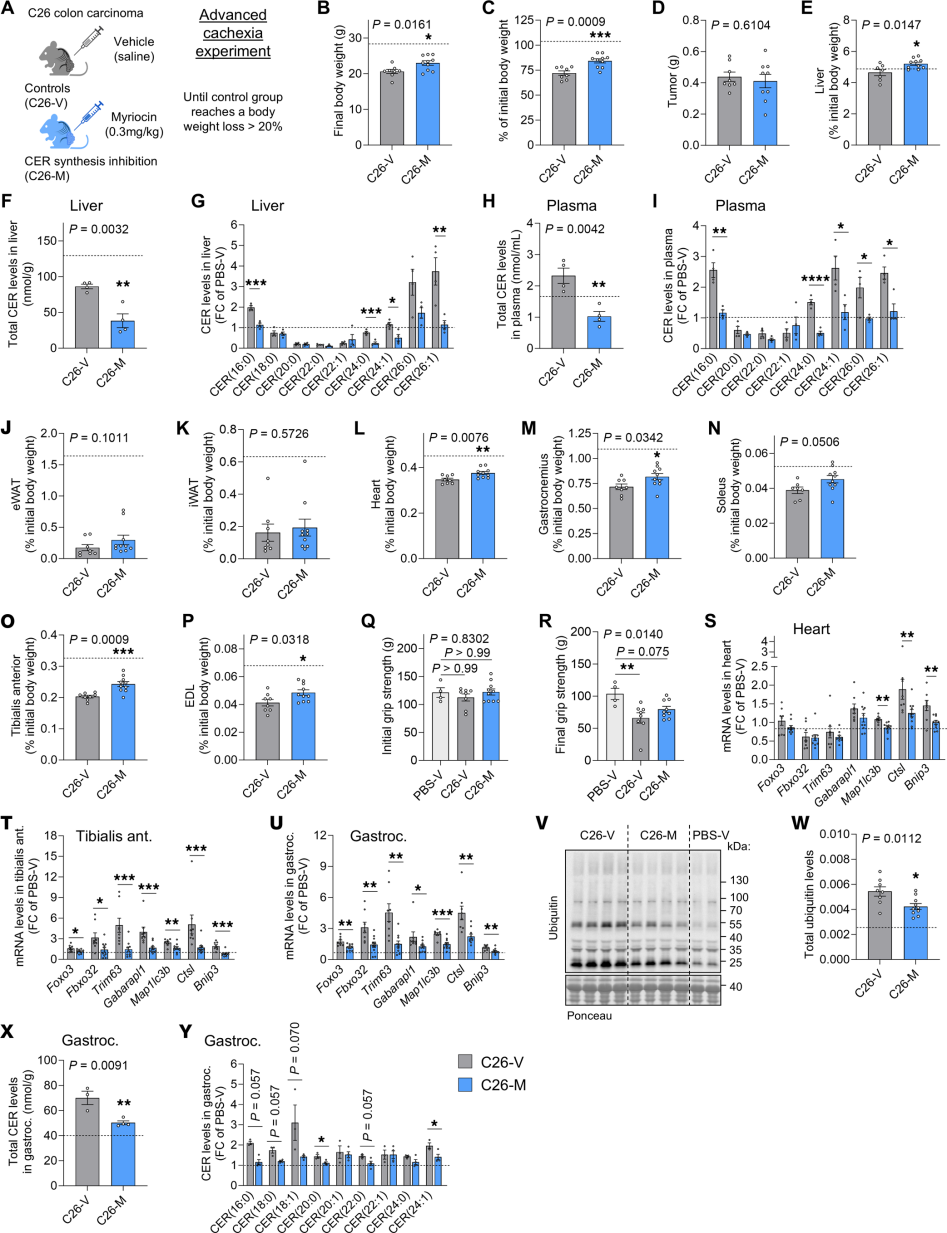
Pharmacological inhibition of CER synthesis ameliorates body wasting in advanced cachexia. (**A**) Mice were injected with C26 cells and treated with vehicle (C26-V, *n* = 8 animals, unless stated otherwise) or myriocin (C26-M, *n* = 10 animals, unless stated otherwise). The experiment was ended once control animals lost more than 20% of their body weight (advanced cachexia experiment). Horizontal dotted lines show reference levels for healthy controls (PBS-V, PBS-injected mice treated with vehicle, *n* = 4 animals). Image created in BioRender. (**B**) Final body weights. (**C**) Body weight loss. (**D**) Final tumor weights (C26-M, *n* = 9). (**E**) Liver weights (C26-V, *n* = 7). (**F**–**I**) Total CER levels (**F** and **H**) and CER composition (fold change of PBS-V, **G** and **I**) in liver (**F** and **G**) and plasma (**H** and **I**) (*n* = 4 animals per group). (**J** and **K**) Epididymal white adipose tissue (eWAT) (2 depots) weights (**J**) and inguinal WAT (iWAT) (1 depot) weights (**K**). (**L**) Heart weight. (**M**–**P**) Skeletal muscle weights: gastrocnemius (**M**), soleus (**N**) (C26-V, *n* = 7), tibialis anterior (**O**), and extensor digitorum longus (EDL) (**P**). (**Q** and **R**) Initial (**Q**) and final (**R**) (C26-M, *n* = 9) grip strength. (**S**–**U**) Atrogen and autophagy markers mRNA expression in heart (**S**) and skeletal muscles (**T** and **U**). (**V**) Total ubiquitin levels in gastrocnemius. Ponceau was used as a loading control. (**X** and **Y**) Total CER levels (**X**) and CER composition (**Y**) in gastrocnemius (C26-V, *n* = 3 animals; C26-M, *n* = 4 animals). Data indicate the mean ± SEM. *P < 0.05, **P < 0.01, ***P < 0.001, and ****P < 0.0001, by unpaired, 2-tailed *t* test (**B**–**I**, **L**–**P**, **S**–**U**, and **W**–**Y**), Mann-Whitney *U* test (**I**–**K**, **S**–**U**, and **Y**), unpaired, 1-way ANOVA with Dunnett’s test (**R**), or Kruskal-Wallis with Dunn’s post hoc test (**Q**).

We next applied a pharmacological approach to counteract CER synthesis in the C26 cachexia model to assess the pathway’s therapeutic relevance. Animals were treated with myriocin, a specific SPT inhibitor, once tumors were visible, until most vehicle-treated C26 animals reached an advanced cachectic phenotype characterized by body weight loss of more than 20% (Figure 2A). The dose and route of administration of myriocin were defined on the basis of the literature (13, 33). Treatment did not induce a toxic phenotype in tumor-free, PBS-injected control animals (Supplemental Figure 2) or in C26 tumor–bearing mice (Figure 2, B–E, and Supplemental Figure 3, A–E). Importantly, myriocin efficiently reduced body weight loss without affecting tumor growth (Figure 2, B–D, horizontal dotted lines show reference levels of tumor-free healthy controls). We also observed trends toward an increase in circulating glucose, fatty acids, and triacylglycerols, suggesting an overall improvement in the nutritional status and/or control of energy homeostasis in these animals (Supplemental Figure 3, F–H). We confirmed the strong reduction in all sphingolipids in both liver and plasma of myriocin-treated animals, including CER, HCER, LCER, and SM levels, which were largely comparable to those in healthy controls (Figure 2, F–I, and Supplemental Figure 3, I–P). While myriocin did not affect fat mass (Figure 2, J and K), we observed significant improvements in the weights of cardiac and many skeletal muscles (Figure 2, L–P, and Supplemental Table 1), one of the most important determinants of morbidity and mortality in patients with cancer (2). Accordingly, grip strength of myriocin-treated animals at the endpoint was less significantly impaired than that of vehicle-treated animals compared with PBS-treated controls, suggesting a moderate functional improvement (Figure 2, Q and R). The potential of CER inhibition to counteract muscle proteolysis was further confirmed by the reduction in atrophy and autophagy marker gene expression in heart and skeletal muscles (Figure 2, S–U), together with a significant decrease in ubiquitinylated protein levels in gastrocnemius muscle (Figure 2, V and W). Interestingly, CER levels in gastrocnemius muscles of cachectic animals also mirrored the hepatic and circulating levels (Figure 2, X and Y, and Supplemental Figure 3, Q–T).

Our data therefore highlight the potential of targeting CER synthesis pathways for the treatment of cachexia.

### Pharmacological inhibition of CER synthesis improves mitochondrial function in livers of cachectic animals.

We next looked for the underlying mechanisms responsible for the beneficial effects of myriocin on cachexia development. To determine the primary chain of events linking CERs to wasting processes, we performed another myriocin experiment, in which C26 tumor–bearing animals were necropsied once vehicle-treated control animals lost more than 8% of their body weight (Supplemental Figure 4A). We found that at such an early stage, CER synthesis inhibition only led to moderate improvements in body weight loss without affecting tumor growth (Supplemental Figure 4, B–F). The use of metabolic cages revealed no effect of myriocin on food intake but a significant increase in water intake during the time course of cachexia development (Supplemental Figure 4, G and H). As cachectic animals tend to crumble their food, water intake may be a more reliable indicator of improved nutritional state. At the endpoint, cachectic C26 tumor–bearing mice showed decreased physical activity and a reduced respiratory exchange ratio (indicating increased lipid oxidation) (Supplemental Figure 4, I and J), as well as increased metabolism and energy expenditure (Supplemental Figure 4, K–M) compared with PBS-treated controls. However, myriocin did not affect those parameters at such an early stage of cachexia. Once again, treatment did not induce any toxicity (Supplemental Figure 4, N–Q) but was very efficient at reducing CER levels (Supplemental Figure 4, R–V). Once again, myriocin did not reduce fat loss (Supplemental Figure 4W) but already caused moderate improvements in cardiac and skeletal muscle mass (Supplemental Figure 4, X and Y, and Supplemental Table 2). Furthermore, cachexia induced significant functional impairment in vehicle- but not myriocin-treated animals (Supplemental Figure 4, Z and AA). The effect on muscle loss could not be explained by a reduction in proteolysis marker expression at such an early stage (Supplemental Figure 4, AB and AC), suggesting that another tissue or tissues may be driving this systemic effect.

**Figure 3:**
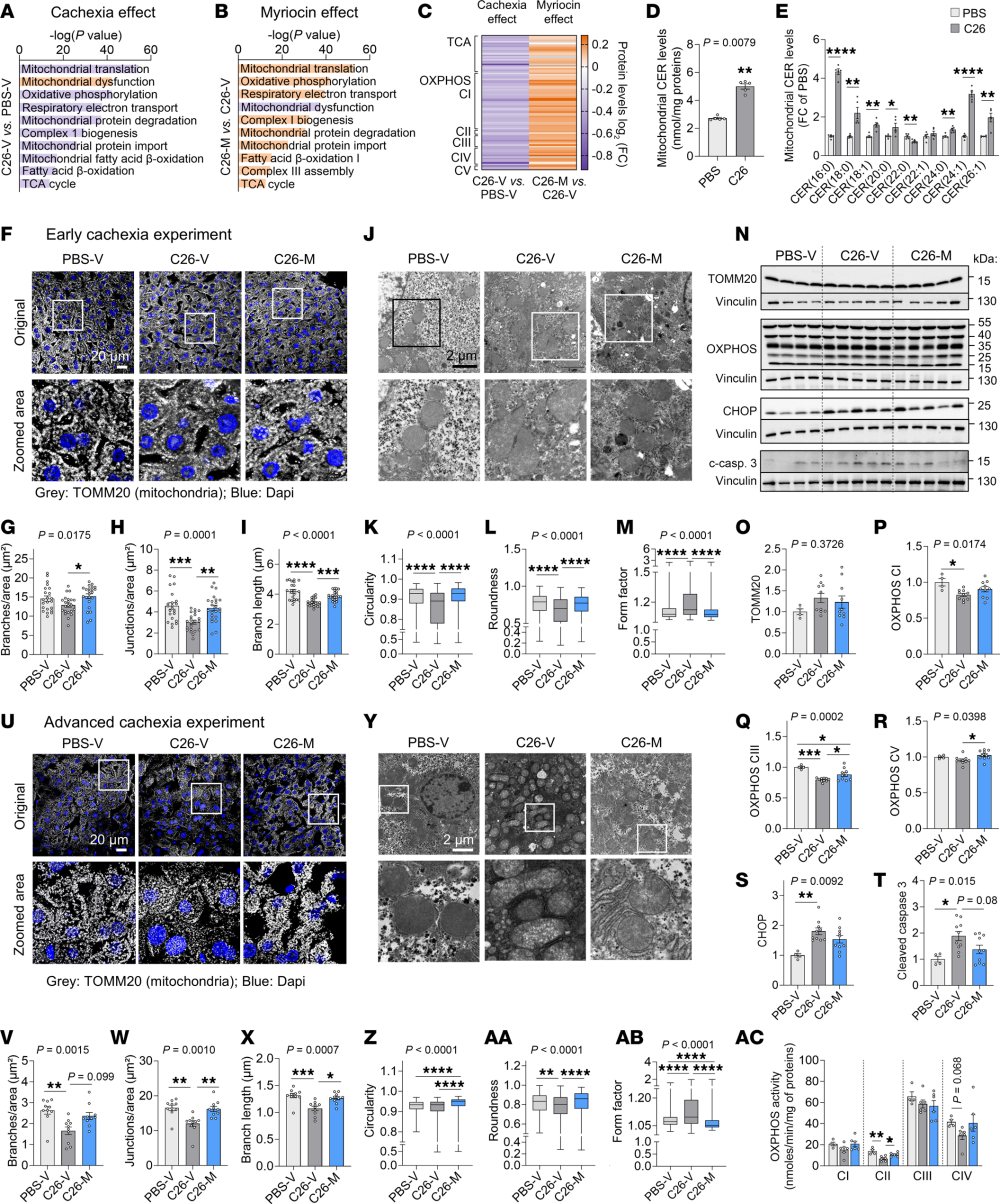
Pharmacological inhibition of CER synthesis improves mitochondrial function in the livers of cachectic animals. (**A**–**C** and **F**–**T**) C26 tumor–bearing mice were treated with vehicle (C26-V) or myriocin (C26-M). The experiment ended once animals reached weight loss of greater than 8% (early cachexia experiment). (**A**–**C**) Liver proteomics (PBS-injected, vehicle-treated mice [PBS-V]: *n* = 4; C26-V, C26-M: *n* = 5 animals). Top mitochondria-related pathways affected by cachexia (**A**) and myriocin (**B**). Purple: Downregulated pathways; orange: upregulated pathways. (**C**) Expression of mitochondria-related proteins. (**D** and **E**) Total CER levels (**D**) and CER composition (**E**) in crude mitochondria from liver of PBS-injected animals and C26 tumor–bearing animals (*n* = 5 animals per group). (**F**–**I**) Confocal microscopy analysis of liver mitochondria (*n* = 3 animals per group; each dot represents 1 image containing >30 hepatocytes quantified; PBS-V, C26-V: *n* = 22 images; C26-M: *n* = 23 images). (**F**) Original magnification, ×60; insets, ×3.5. Quantification of mitochondrial branches (**G**), junctions (**H**), and mean branch length (**I**). (**J**–**M**) Electron microscopy images and analysis of liver mitochondria (*n* = 3 animals per group; *n* = 509–646 mitochondria). (**J**) Original magnification, ×12,000; insets, ×2. Quantification of mitochondrial circularity (**K**), roundness (**L**), and form factor (**M**). (**N**–**T**) Western blots of liver mitochondria-related proteins: TOMM20 (**O**), OXPHOS complexes (**P**–**R**, from top to bottom: CV, CIII, CIV, CII, CI), CHOP (**S**) and cleaved caspase-3. Vinculin as loading control (PBS-V, *n* = 4, C26-V, -M: *n* = 10 animals). Quantification relative to PBS-V. (**U**–**AC**) Advanced cachexia experiment. Confocal microscopy (**U**–**X**, *n* = 3 animals per group; *n* = 10 images; Original magnification, ×60; insets, ×3.5) and electron microscopy (**Y**–**AB**, *n* = 3 animals per group; *n* = 266–385 mitochondria) analyses of liver mitochondria as in **F**–**M**. (**AC**) OXPHOS activity in liver (PBS-V: *n* = 4; C26-V, C26-M: *n* = 6 animals). Scale bars: 20 μm (**F** and **U**) and 2 μm (**J** and **Y**). Original magnification, ×12000; insets, ×3.5. Data indicate the mean ± SEM. (**D**, **E**, **G**–**I**, **O**–**X**, and **AC**) and the median minimum to maximum values (**K**–**M** and **Z**–**AB**). *P < 0.05, **P < 0.01, ***P < 0.001, and ****P < 0.0001, by unpaired, 2-tailed *t* test (**D** and **E**), Mann-Whitney *U* test (**E**), unpaired, 1-way ANOVA with Tukey’s test (**H**, **O**–**R**, **T**, and **AC**), or Kruskal-Wallis with Dunn’s test (**G**, **I**, **K**–**M**, **S**, **V**–**X**, **Z**, and **AA**–**AC**). FC, fold change; CI, Complex I; CII, Complex II; CIII, Complex III; CIV, Complex IV; CV, Complex V.

As liver is the primary tissue for CER synthesis in cachexia (Figure 1), we explored how this tissue may affect systemic energy homeostasis. Liver is a central organ in the control of systemic insulin sensitivity, and insulin resistance has been reported in animal models and patients with cancer cachexia (34–36), potentially counteracting the anabolic effect of insulin. We measured protein levels of different actors of the insulin signaling cascade, however, neither cachexia nor myriocin interfered with insulin signaling (Supplemental Figure 5, A–F). We also observed no signs of insulin resistance in cachectic C26 tumor–bearing mice at any time in the course of cachexia development, as assessed both by insulin tolerance tests (Supplemental Figure 5, G–O) and the activation of the insulin signaling cascade in liver and muscle after insulin stimulation (Supplemental Figure 5, P–S). C26 tumor–bearing mice were even more responsive to a low dose of insulin, which was too low to induce a response in healthy PBS-treated controls. Beyond insulin sensitivity, we also assessed the effect of CERs on liver acute phase response and systemic inflammation. Neither the expression of genes encoding APR proteins nor the plasma levels of these proteins were altered upon myriocin treatment in cachexia (Supplemental Figure 5, T–W). Plasma IL-6 levels were reduced, but only at an advanced cachexia stage (Supplemental Figure 5, X and Y). Therefore, insulin resistance, liver APR or CER-induced systemic inflammation are unlikely to play a central role in the deleterious effect of CERs on cachexia development. We next performed liver proteomics analysis to understand at what level CERs may interfere with liver function (Supplemental Figure 6, A and B). Pathway analysis of significantly altered proteins highlighted mitochondrial dysfunction as one of the top pathways affected by cachexia and rescued by myriocin (Supplemental Figure 6, C and D). Cachexia was notably characterized by a decrease in many mitochondrial pathways related to energy production, which were reversed upon myriocin treatment (Figure 3, A and B). In accordance, a high number of mitochondrial proteins involved in the TCA cycle or oxidative phosphorylation (OXPHOS) complexes were reduced in cachexia and increased by myriocin (Figure 3C).

To investigate the link between liver CERs and mitochondrial function, we aimed to confirm the increase in mitochondrial CER content upon cachexia. We performed lipidomics analysis of the liver crude mitochondrial fraction in tumor-free controls and C26 tumor–bearing mice. While total CER levels in whole liver tended to be decreased in cachexia due to the strong depletion of CER(22:0) (Figure 2, F and G), total CER content in crude mitochondria increased 2-fold and CER(16:0) levels 4-fold (Figure 3, D and E, and Supplemental Figure 6, E–H). As CERS6-derived CERs [mostly CER(16:0)] have been previously shown to promote mitochondrial fission and dysfunction (14), we next assessed mitochondrial ultrastructural morphology and connectivity in livers of cachectic animals upon myriocin treatment at the early disease stage (Supplemental Figure 4). We observed reduced branch and junction numbers in cachectic vehicle-treated animals, reduced branch length (Figure 3, F–I), decreased mitochondrial circularity and roundness, and increased form factor (Figure 3, J–M). All those effects of cachexia were reversed upon myriocin treatment, confirming the adverse effect of CERs on mitochondrial structure in cachexia. We next assessed protein expression of markers of mitochondrial mass (TOMM20), respiratory function (OXPHOS complexes), and stress (C/EBP homologous protein [CHOP], cleaved caspase 3) (Figure 3, N–T, and Supplemental Figure 6, I and J). While mitochondria numbers did not seem to be affected in early cachexia, we observed decreased levels of some OXPHOS complexes, which was prevented by myriocin, confirming our proteomics data. Mitochondrial stress markers were increased in livers of cachectic animals and partially reversed by myriocin, suggesting an improvement in mitochondrial function. We confirmed the strong effect of CERs on liver mitochondria by performing similar analyses in livers of animals with advanced cachexia (Figure 2). Alteration in mitochondrial morphology and connectivity in cachexia (Figure 3, U–AB, and Supplemental Figure 6, K–S) was associated with reduced activity of several OXPHOS complexes (Figure 3AC and Supplemental Figure 6T), which was rescued upon myriocin treatment.

Overall, our data suggest that CERs affected mitochondrial morphology and connectivity in cachectic livers as a primary event, thereby reducing mitochondrial function and the ability to produce energy early in the development of cachexia.

### Liver-specific knockdown of CER synthesis improves cachectic phenotypes and mitochondrial function in livers of cachectic animals.

**Figure 4:**
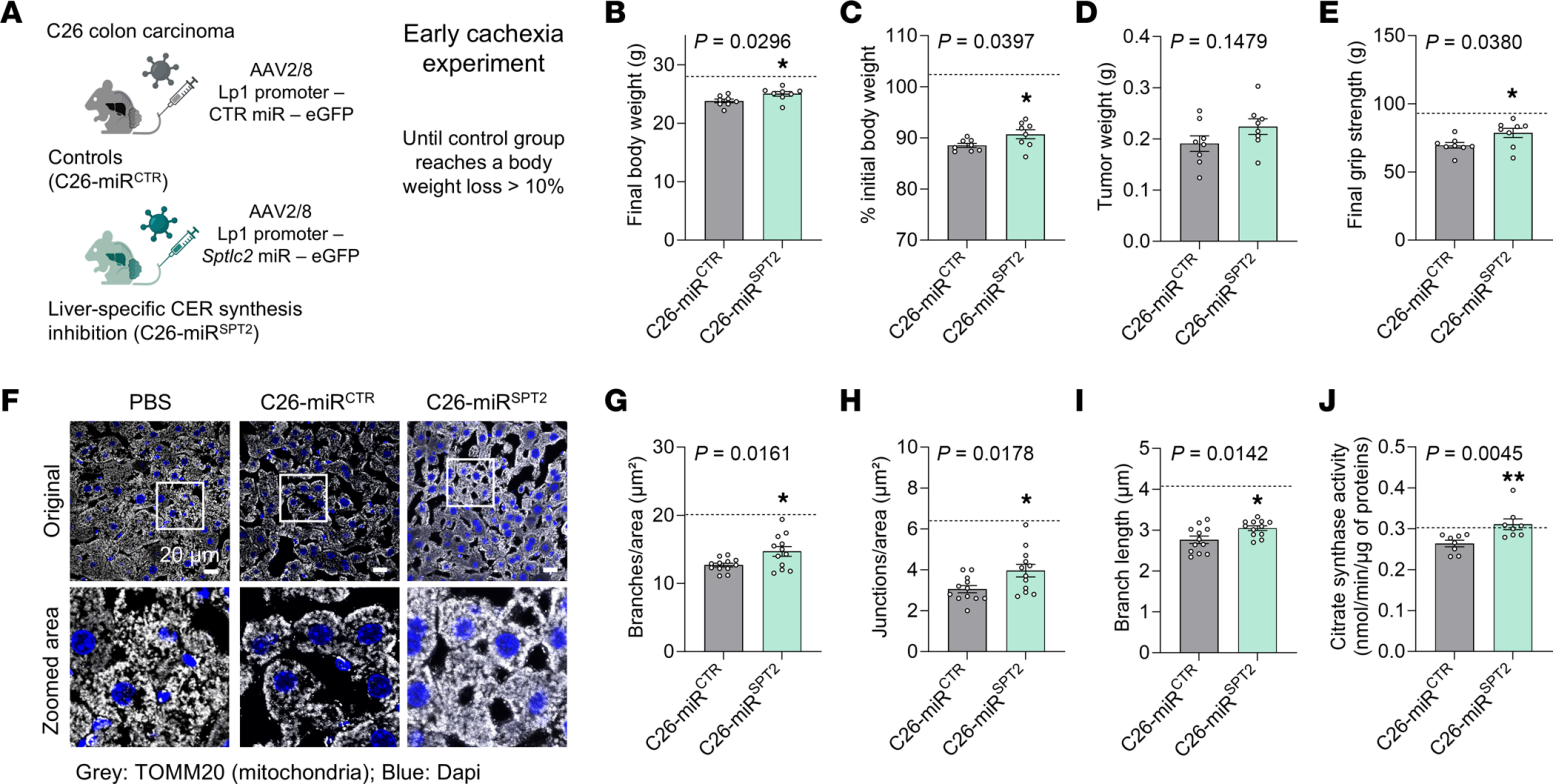
Liver-specific knockdown of CER synthesis improves cachectic phenotypes and mitochondrial function in livers of cachectic animals. (**A**) Mice were injected with an AAV expressing a control miRNA (miR^CTR^) or a miRNA against *Sptlc2* (miR^SPT2^) in a hepatocyte-specific manner. Mice were then injected with cachexia-inducing C26 cells (*n* = 8 animals per group, unless stated otherwise). The experiment ended once control animals lost more than 10% of their body weight (early cachexia experiment). Image created in BioRender. (**B**) Final body weights, (**C**) body weight loss, (**D**) final tumor weights, and (**E**) final grip strength. Horizontal dotted lines show reference levels of healthy controls (PBS-injected mice: *n* = 7). (**F**–**I**) Confocal microscopy images and analysis of liver mitochondria from 4 animals per group (*n* = 12 images quantified per group). Scale bars: 20 μm. Original magnification, ×60; inset, ×3.5. Quantification of mitochondrial morphology: branches (**G**), junction numbers (**H**), and mean branch length (**I**), indicating mitochondrial connectivity. (**J**) Citrate synthase activity in liver. Data indicate the mean ± SEM. *P < 0.05 and **P < 0.01, by unpaired, 2-tailed *t* test (**B**–**E** and **G**–**I**) or Mann-Whitney *U* test (**J**).

We next wanted to confirm whether the observed effects on mitochondrial function were specific to CERs and not off-target effects of myriocin. To do so, we performed an experiment using the AAV-miR^SPT2^ previously validated (Figure 1, C–G, and Supplemental Figure 1, A–N) and ended it as soon as the control tumor-bearing animals reached a weight loss of more than 10% (Figure 4A). With this setup, we observed a moderate but significant improvement in body weight loss upon liver-specific SPT knockdown (Figure 4, B and C, and Supplemental Figure 7, A–C), without any effect on final tumor weight (Figure 4D). Once again, we confirmed the strong reduction in circulating CER levels (Supplemental Figure 7, D–F). Although there was no significant difference in fat or muscle tissue weights at the endpoint (Supplemental Figure 7, G–I, and Supplemental Table 3), we still observed an improvement in muscle grip strength and a decrease in muscle atrophy markers (Figure 4E and Supplemental Figure 7, J and K), suggesting an overall improvement of muscle wasting. Interestingly, total CER levels and levels of CER(18:0) and CER(18:1) in muscle, the main CER species produced in this tissue, were not reduced upon liver-specific inhibition of CER synthesis, arguing for a local CER biosynthesis in cachectic muscle (Supplemental Figure 7, L and M). Nevertheless, the levels of CER(16:0), the main species affected in liver and plasma in cachexia, were fully reversed in muscles of C26-miR^SPT2^ mice, supporting the hypothesis of an inter-tissue crosstalk. Focusing on the liver, we next confirmed that the beneficial effects of CER inhibition on cachexia were not associated with alterations in the APR (Supplemental Figure 7N). Looking at mitochondrial phenotypes, SPT knockdown was associated with a partial improvement in readouts of mitochondrial connectivity (Figure 4, F–I) and activity of citrate synthase in livers of tumor-bearing animals (Figure 4J).

Genetic inhibition of CER synthesis therefore recapitulated the beneficial effects observed with myriocin, improving mitochondrial function in the liver and body wasting.

### CERs directly interfere with mitochondrial function and induce cachexia-like phenotypes in primary hepatocytes and C2C12 myotubes.

**Figure 5:**
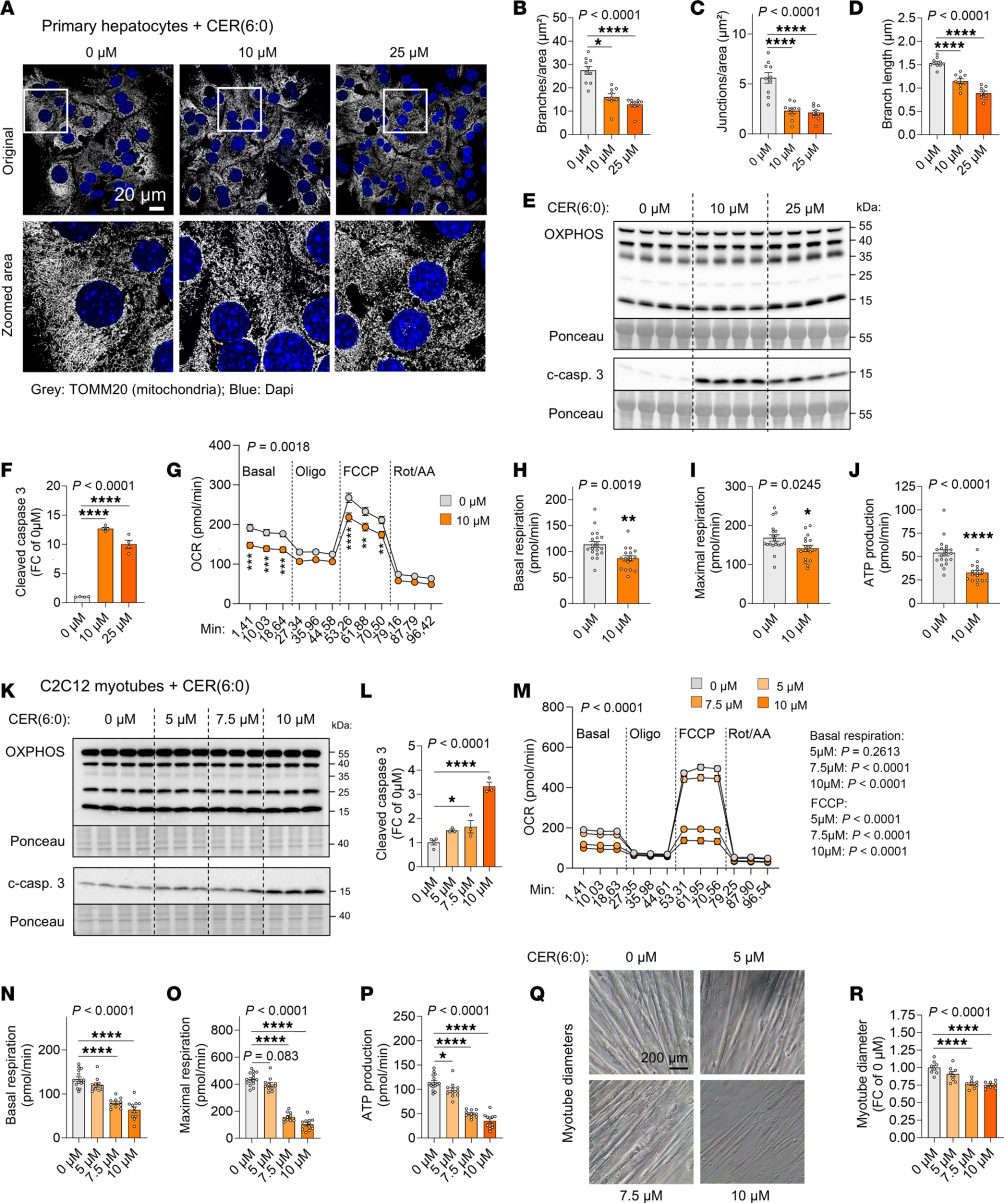
CERs directly interfere with mitochondrial function and induce cachexia-like phenotypes in primary hepatocytes and C2C12 myotubes. (**A**–**J**) Primary hepatocytes and (**K**–**R**) C2C12 myotubes were treated with CER(6:0) for 16–24 hours. (**A** and **B**) Confocal microscopy of mitochondria (data are from 6 replicates; *n* = 9–10 images quantified per group). Scale bars: 20 μm (**A**) and 200 μm (**Q**). Original magnification, ×60, insets, ×3.5. Quantification of mitochondrial morphology: branch numbers (**B**), junction numbers (**C**), and mean branch lengths (**D**). (**E**, **F**, **K**, and **L**) Western blots of mitochondria-related proteins: OXPHOS (Bands from top to bottom represent: CV, CIII, CIV, CII, CI) and cleaved caspase 3 (c-casp. 3). Ponceau was used as a loading control. (**E** and **K**) Representative blots. (**F** and **L**) Quantification of protein levels (relative to 0 μM condition; *n* = 3–4 replicates per group). (**G**–**J** and **M**–**P**) Mitochondrial respiratory measurements (normalized to protein content). (**G** and **M**) Oxygen consumption rate throughout the assay (hepatocytes: *n* = 17–20 replicates; myotubes: *n* = 10–15 replicates per group). FCCP, carbonyl cyanide-4-(trifluoromethoxy); Rot/AA, rotenone/antimycin; Min, minutes; Oligo, oligomycin. (**H** and **N**) Basal respiration, (**I** and **O**) maximal respiration, and (**J** and **P**) ATP production. (**Q** and **R**) Myotube diameters (*n* = 8 replicates per group). Data indicate the mean ± SEM. *P < 0.05, **P < 0.01, ***P < 0.001, and ****P < 0.0001 versus the 0 μM condition. Statistical analysis was done using 1-way ANOVA with Dunnett’s post hoc test (**C**, **D**, **F**, **L**, **O**, **P**, and **R**), Kruskal-Wallis with Dunn’s post hoc test (**B** and **N**), unpaired, 2-tailed *t* test (**H** and **I**), Mann-Whitney *U* test (**J**), or paired, 2-way ANOVA with Šídák’s (**G**) or Dunnett’s (**M**) post hoc test.

To further confirm the direct effect of CERs on mitochondrial morphology and capacity to produce energy, we treated primary hepatocytes with different doses of the short analog CER(6:0), a synthetic CER species commonly used for in vitro studies due to its better ability to cross cellular membranes (37, 38). Mitochondrial morphology was similar to that seen in livers of cachectic animals, with reduced branch and junction numbers and branch length (Figure 5, A–D). We also observed an induction of stress markers, as evidenced by cleaved caspase 3 levels (Figure 5, E and F). While OXPHOS protein levels were unchanged after 24 hours of treatment (Figure 5E), we still observed a clear decrease in both basal and maximal respiratory function and ATP production (Figure 5, G–J), further supporting that CERs can be directly responsible for the effect observed in vivo in cachectic animals (Figure 3).

Assessing the procachectic potential in other cell types, treatment of 3T3-L1 adipocytes with CER(6:0) did not alter lipolysis (i.e., glycerol release), even at a very high dose (Supplemental Figure 8, A and B), in accordance with the absence of effects of myriocin on adipose tissue wasting in vivo (Figure 2, J and K, and Supplemental Figure 4W). Given the protective effect of CER synthesis inhibition on cardiac and skeletal muscle wasting (Figure 2, L–W, Figure 4E, Supplemental Figure 4, X and Y, and Supplemental Figure 7K), we also treated C2C12 myotubes with CER(6:0). As in hepatocytes, CERs dose-dependently increased mitochondrial stress markers after 24 hours, without affecting OXPHOS protein levels (Figure 5, K and L), and dose-dependently reduced mitochondrial respiration and ATP production (Figure 5, M–P). Accordingly, CERs induced myotube atrophy in a dose-dependent manner, also indicating a direct effect of CERs on muscle wasting in cachexia (Figure 5, Q and R). Ultimately, we treated primary hepatocytes and myotubes with the naturally occurring CER(16:0) —the most enriched CER species in plasma of cachectic animals (Figure 1G, Figure 2I, and Supplemental Figure 7E) — and observed an impairment of mitochondrial function similar to that seen with CER(6:0) treatments (Supplemental Figure 9).

The above data therefore confirm the procachectic potential of CERs in hepatocytes and myotubes.

### Inhibition of CER synthesis improves mitochondrial phenotypes in skeletal muscle of cachectic animals.

**Figure 6:**
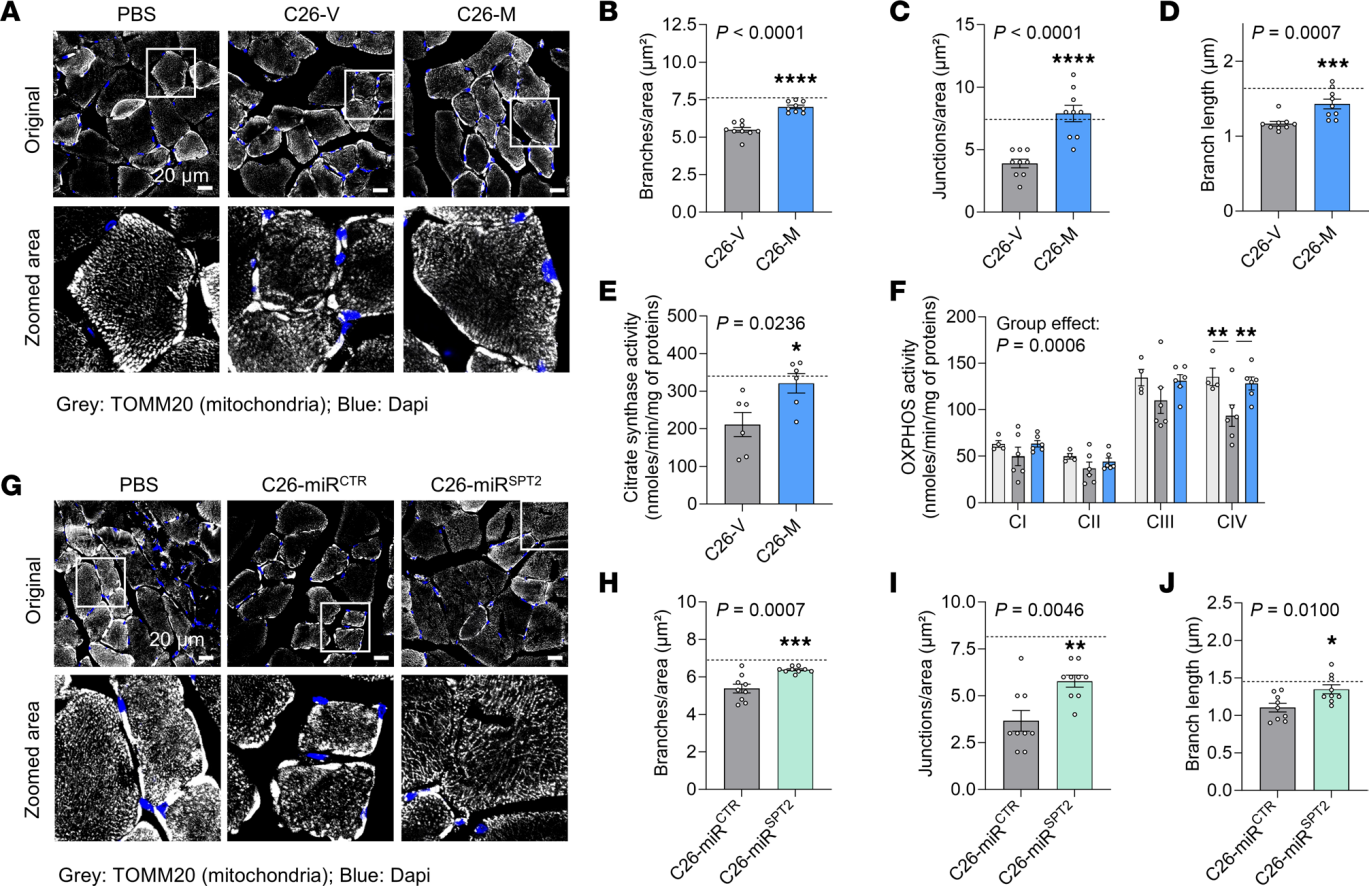
Inhibition of CER synthesis improves mitochondrial phenotypes in skeletal muscle of cachectic animals. (**A**–**F**) Mice were injected with C26 cells and treated with vehicle (C26-V) or myriocin (C26-M) (advanced cachexia experiment). Dotted lines represent the values from PBS-V–treated mice. (**A**–**D**) Confocal microscopy of mitochondria in tibialis anterior from 4 animals per group (*n* = 9 images quantified per group). Quantification of mitochondrial morphology: branch numbers (**B**), junction numbers (**C**), and mean branch lengths (**D**), indicating mitochondrial connectivity. (**E** and **F**) Citrate synthase (**E**) and OXPHOS complex (**F**) activity in gastrocnemius (*n* = 6 animals per group). (**G**–**J**) Mice were injected with an AAV expressing a control miRNA (miR^CTR^) or a miRNA against *Sptlc2* (miR^SPT2^) in a hepatocyte-specific manner, and then later with C26 cells (early cachexia experiment). Dotted lines represent the values from PBS-injected mice. Confocal microscopy of mitochondria in tibialis anterior from 4 animals per group (*n* = 9 images quantified per group). Quantification of mitochondrial morphology: branch numbers (**H**), junction numbers (**I**), and mean branch lengths (**J**), indicating mitochondrial connectivity. Scale bars: 20 μm, original magnification, ×60; insets ×3,5 (**A** and **G**). Data indicate the mean ± SEM. *P < 0.05, **P < 0.01, ***P < 0.001, and ****P < 0.0001. Statistical analysis was done using an unpaired, 2-tailed *t* test (**B**, **C**, **E**, and **H**–**J**), a Mann-Whitney *U* test (**D**), or unpaired, 2-way ANOVA with Tukey’s post hoc test (**F**).

Given the toxic effect of CERs on mitochondrial function we observed in vitro in muscle cells, we next assessed whether CERs could be involved in mitochondrial dysfunction in cachectic muscles. In myriocin-treated, tumor-bearing mice, we did not observe a strong effect of cachexia and myriocin on mitochondrial mass, function, or stress markers in gastrocnemius muscle at an early cachexia stage (Supplemental Figure 10, A–H), but we did find an unexpected increase in OXPHOS complex II (i.e., succinate dehydrogenase, also part of the TCA cycle) and stress markers (CHOP), which tend to be decreased upon myriocin treatment at advanced cachexia stages (Supplemental Figure 10, I–P). Confocal microscopic analyses of skeletal muscle revealed clear alterations of mitochondrial morphology and connectivity in cachexia, characterized by decreased mitochondrial branch and junction numbers and branch lengths (Figure 6, A–D), which were associated with an impairment of citrate synthase and activity of OXPHOS complexes (Figure 6, E and F). All those effects were partially rescued upon myriocin treatment. As validation, we also confirmed the partial rescue of mitochondrial morphology in skeletal muscle of tumor-bearing animals with liver-specific genetic knockdown of CER synthesis (Figure 6, G–J).

All in all, our data suggest that CERs affect liver mitochondrial function as an early event, promoting energy loss and muscle wasting in cachexia.

### Hepatic expression of CER synthesis enzymes and CER content are positively correlated with the severity of weight loss in patients with cancer.

**Figure 7:**
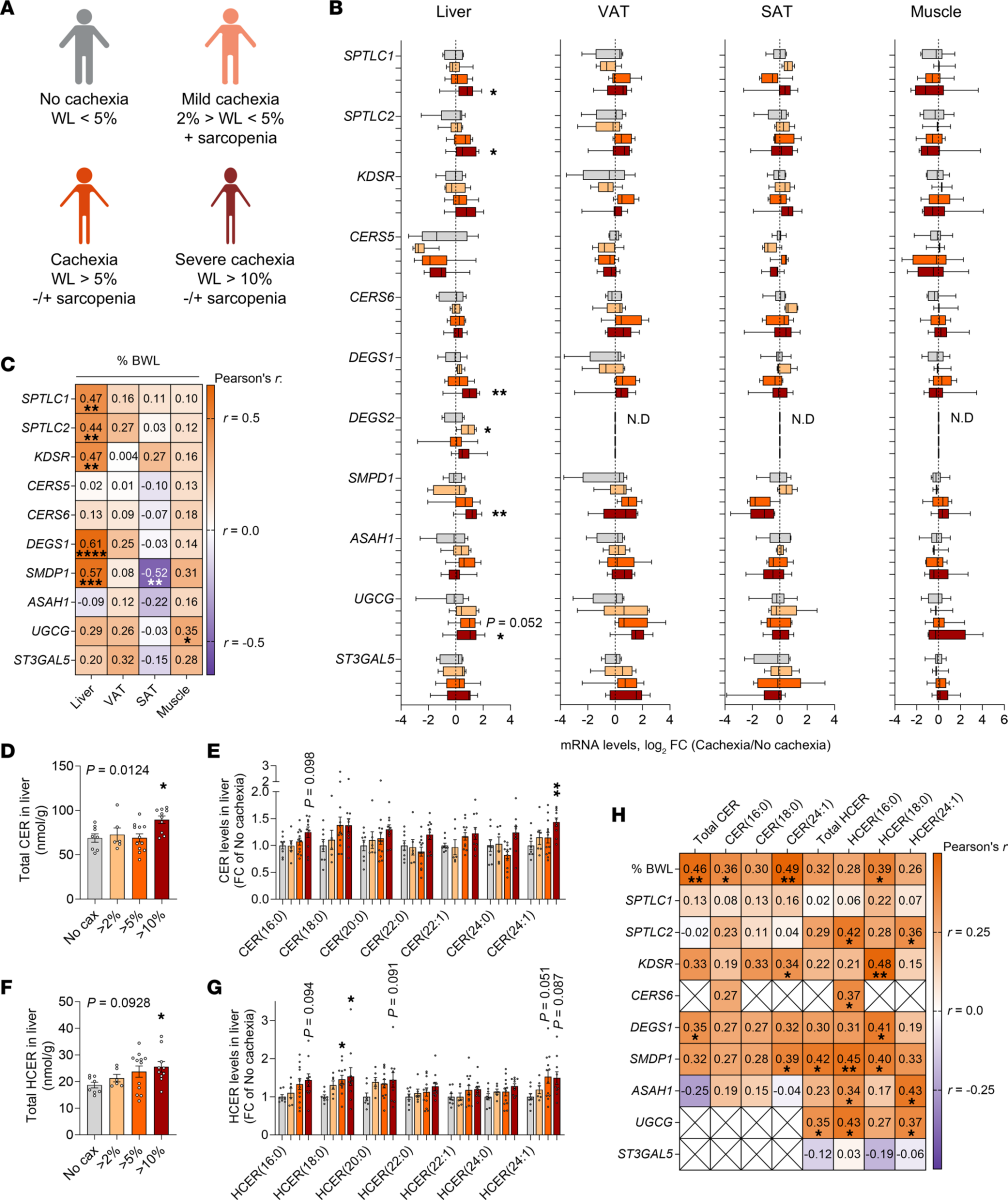
Hepatic expression of CER synthesis enzymes and CER content are positively correlated with the severity of weight loss in patients with cancer. (**A**) Patients with gastrointestinal cancer were stratified on the basis of their cachectic phenotype (no cachexia, *n* = 8 individuals; mild cachexia, *n* = 6; cachexia, *n* = 12; severe cachexia, *n* = 11; unless stated otherwise). Image created in BioRender. See also Supplemental Table 4 for patients’ characteristics. WL, weight loss. (**B**) mRNA expression of CER synthesis enzymes in liver (cachexia, *n* = 11), VAT (no cachexia *n* = 5, cachexia *n* = 8, severe cachexia *n* = 7), SAT (no cachexia *n* = 7, mild cachexia *n* = 5, cachexia *n* = 6), and skeletal muscle (mild cachexia *n* = 3, severe cachexia *n* = 10). (**C**) Correlation analyses between CER synthesis enzyme expression in liver, VAT, SAT and muscle and the percentage of body weight loss (BWL). Pearson’s *r* values are displayed for each comparison, together with their significance. (**D**–**G**) Total CER (**D**) and HCER (**F**) levels in liver (severe cachexia, *n* = 10). CER (**E**) and HCER composition (**G**) in liver (FC in the no-cachexia group). (**H**) Correlation analyses between CER and HCER levels and the percentage of body weight loss or CER synthesis enzyme expression in liver. Data indicate the median minimum to maximum (**B**) or the mean ± SEM (**D**–**G**). *P < 0.05, **P < 0.01, ***P < 0.001, and ****P < 0.0001 versus the no-cachexia group. Statistical analysis was done by 1-way ANOVA with Dunnett’s post hoc test (**B** and **D**–**G**), Kruskal-Wallis with Dunn’s post hoc test (**B**, **E**, and **G**), and correlation analysis (**C** and **H**). No cax, no cachexia.

Ultimately, we looked for evidence that hepatic CER synthesis is also a hallmark of cachexia in patients with cancer, to support a finding that could be useful for future targeted therapies. We analyzed tissue samples from patients with gastrointestinal cancers of different entities (Supplemental Table 4) and different degrees of cachexia. Cachexia was defined according to Fearon’s definition (39), and patients were stratified into 4 groups (Figure 7A and Supplemental Table 4): noncachectic, mildly cachectic, cachectic, and severely cachectic. As in our preclinical models (3) (Figure 1, A and B), we measured gene expression levels of a large panel of CER synthesis enzymes (including enzymes of the de novo synthesis and salvage pathways and glycosphingolipid synthesis) in liver, visceral adipose tissue (VAT), subcutaneous adipose tissue (SAT), and skeletal muscle from those patients (Figure 7B). Remarkably, enzymes from all synthesis pathways were significantly upregulated in the livers of patients with severe cachexia, whereas expression of these enzymes was unchanged in other metabolic tissues. Liver enzyme expression gradually increased with disease severity, although it reached significance only at the severe cachexia state. Accordingly, the association between hepatic enzyme expression and cachexia severity (i.e., percentage of body weight loss) in patients was confirmed by significant positive correlations in liver, while no significant or negative correlations were observed in other tissues (Figure 7C). To functionally confirm increased CER synthesis in livers of cachectic patients, we also performed liver lipidomics analysis of the same patients (Figure 7, D–G). Total CER and HCER levels were significantly elevated in the livers of patients with severe cachexia (Figure 7, D and F), mostly due to an increase in CER and HCER (16:0), (18:0), and (24:1) species (Figure 7, E and G). HCER and CER levels were accordingly positively associated with the severity of body weight loss in patients with cancer and with the expression of CER synthesis enzymes in the liver (Figure 7H).

In summary, our study reveals a clear role of liver-derived CER in the etiology of cachexia and highlights the strong potential and relevance of targeting CER synthesis in patients with cancer cachexia.

## Discussion

Cachexia is one of the main predictors of mortality in patients with cancer (2). Its origins are multifactorial, with many layers of complexity involving tumor- and host-secreted factors, systemic inflammation, anorexia, and metabolic reprogramming of cachexia target tissues (1, 40). Here, we report that elevated CERs were not only a hallmark of cachexia in mice and humans (3) but also contributed to the disease by affecting cellular and systemic energy homeostasis. Of note, the inclusion of only male mice is a limitation of the current study.

Inhibition of CER synthesis has been shown to be beneficial for multiple metabolic and cardiovascular diseases, with profound effects on blood pressure, insulin sensitivity, ectopic lipid accumulation, inflammation, fibrosis, and cell survival (5–7, 41). Here, we show that targeting CER synthesis using myriocin was also efficient in counteracting body wasting and muscle atrophy in cancer cachexia. We propose that CERs promote muscle loss indirectly through the control of systemic energy homeostasis by the liver, but also directly by affecting mitochondrial function in myotubes. Accumulation of CERs within muscle mitochondria has indeed been demonstrated to impair mitochondrial function by depleting coenzyme Q and disrupting respiratory chain components (42). On the other hand, inhibition of CER synthesis in skeletal muscle ameliorates mitochondrial dysfunction in age-induced sarcopenia, thereby improving functional muscle outputs (9). Mitochondrial dysfunction is a hallmark of cachexia-target tissues (1, 43–45), and our data showing that CERs interfered with mitochondrial morphology and function in the livers of cachectic mice are in accordance with previous reports in which CERs, especially CERS6-derived CER(16:0), promote mitochondrial fission, liver steatosis, inflammation, and systemic insulin resistance under obesogenic diets (14, 16). Elevated CER(16:0) levels may, at least in part, be driven by stress- or IL-6–induced increases in the levels of hepatic SPTLC2 and CERS6 expression, leading to the accumulation of toxic, shorter ceramide species (46).

In addition to the effects of CERs described in the present study, CER-derived sphingolipids may also contribute to cachexia etiology. Recent studies have shown that CER precursors, namely dihydro-CERs, are particularly toxic, promoting ER stress and age-induced sarcopenia (8, 24). HCER and LCER are precursors of more complex gangliosides, which have been shown to directly interact with insulin receptor function in plasma membranes of adipocytes, promoting insulin resistance (47). CER can also be degraded into sphingosine by acid ceramidases (e.g., ASAH1) and phosphorylated by sphingosine kinases (SPHKs) to generate sphingosine-1-phosphate (S1P), a potent signaling molecule with multiple, sometimes antagonizing, effects on metabolic tissues. S1P notably induces lipid synthesis and accumulation in liver, lipolysis in adipose tissue, chemokine and cytokine production, and immune cell infiltration in skeletal muscle and in the aforementioned tissues (5). As sphingosine is derived from CER degradation, myriocin can also reduce S1P levels by inhibiting CER synthesis.

CERs may therefore represent promising therapeutic targets for the treatment of cancer cachexia. We have shown that CER levels are elevated in patients with cancer cachexia and positively correlate with the severity of body wasting, highlighting the relevance of targeting this pathway for such patients. Historically, myriocin (SPT inhibitor) and fumonisins (CERS inhibitors) have been widely used in preclinical studies (5–9, 13, 41, 48, 49), but their long-term toxicity has precluded them from being used in the clinic. While the development of SPT inhibitors is complicated by its crucial role on gut architecture, the development of inhibitors of other enzymes of the de novo pathway, notably of CERS, is an active area of research (26, 50, 51). This may hold promise for cachexia considering the significant increase in toxic CER6-derived CER(16:0) levels in both cachectic mice and patients. Tricyclic antidepressants (desipramine, imipramine) are inhibitors of the salvage pathway, however, targeting this pathway alone may not be sufficient to counteract CER synthesis in cachexia, based on our study (51). The DEGS1 inhibitor fenretinide has shown promising results for the treatment of insulin resistance and associated liver diseases in preclinical studies (52). This drug is currently tested in clinical trials as an anticancer therapy and could represent a candidate for counteracting cancer cachexia as well (11, 12). Fingolimod is the only drug targeting sphingolipid signaling approved by the FDA for the treatment of multiple sclerosis (51). Fingolimod is a derivative of myriocin, however it does not inhibit the SPT enzyme, and its antiinflammatory effects are mediated by S1P receptor antagonization. In addition, other inhibitors of SPHKs and S1P are investigated in clinical trials for the treatment of some cancers and have been reported to be well tolerated by patients so far (ABC294640, safingol) (11, 12).

In conclusion, future studies should determine the contribution of other CER-derived sphingolipids to cachexia development, in addition to the direct effects of CERs we describe here. The development of drugs targeting de novo CER synthesis may hold promise for targeting different pathological pathways, including CERs and their derivatives, glycosphingolipids and S1P, all at once. As CER synthesis induction is one therapeutic approach under investigation to cause cancer cell death (11, 12), the benefit/risk ratio of manipulating this pathway, between counteracting tissue wasting and promoting tumor growth, should be carefully assessed, although we did not notice any effect of myriocin on tumor growth here. Therapies may be combined with diets aimed at reducing CER levels, including diets low in total fat and enriched in unsaturated fatty acids, and antiinflammatory drugs (5). Overall, our study provides proof of concept of the potential of targeting CER metabolism for the treatment of cancer cachexia and paves the way for future studies using combined approaches and currently available drugs suitable for clinical use to target sphingolipids.

## Methods

### Sex as a biological variable

We used only male mice to ensure consistent experimental conditions and reduce variability. This choice reflects reported sex differences in patients with cancer-associated weight loss, with male individuals showing greater susceptibility. In mouse models, sex-specific regulation may also affect responses to cachexia-inducing cytokines during disease progression. Therefore, limiting experiments to male mice improved the precision of our analysis by accounting for potential sex-specific disease features. Patient data include both men and women.

### Animal experiments

In vivo experiments were carried out in 9.5- to 11-week-old male CD2F1, BALB/c, and C57BL6/J mice from Charles River Laboratories. Mice were maintained under specific pathogen–free conditions on a 12-hour light/12-hour dark cycle at 22°C with ad libitum access to chow (Altromin, no. 1314) and water. Mice were assigned to groups, such that initial body weights and grip strength were similar between the groups, as confirmed by nonsignificant statistical analysis.

#### Cachexia experiments.

For early cachexia experiments, mice were injected subcutaneously with 1 million C26 cells (mouse colon carcinoma) or a similar amount of PBS (Thermo Fisher Scientific, 14190250). Mice were monitored for 2–4 weeks after cell implantation with daily assessment of tumor growth, body weight, and the body condition score (BCS). Mice were sacrificed by ketamine/xylazine overdose or cervical dislocation once they developed cachexia (body weight loss >8%–10% of the initial body weight, early cachexia experiments).

#### Generation of AAV vectors, AAV production, and AAV purification and titration.

Details can be found in Supplemental Methods.

#### In vivo Sptlc2 silencing.

Animals were injected with 2 × 10^11^ virus particles via the intravenous route (i.e., tail vein). After 2 weeks of incubation, the animals were injected with C26 cancer cells, and the cachexia experiment was conducted as described above.

#### CER synthesis inhibition by myriocin in early cachexia.

PBS- or C26 tumor–bearing mice received a daily dose of 0.3 mg/kg myriocin (MilliporeSigma, M1177) or a similar volume of vehicle (saline) intraperitoneally as soon as the tumor was palpable and until the end of the experiment.

#### Assessment of grip strength.

Muscle strength was recorded at the beginning and end of the experiment using a BIO-GS3 device (Bioseb Instruments). Mice were positioned with the forelimbs on the grid connected to a dynamometer, and the force was recorded after pulling the animal’s tail. Measurements were carried out 3 times in succession, with resting times between recordings, and the mean of all values was used.

#### Metabolic cages.

Details are provided in Supplemental Methods.

#### Assessment of insulin sensitivity.

Details are provided in Supplemental Methods.

#### Fasting experiment.

C57BL/6 mice had either free access to food (i.e., ad libitum group) or were fasted for 24 hours (i.e., fasting group) before necropsy.

#### LPS experiment.

Animals were injected intraperitoneally with a single dose of LPS (1 mg/kg body weight, Merck Millipore, LPS25) diluted in PBS. Animals were necropsied 18 hours later. Control mice were not subjected to any treatment.

#### Primary hepatocyte isolation.

Primary hepatocytes from 8- to 12-week-old male C57BL/6 mice were isolated via collagenase perfusion as previously described (53, 54). See Supplemental Methods for additional information.

#### CER synthesis inhibition by myriocin in advanced cachexia.

The experiment was conducted at the Venetian Institute of Molecular Medicine (Padova, Italy). Male CD2F1 mice (10-week-old, CRL) were housed in independent cages in an environmentally controlled room (23°C, 12-hour light/12-hour dark cycle) with ad libitum access to food and water.

As soon as tumors were palpable, PBS-treated mice and mice with C26 tumors were treated daily with 0.3 mg/mL myriocin for 1 week to induce an initial strong inhibition of CER synthesis, and then every other day to maintain the inhibition until the end of the experiment. Control mice were treated with similar amounts of vehicle. The experiment was ended once the vehicle-treated mice had lost more than 20% of their body weight. Animals were anesthetized with an overdose of ketamine/xylazine to collect blood from the retro-orbital vein and then euthanized by cervical dislocation. Muscle strength was recorded at the beginning and the end of the experiment following the same procedure as described above.

#### IL-6 neutralization.

See Supplemental Methods for details.

### Cell culture

All cell lines were tested for mycoplasma contamination by PCR according to the manufacturer’s instructions (Promokine, PK-CA91–1048).

#### C26 cancer cells.

See Supplemental Methods for details.

#### Primary hepatocytes.

See Supplemental Methods for details.

#### C2C12 myotubes.

See Supplemental Methods for details.

#### 3T3-L1 adipocytes.

See Supplemental Methods for details.

#### Treatment with CERs.

CER(6:0) (d18:1/6:0, MilliporeSigma [Avanti], 860506P) and CER(16:0) (d18:1/16:0, MilliporeSigma [Avanti],860516P) were resuspended in methanol/chloroform (50:50, v/v) as a 2.5 mM stock solution and stored at –20°C under oxygen-free conditions (nitrogen gas) as previously described (24). For cell treatment, necessary volumes of stock solution were evaporated under nitrogen gas, and CERs were resuspended in ethanol/dodecane (98:2, v/v) to obtain a 2.5 mM working solution. After heating and regularly vortexing the working solution at 37°C for 20 minutes, the appropriate volumes of working solution were diluted into media right before cell treatment. Primary hepatocytes were treated with either 10 μM or 25 μM CERs overnight for all assays. Myotubes were treated with 5 μM, 7.5 μM, or 10 μM CERs overnight for the Seahorse assay and Western blotting and for 24 hours for diameter measurement. Adipocytes were treated with 10 μM and 50 μM CERs for 24 hours for lipolysis assays. For all cell types, control cells were treated with the equivalent amount of vehicle.

#### Seahorse experiments.

Respirometry measurements were performed using the Seahorse XF Cell MitoStress Test kit and the Seahorse XF96 Analyzer (Agilent Technologies, 103792100) according to the manufacturer’s instructions. Assay media for hepatocytes consisted of XF DMEM Medium (Agilent Technologies, 103575-100) supplemented with 1.78 mM glucose (Agilent Technologies, 103577-100), and for myotubes, the media consisted of XF DMEM Medium supplemented with 10 mM glucose, 1 mM pyruvate (Agilent Technologies, 103578-100), and 2 mM l-glutamine (Thermo Fisher Scientific, 25030-024). For primary hepatocytes, we consecutively injected 2 μM oligomycin (MilliporeSigma, 579-13-5), 1 μM FCCP (Biomol, 370-86-5), 2 μM rotenone (MilliporeSigma, 83-79-4), and antimycin A (MilliporeSigma, 1397-94-0) and for C2C12 myotubes, 1 μM oligomycin, 4 μM FCCP, and 0.75 μM rotenone/ antimycin A. The following settings were applied: 3 cycles per condition, 3 minutes’ mixing time, 2 minutes’ waiting time, and 3 minutes’ measurement time. OCR data were normalized by relative protein content, determined using the Pierce BCA assay (Thermo Fisher Scientific, 23225). The experiment was repeated using different independent cultures of hepatocytes and myotubes.

#### Myotube diameter.

After 24 hours of treatment, 4 images of myotubes per well were recorded (×40 objective, Nikon Eclipse Ts2). Approximately 20 myotubes per well were quantified using ImageJ software (NIH), and the average was considered as 1 independent biological replicate. The experiment was repeated using different independent cultures of myotubes.

#### Adipocyte lipolysis.

See Supplemental Methods for details.

### Patient data

This study included both male and female patients. Most patients were from Germany, with some participants from other European countries. All participants self-identified as White. No further categorization on the basis of race, ethnicity, or other socially relevant groups was performed. Upon recruitment prior to surgery, the patients’ clinical characteristics were collected through standardized questionnaires, anthropometry, routine clinical chemistry, and medication (MUCABI [Munich Cachexia Biomarkers Working Group]). The skeletal muscle area index, a marker of sarcopenia, was evaluated in all patients as described previously (35, 55). Sarcopenia was defined as a skeletal muscle area index of less than 52.4 cm^2^/m^2^ for men and less than 38 cm^2^/m^2^ for women (56). Detailed patient characteristics are summarized in Supplemental Table 4. Patients were stratified according to Fearon’s definition of cachexia (39) and divided in the following groups: (a) no cachexia, i.e., patients with weight loss of up to 5% in the previous 6 months without sarcopenia or patients with sarcopenia but with less than 2% weight loss; (b) mild cachexia, i.e., patients with weight loss of 2%–5% with sarcopenia; (c) cachexia, i.e., patients with 5%–10% weight loss with or without sarcopenia; and (d) strong cachexia, i.e., patients with weight loss of greater than 10% with or without sarcopenia.

Samples of liver (from liver segments III, IVb, or V, where, macroscopically, no liver lesions were seen), abdominal VAT, SAT, and musculus rectus abdominis were collected from a total of 37 patients with malignant diseases of the gastrointestinal tract during surgical procedures at the Department of Surgery, Klinikum rechts der Isar (University Hospital of the Technical University of Munich, Munich, Germany). Tissue samples were snap-frozen and stored at –80°C until further analysis.

### Real-time quantitative PCR and Western blot analysis

See Supplemental Methods for details.

### Microscopy analysis

#### Mitochondria 3D reconstruction and morphometric analysis by confocal microscopy.

Liver pieces or whole tibialis anterior muscles were fixed in 4% paraformaldehyde (Roth, P087.5) for 24 hours at 4°C. Samples were then washed in PBS and switched to a 30% sucrose (MilliporeSigma, S1888) and PBS solution for another 48 hours at 4°C. Fixed tissues were then washed in PBS before embedding in O.C.T. compound (VWR, 361603E). Liver slices (6 μm) and muscle slices (8 μm) were cut using a cryostat (Leica, CM1860) and attached on a glass slide (SuperFrost plus slide, VWR, 631-0108). We then proceeded immediately to antibody staining with the following steps: incubation in 0.1% glycine (MilliporeSigma, G7126) and PBS for 5 minutes; permeabilization in 0.1 % Triton X-100 (Roth, 3051.4) and PBS for 10 minutes; blocking in 3% BSA (MilliporeSigma, A7030) and PBS for 15 minutes; incubation in primary antibody (TOMM20, Abcam, ab78547, 1:300) diluted in 3% BSA and PBS for 2 hours in a wet chamber; 3 washes in 3% BSA and PBS for 5 minutes; incubation in a secondary antibody (goat–anti rabbit Alexa 555, 1:1,000, Thermo Fisher Scientific, A-21429) diluted in 3% BSA and PBS for 45 minutes in a wet chamber in the dark; 3 washes in DAPI (Life Technologies, Thermo Fisher Scientific, D3571) and PBS (1:5,000) solution for 5 minutes in the dark; and a last wash in PBS. All steps were carried out at room temperature. Slides were then mounted using Mowiol (Merck Millipore, 475904). Negative controls consisted of staining only with the secondary antibody to assess fluorescence background.

For primary hepatocytes in the monolayer, we used an alternative protocol to the one previously described with the following modifications: incubation in 4% paraformaldehyde for 15 minutes; 2 washes in PBS; 0,1 % Triton X-100 and PBS for 5 minutes; 3 washes in PBS; 10 % horse serum (Life Technologies, Thermo Fisher Scientific, 16050130) for 10 minutes; primary antibody diluted in 5 % horse serum and PBS for 1 hour; 3 washes in PBS; secondary antibody diluted in 5 % horse serum and PBS for 1 hour; 3 washes in DAPI and PBS; and mounting with Mowiol.

Immunofluorescence samples were analyzed using a laser-scanning confocal microscope (Olympus Fluoview 1200; Olympus) equipped with an Olympus UPlanSApo ×60 (1.35 NA) and UPlanSApo ×40 (1.25 NA) silicone oil immersion objective (Olympus). Images were acquired at a resolution of approximately 100 nm/pixel (×60 objective) with a *z*-step size of 600 nm.

For mitochondrial 3D reconstruction, images were deconvolved using the FIJI plugins point spread function (PSF) generator (57) and DeconvolutionLab (58) (EPFL; http://bigwww.epfl.ch/). *Z*-step was set to 0.6 mm, and a PSF algorithm (Born & Wolf 3D Optical model) was used for PSF generation. The generated PSF and a 3D deconvolution algorithm (Richardson-Lucy with TV regularization) were applied to microscopic images using DeconvolutionLab. From the deconvolved 2D and 3D binary images (8 bit images), the mitochondrial network was determined by generating a skeleton of the images using the Fiji plugin Skeletonize3D and analyzed using the plugin AnalyzeSkeleton (2D/3D). This plugin will tag all pixel/voxels in a skeleton image and then counts the junctions and branches of the mitochondrial network and measures their average length. For mitochondrial network analysis, at least 3 images per mouse with more than 30 hepatocytes per image were analyzed.

#### Determination of mitochondrial morphology by electron microscopy.

Electron microscopy was performed at the CCMA EM Core Facility (Université de Nice Sophia Antipolis, Nice, France). Liver pieces were fixed in 2.5% glutaraldehyde (Science Services, E16220) and postfixed for 2 hours in 1% osmium tetroxide and 1% potassium ferrocyanide in 0.1 M cacodylate buffer pH 7.4 to enhance the staining of membranes. Samples were then rinsed in distilled water (overnight at 4°C), dehydrated in acetone on ice, and embedded in epoxy resin. Several vacuum steps to remove air bubbles were performed. Classically contrasted ultrathin sections (70 nm) were analyzed under a JEOL 1400 transmission electron microscope equipped with a Morada Olympus CCD camera or with GATAN-RIO9. Quantification of mitochondria morphology in the electron microscopy images was obtained using Fiji software (ImageJ, version 2.0.0-rc-69/1.52p). Briefly, the morphological descriptors (roundness, circularity, area, perimeter) were extracted by combining the “freehand selections” tool to perform manual segmentation of the mitochondria, with the “measure” tool to collect the quantitative values for each mitochondrion. The mitochondrial form factor was estimated as follows: (Perimeter^2^)/(4π × area).

### Isolation of crude mitochondria for lipidomics

Details are provided in Supplemental Methods.

### Determination of CER levels

See Supplemental Methods for details.

### Proteomics

Details can be found in Supplemental Methods.

### Measurement of citrate synthase and OXPHOS complex activity

See Supplemental Methods for details.

### Serum analyzer, plasma glycerol and NEFA levels, SAA and IL-6 ELISAs

Details are provided in Supplemental Methods.

### Statistics

Unless stated otherwise in the different Methods sections (e.g., *Proteomics*), statistical analysis (except ANCOVA) was performed using GraphPad Prism 10 (GraphPad Software). Normality was tested using the Shapiro-Wilk normality test. Statistical tests were 2 sided. Unpaired, 2-tailed, nonadjusted Student’s *t* tests and Mann and Whitney tests were performed to compare 2 conditions. Unpaired, 1-way ANOVA, nonadjusted, with Tukey’s or Dunnett’s post hoc tests or Kruskal-Wallis with Dunn’s post hoc tests were applied to compare more than 2 groups. Paired or unpaired 2-way ANOVA with Tukey’s, Šídák’s, or Dunnett’s post hoc tests were used to compare 2 variables. Correlation analysis was performed to test associations between 2 variables with a CI of 95%.

ANCOVA was performed to evaluate the effect of group on tissue weights, while adjusting for initial body weight. Assumptions of linearity, homogeneity of slopes, homogeneity of variances (Bartlett’s test), and normality of residuals (Shapiro-Wilk test) were verified prior to analysis. When necessary, variables were log transformed to meet model assumptions (Supplemental Table 1: eWAT, iWAT; Supplemental Table 2: eWAT). Analyses were performed using R 4.4.1 and the The R Stats Package (version 4.4.1).

A *P* value of less than 0.05 was considered significant. Statistical tests applied for each panel with corresponding *P* values can be found in the figure legends and the Supporting Data Values file. Data are presented as the mean ± SEM, or minimum to maximum median values.

### Study approval

#### Early cachexia experiments, fasting experiment, LPS experiment, and primary hepatocyte isolation.

Animal handling and experiments were performed in accordance with the institutional animal welfare officer and licenses from the state ethics committee and Government of Upper Bavaria (ROB-55.2-2532.Vet_02-18-93, ROB-55.2-2532.Vet_02-22-47, ROB-55.2-2532.Vet_02-19-156, ROB-55.2-2532.Vet_02-21-66).

#### IL-6 neutralization.

This experiment was approved by and performed in accordance with the guidelines of the local ethics committee from the UCLouvain, Belgium. Housing conditions were as specified by the Belgian Law of May 29, 2013, regarding the protection of laboratory animals.

#### Advanced cachexia experiment.

This experiment was conducted at the Venetian Institute of Molecular Medicine (Padua, Italy). The study was conducted according to the NIH’s *Guide for the Care and Use of Laboratory Animals* (National Academies Press, 2011), the ARRIVE guidelines (https://arriveguidelines.org/), as well as the Italian law for the welfare of animals. The Italian Ministero della Salute approved all animal experiments, Allegato VI (Rome, Italy; authorization no. 448/2021 PR).

#### Patient data.

The study was approved by the ethics committee of the Medical Faculty of the Technical University of Munich (project number 409/16 S) and is registered in the German Clinical Trials Register (Deutsches Register Klinischer Studien, identifier DRKS00017285). All patients provided written informed consent before participation in this study, and the conduct of this study complied with the Declaration of Helsinki.

### Data availability

Raw data and the statistical analyses performed can be found in the Supporting Data Values file. The mass spectrometry proteomics data have been deposited in the ProteomeXchange Consortium via the PRIDE (59) partner repository with the dataset identifier PXD062072. Further information can be found in Supplemental Methods.

## Author contributions

MR and PM conceptualized, managed, and coordinated the project. PM designed the animal and cell experiments, analyzed and interpreted data. PM, BB, LC, SZ, LN, DK, TFS, and LBB performed in vivo experiments. PM and A Maier performed in vitro experiments. HJ, RTE, SQG, JJT, and VK assisted with the in vivo and in vitro experiments. YK, AZ, SLG, and JG performed, analyzed, and interpreted confocal and electron microscopy analyses. CFV and SC performed, analyzed, and interpreted mitochondria activity assays. FR and MH performed and analyzed lipidomics. CVT performed and analyzed proteomics. TK, CS, OP, JB, MC, and HH coordinated patient recruitment and clinical characterization, provided patient tissue samples, and performed gene expression analyses. PM, A Maida, and AT developed and produced AAV vectors. BB, LBB, OK, MBD, and SH contributed valuable discussions and suggestions. PM prepared the figures. PM and MR wrote the manuscript. All authors edited the manuscript and agreed to the final version.

## Conflict of interest

The authors have declared that no conflict of interest exists.

## Funding support

- European Research Council (ERC) under the European Union’s Horizon 2020 research and innovation program (STOPWASTE no. 949017) (to MR).
- Helmholtz Association Initiative and Networking Fund (to MR and SH).
- German Diabetes Center (DZD) Next grant (to MR).
- EFSD/Novo Nordisk Foundation Future Leaders Award (to MR).
- German Center for Cardiovascular Research (DZHK) excellence grant (to MR).
- German Centers for Health Research (DZG) innovation fund (to MR).
- German Research Foundation (DFG FOR5795-1_HyperMet, to MR).
- HORIZON-MSCA-2024-PF-01 (project 101202047, to PM).
- Joint grant of the Bavarian-Czech Academic Agency (BTHA-JC-2022-3)/Czech Ministry of Education, Youth and Sports (LUAUS24040, to SH and OK).
- Else-Kröner-Fresenius-Stiftung (2020 EKSE.23, to SH and MBD)
- Edith-Haberland-Wagner Stiftung (to SH and MBD).
- Collen-Francqui Research Professor, Francqui Fondation (to LBB).
- Walloon Region strategic axis FRFS-WELBIO (40009849, to LBB).
- Fonds Wetenschappelijk Onderzoek – Vlaanderen (FWO) (to LBB).
- Fonds de la Recherche Scientifique FNRS (EOS project no. 40007505, to LBB).
- AIRC Foundation for Cancer Research (project 27007, to BB).
- HORIZON-MSCA-2023-PF-01 (project 101154890, to LC).
- Clinical Leave Stipend from the German Center of Infection Research (DZIF, grant TI07.001”, to OP).
- Novo Nordisk Foundation (NNF21SA0072102, to MC).
- Weissman, Davis and Titlebaum Family MGH Research Scholar 2024-2029 (to MC).
- Else-Kröner-Fresenius-Foundation, Bad Homburg, Germany (to TK).
- INSERM, Université Côte d’Azur, Adipo-Cible Research Study Group (supported by the France 2030 investment plan, to JG).
- French National Research Agency (ANR) through the Investments for the Future Labex SIGNALIFE (ANR-11-LABX-0028-01, to JG).
- Université Côte d’Azur Joint Excellent and Dynamic Initiative (ANR-15-IDEX-01, to JG).
- Agence Nationale de la Recherche Projet de Recherche Collaborative program (ANR-23-CE14-0048-01, to JG).

## Supplementary Material

Supplemental data

Unedited blot and gel images

Supporting data values

## Version 1 05/15/2026

Electronic publication
