# Supplementary material for: Inhibition of ceramide synthesis ameliorates body wasting in a cancer cachexia model
Source: J Clin Invest. 2026 May 15;136(10):e194687. doi: 10.1172/JCI194687 (PMC13178653; doi:10.1172/JCI194687)
Supplement: Supplemental data [file jci-136-194687-s037.pdf]

Supplemental materials

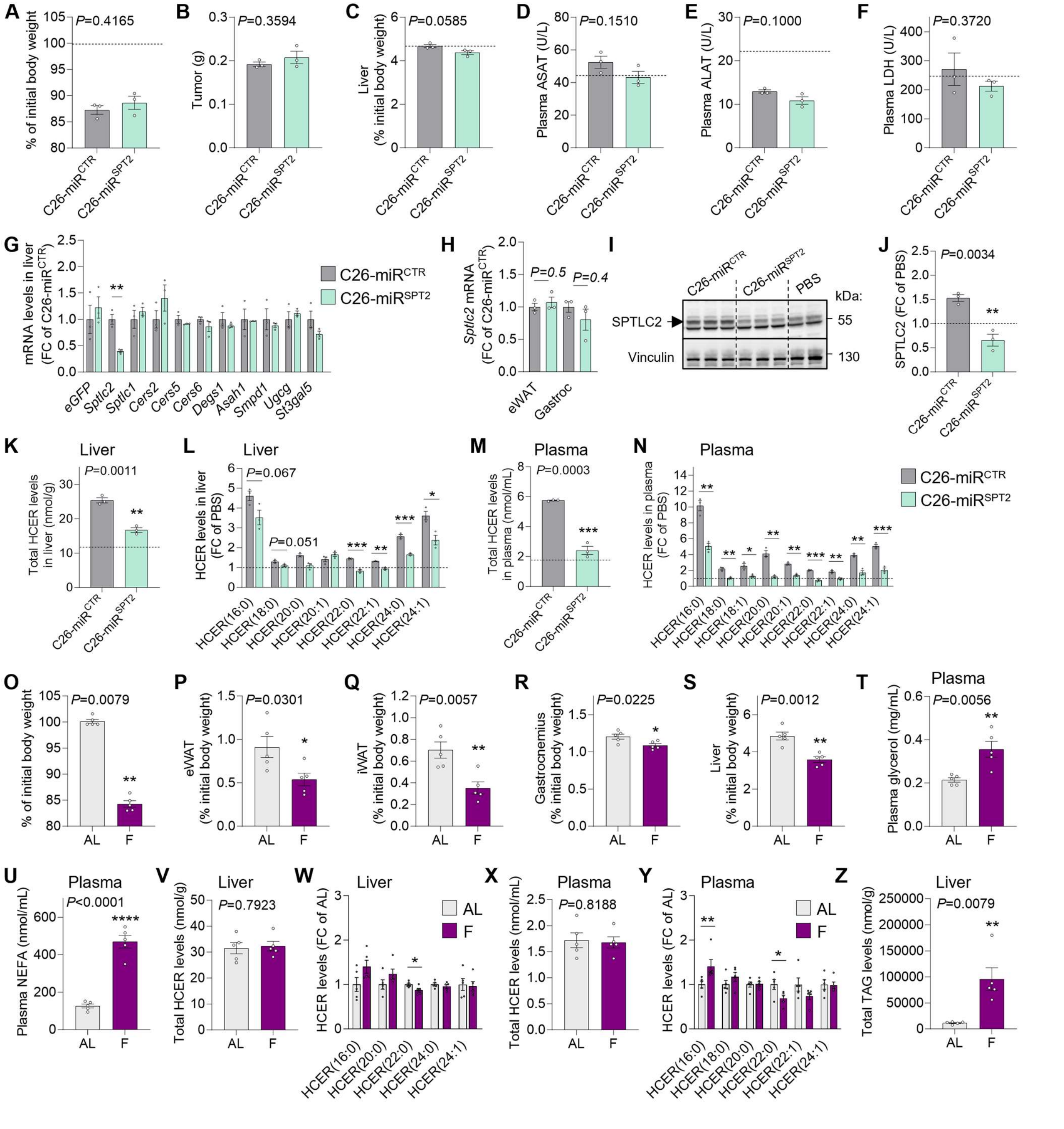

Supplemental Figure 1. Liver is responsible for the increased circulating levels of CERs in cachexia. (A-N) Mice injected with hepatocyte-specific AAVs expressing either a control miRNA (miR^{CTR}) or a miRNA against *Sptlc2* (miR^{SPT2}), and with cachexia-inducing C26 carcinoma cells. Experiment ended once each animal had reached 10% weight loss for comparable cachexia severity (n=3 animals per group). Horizontal dotted lines show reference levels

of healthy controls (PBS-injected mice). **(A)** Body weight loss. **(B)** Final tumor weight. **(C)** Liver weight. **(D-F)** Markers of liver toxicity: plasma levels of ASAT **(D)**, ALAT **(E)**, and lactate dehydrogenase **(F)**. **(G)** mRNA expression of *eGFP* (positive control of transgene expression) and CER synthesis enzymes in liver. **(H)** mRNA expression of *Sptlc2* in epididymal adipose tissue and gastrocnemius muscle. **(I** and **J)** Protein levels of SPTLC2 in liver. Vinculin as loading control. Quantification relative to PBS. **(K-N)** Total HCER levels **(K, M)** and HCER composition relative to PBS controls (**L, N**) in liver **(K** and **L)** and plasma **(M** and **N)**. **(O-Z)** Mice were fasted for 24 h to induce a weight loss > 10% similar to cachexia (F, fasting) or fed *ad libitum* (AL, controls) (n=5 animals per group). **(O)** Body weight loss. **(P** and **Q)** Epididymal **(P)** and inguinal **(Q)** adipose tissue weights. **(R)** Skeletal muscle weight (gastrocnemius). **(S)** Liver weight. **(T** and **U)** Plasma glycerol **(T)** and non-esterified fatty acid levels **(U)**. **(V-Y)** Total HCER levels **(V, X)** and HCER composition relative to AL controls (**W, Y**) in liver **(V** and **W)** and plasma **(X** and **Y)**. **(Z)** Liver triacylglycerol levels. Data are mean ± s.e.m. *$P < 0.05$, ** $P < 0.01$, *** $P < 0.001$, **** $P < 0.0001$. Statistical analysis: unpaired two-tailed *t* test (A-D, F-H, J-N, P-Y) or Mann and Whitney's test (E, H, L, O, W, Y-Z).

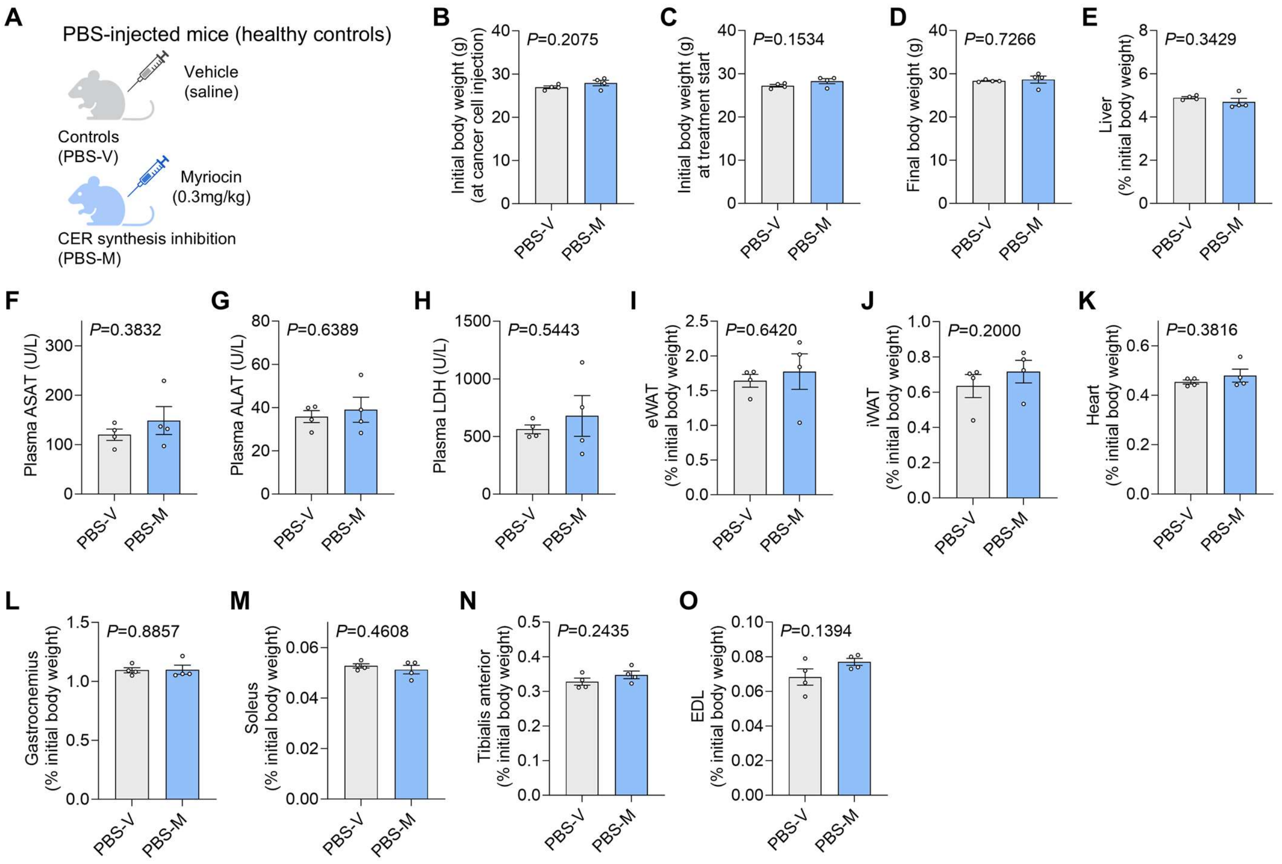

Supplemental Figure 2. **Pharmacological CER synthesis inhibition did not induce liver toxicity in tumor-free animals. (A)** Mice injected with PBS (no tumor) and treated with vehicle (PBS-V) or myriocin (PBS-M) for a similar amount of time as C26 tumor mice with advanced cachexia (see also **Figure 2**) (n=4 animals per group). **(B** and **C)** Initial body weight at the start of the experiment (injection of PBS, **B**), and at the start of myriocin treatment **(C)**. **(D)** Final body weight. **(E)** Liver weight. **(F-H)** Markers of liver toxicity: plasma levels of ASAT **(F)**, ALAT **(G)**, and lactate dehydrogenase **(H)**. **(I** and **J)** Epididymal (**I**, 2 depots) and inguinal (**J**, 1 depot) adipose tissue weights. **(K)** Heart weight. **(L-O)** Skeletal muscle weights: gastrocnemius **(L)**, soleus **(M)**, tibialis anterior **(N)** and extensor digitorum longus (EDL) **(O)**. Data are mean ± s.e.m. Statistical analysis: unpaired two-tailed *t* test (B-D, F-I, K, M-O) or Mann and Whitney's test (E, J, L).

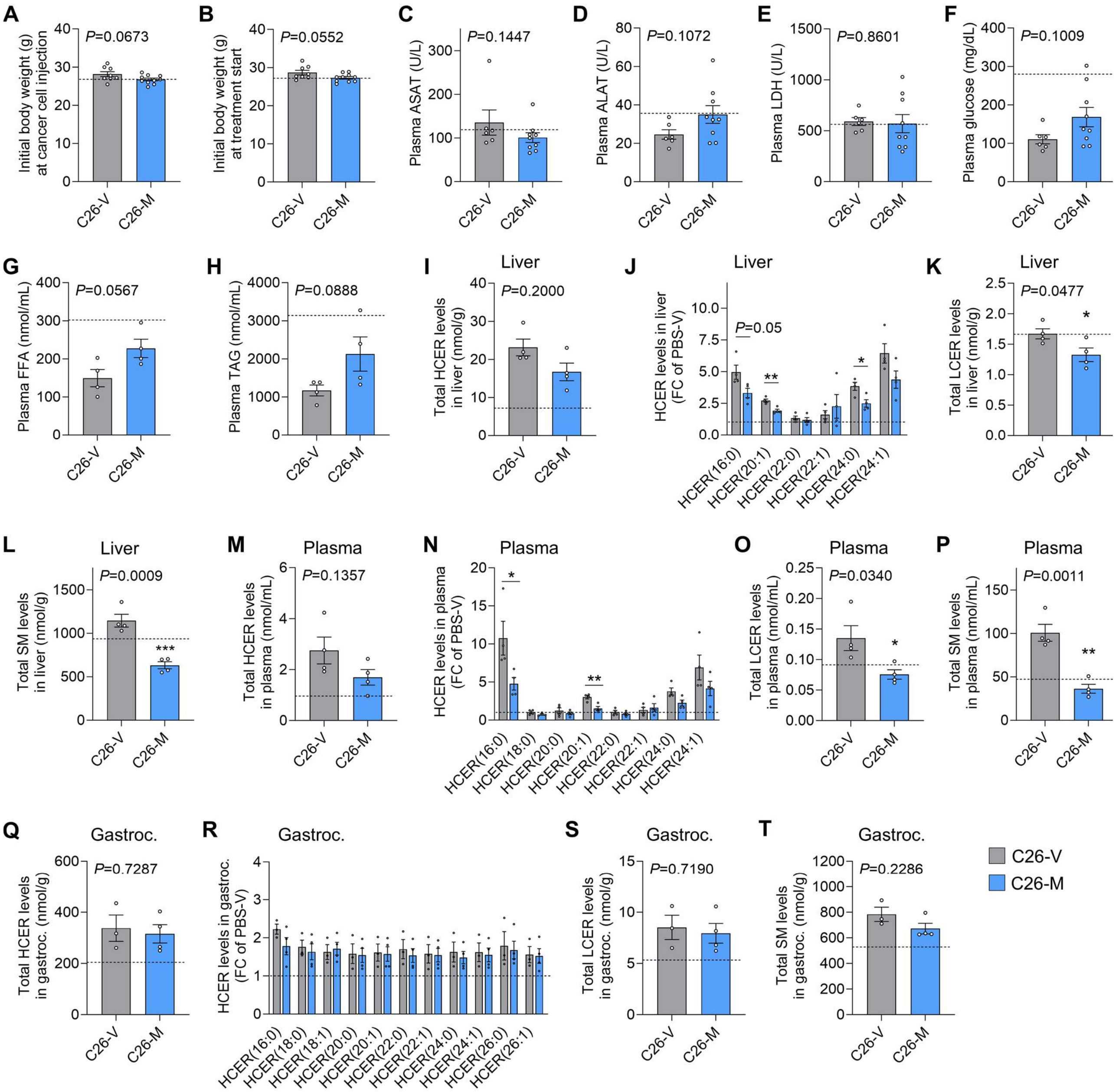

Supplemental Figure 3. Pharmacological CER synthesis inhibition did not induce liver toxicity in tumor-bearing animals. Mice injected with C26 cells and treated with vehicle (C26-V, n=8 animals, unless stated otherwise) or myriocin (C26-M, n=10 animals, unless stated otherwise). Experiment ended once control animals lost > 20 % of their initial body weight (**advanced cachexia experiment**, see also **Figure 2**). Horizontal dotted lines show reference levels of healthy controls (PBS-injected, vehicle-treated mice). **(A and B)** Initial body weight at the start of the experiment (injection of C26 cancer cells, **A**), and at the start of myriocin treatment **(B)**. **(C-E)** Markers of liver toxicity: plasma levels of ASAT **(C)**, ALAT **(D)**, and lactate dehydrogenase **(E)** (C26-V: n=6; C26-M: n=9 animals).

(F) Plasma glucose levels (C26-V: n=6; C26-M, n=9 animals). **(G** and **H)** Plasma free fatty acid **(G)** and triacylglycerol levels **(H)** (n=4 animals per group). **(I-L)** Total HCER **(I)**, LCER **(K)** and SM **(L)** levels in liver, and liver HCER composition relative to PBS-V controls **(J)** (n=4 animals per group). **(M-P)** Total HCER **(M)**, LCER **(O)** and SM **(P)** levels in plasma, and plasma HCER composition **(N)** (n=4 animals per group). **(Q-T)** Total HCER **(Q)**, LCER **(S)** and SM **(T)** levels in gastrocnemius, and HCER composition in gastrocnemius **(R)** (C26-V, n=3; C26-M, n=4 animals). Data are mean ± s.e.m. $^*P < 0.05$, $^{**} P < 0.01$. Statistical analysis was performed using unpaired two-tailed *t* test (A-B, D-H, J-S) or Mann and Whitney's test (C, I, N, T).

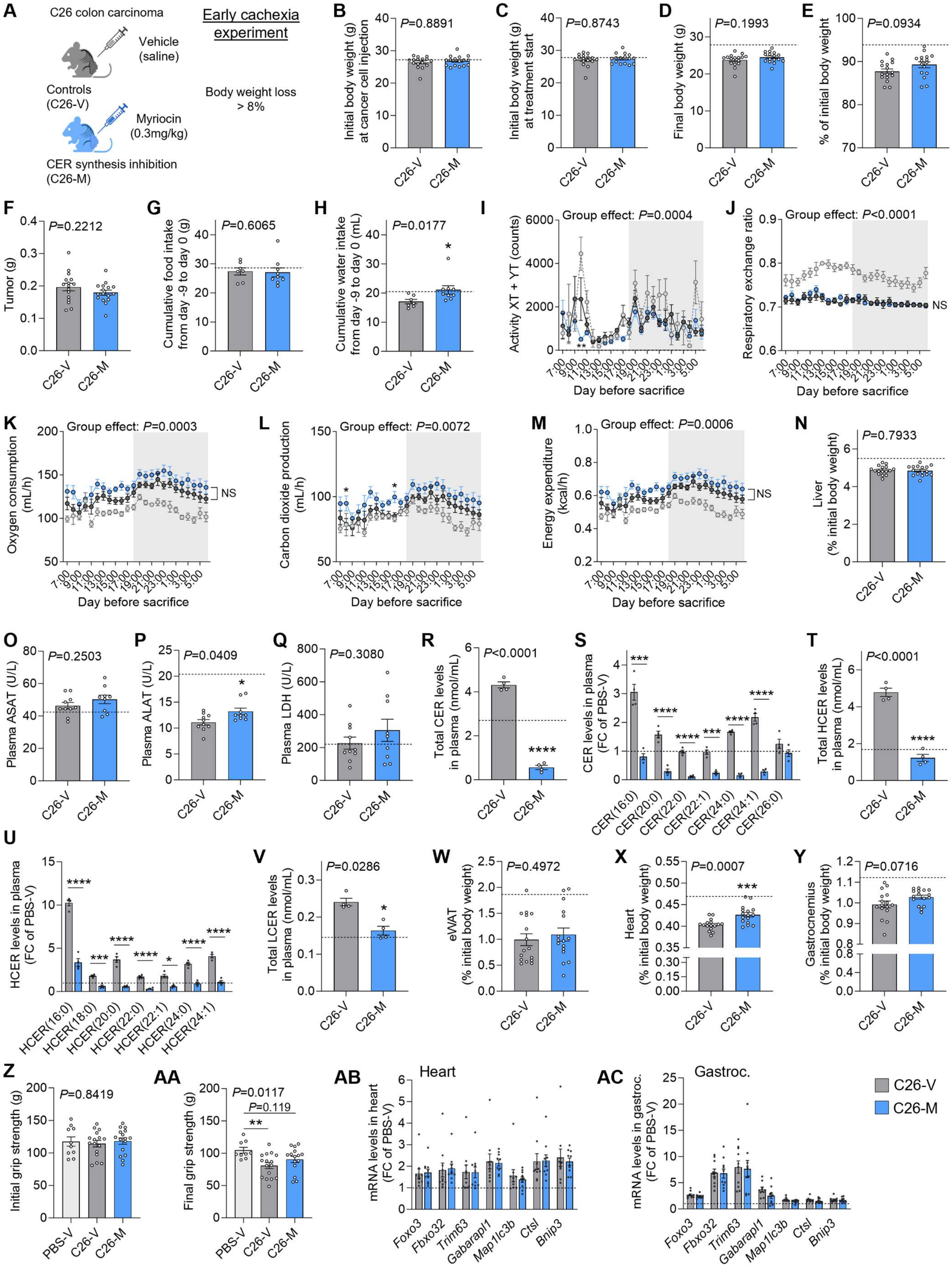
A
C26 colon carcinoma
Vehicle (saline)
Controls (C26-V)
Myriocin (0.3mg/kg)
CER synthesis inhibition (C26-M)
Early cachexia experiment
Body weight loss > 8%
B
P=0.8891
Initial body weight (g) at cancer cell injection
C
P=0.8743
Initial body weight (g) at treatment start
D
P=0.1993
Final body weight (g)
E
P=0.0934
% of initial body weight
F
P=0.2212
Tumor (g)
G
P=0.6065
Cumulative food intake from day -9 to day 0 (g)
H
P=0.0177
Cumulative water intake from day -9 to day 0 (mL)
I
Group effect: P=0.0004
Activity XT + YT (counts)
Day before sacrifice
J
Group effect: P<0.0001
Respiratory exchange ratio
NS
Day before sacrifice
K
Group effect: P=0.0003
Oxygen consumption (mL/h)
NS
Day before sacrifice
L
Group effect: P=0.0072
Carbon dioxide production (mL/h)
Day before sacrifice
M
Group effect: P=0.0006
Energy expenditure (kcal/h)
NS
Day before sacrifice
N
P=0.7933
Liver (% initial body weight)
O
P=0.2503
Plasma ASAT (U/L)
P
P=0.0409
Plasma ALAT (U/L)
Q
P=0.3080
Plasma LDH (U/L)
R
P<0.0001
Total CER levels in plasma (nmol/mL)
S
CER levels in plasma (FC of PBS-V)
CER(16:0) CER(20:0) CER(22:0) CER(22:1) CER(24:0) CER(24:1) CER(26:0)
T
P<0.0001
Total HCER levels in plasma (nmol/mL)
U
HCER levels in plasma (FC of PBS-V)
HCER(16:0) HCER(18:0) HCER(20:0) HCER(22:0) HCER(22:1) HCER(24:0) HCER(24:1)
V
P=0.0286
Total LCER levels in plasma (nmol/mL)
W
P=0.4972
eWAT (% initial body weight)
X
P=0.0007
Heart (% initial body weight)
Y
P=0.0716
Gastrocnemius (% initial body weight)
Z
P=0.8419
Initial grip strength (g)
PBS-V C26-V C26-M
AA
P=0.0117
P=0.119
Final grip strength (g)
PBS-V C26-V C26-M
AB
Heart
mRNA levels in heart (FC of PBS-V)
Foxo3 Fbxo32 Trim63 Gabarapl1 Map1lc3b Ctsl Bnip3
AC
Gastroc.
mRNA levels in gastroc. (FC of PBS-V)
Foxo3 Fbxo32 Trim63 Gabarapl1 Map1lc3b Ctsl Bnip3
C26-V
C26-M

Supplemental Figure 4. Pharmacological CER synthesis inhibition led to moderate improvements in body wasting in early cachexia. (A) Mice injected with C26 cells and treated with vehicle (C26-V) or myriocin (C26-M). Experiment ended once control animals lost > 8 % of their initial body weight (**early cachexia experiment**; n=16 animals per group, unless stated otherwise). Horizontal dotted lines show reference levels of healthy controls (PBS-V, PBS-injected vehicle-treated, n=10 animals). **(B** and **C)** Initial body weights at the start of experiment (injection of C26 cells, **B**), and myriocin treatment **(C)**. **(D)** Final body weight. **(E)** Body weight loss. **(F)** Final tumor weight. **(G-M)** Metabolic-cage monitoring (C26-V, n=7; C26-M, n=9; PBS-V, n=6 animals). ANOVA *P* value for the comparison of the 3 groups is displayed on the graphs but only significance from post-hoc tests between C26-V and C26-M is displayed on (I-M). **(G-H)** Cumulative food **(G)** and water **(H)** intakes. **(I)** Physical activity, **(J)** respiratory exchange ratio, **(K)** oxygen consumption, **(L)** carbon dioxide production and **(M)** energy expenditure at endpoint (last 24h before sacrifice, once control animals developed cachexia). **(N)** Liver weight. **(O-Q)** Markers of liver toxicity: plasma levels of ASAT **(O)**, ALAT **(P)**, and lactate dehydrogenase **(Q)** (C26-V, n=10; C26-M, n=9 animals). **(R-V)** Total CER **(R)**, HCER **(T)**, and LCER **(V)** levels in plasma, and plasma CER **(S)** and HCER **(U)** composition relative to PBS-V controls (n=4 animals per group). **(W)** Epididymal adipose tissue weight (2 depots). **(X)** Heart weight. **(Y)** Gastrocnemius muscle weight. **(Z** and **AA)** Initial **(Z)** and final **(AA)** grip strength. **(AB** and **AC)** Atrophy and autophagy marker mRNA levels in heart **(AB)** and gastrocnemius muscle (**AC**) (n=10 animals per group). Data are mean ± s.e.m. $*P < 0.05$, $** P < 0.01$, $*** P < 0.001$, $**** P < 0.0001$. Statistical analysis: unpaired two-tailed *t* test (D-F, N-O, Q, R-U, X-Y, AC), Mann and Whitney's test (B-C, G-H, P, U-W, AB-AC), unpaired one-way ANOVA with Dunett's post-hoc tests (Z-AA) or paired two-way ANOVA with Tukey's (I-J, L) or Sidak's (K, M) post-hoc tests.

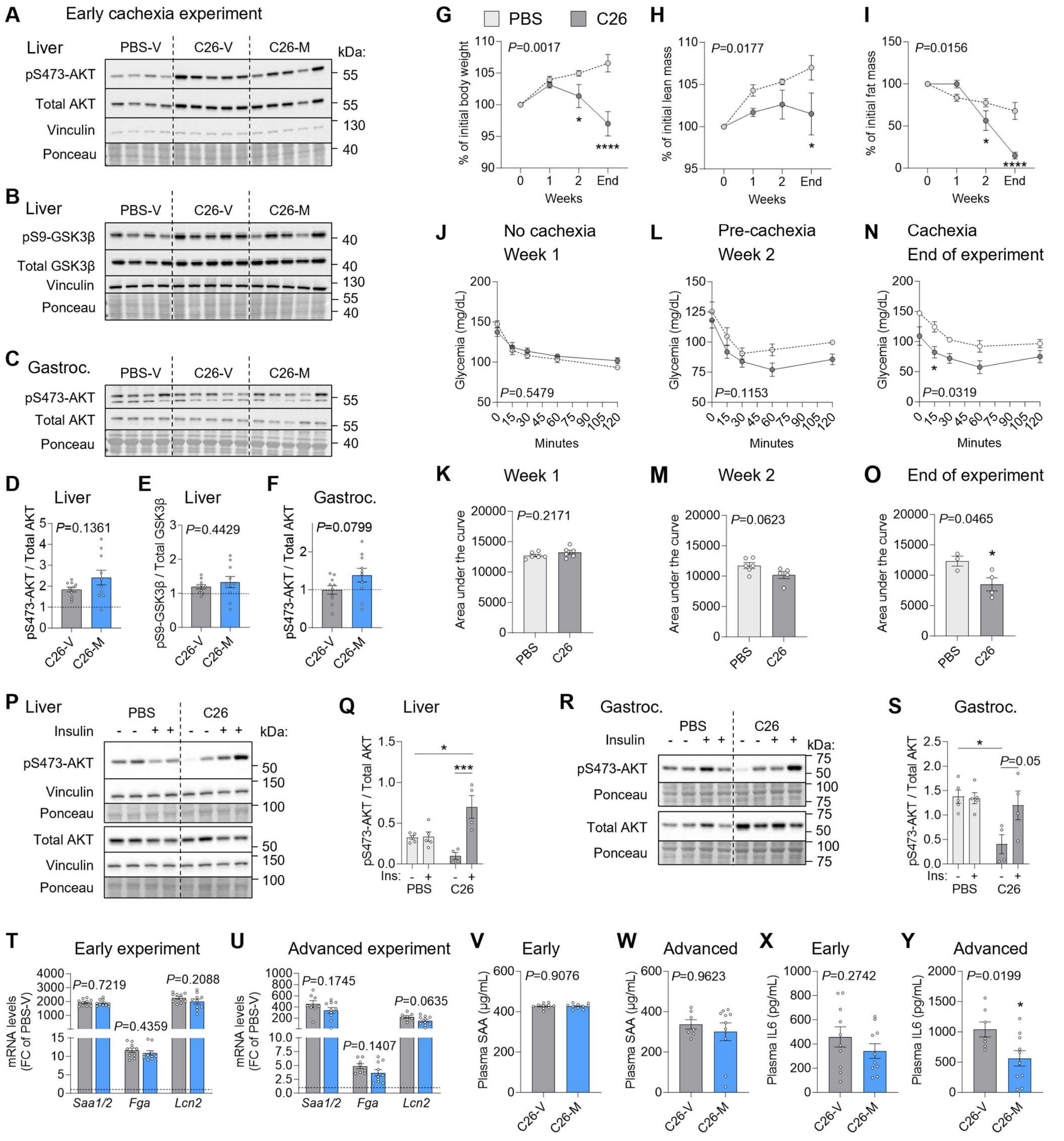

Supplemental Figure 5. CER levels are not associated with insulin resistance, acute phase response or IL6 levels in cachexia. **(A-F)** Western blot of insulin-signalling proteins (pS473-AKT, AKT, pS9-GSK3β, GSK3β) in liver (**A** and **D**, **B** and **E**) and gastrocnemius (**C** and **F**) of C26-tumor mice treated with vehicle (C26-V) or myriocin (C26-M) (early cachexia experiment, see **Supplemental Figure 4**). Vinculin and/or ponceau as loading controls. n=10 animals per group. **(A-C)** Representative blots and **(D-F)** quantifications (relative to PBS-V). Horizontal dotted

lines show levels of PBS-injected vehicle-treated controls. **(G-O)** Mice injected with PBS or C26 cells (n=6 animals per group). Animals were subjected to insulin tolerance tests at different stages: 1 week post-tumor implantation (No cachexia, n=6 per group, **J and K**); 2 weeks post-implantation (Pre-cachexia, PBS n=6, C26 n=5, **L and M**); and endpoint (Cachexia, 2.5-3 weeks post-implantation, PBS, n=3; C26, n=4; **N and O**). Glycemia reported in mg/dL **(J, L, N)** and respective area under the curve (AUC) (**K, M, O**). Cachexia progression was evaluated by loss of body weight **(G)**, lean mass **(H)** and fat mass **(I)**. **(P-S)** In another experiment, at endpoint, animals were fasted 4 hours and injected with saline or insulin 15min before tissue collection (PBS, n=5; C26, n=4 animals). Insulin-signalling was assessed by western blot (pS473-AKT, AKT) in liver **(P** and **Q)** and gastrocnemius **(R** and **S)**. **(P, R)** Representative blots and **(Q, S)** quantifications. Vinculin and/or ponceau as loading controls. **(T** and **U)** Hepatic mRNA levels of acute-phase response genes in C26-V and C26-M mice from the early **(T,** n=10 animals per group**)** and advanced **(U,** C26-V, n=8; C26-M, n=10 animals**)** cachexia experiments. **(V-Y)** Plasma SAA **(V** and **W)** and IL6 **(X** and **Y)** in C26-V and C26-M mice from the early **(V, X,** n=10 animals per group**)** and advanced **(W, Y,** C26-V, n=7; C26-M, n=10 animals) cachexia experiments. Data are mean ± s.e.m. *$P < 0.05$, *** $P < 0.001$, **** $P < 0.0001$. Statistical analysis: unpaired two-tailed *t* test (D-F, K, M, O, T-V, X-Y), Mann and Whitney's test (T, W), paired two-way ANOVA with Sidak's (G-J, L, N) or unpaired two-way ANOVA with Tukey's (Q, S) post-hoc tests.

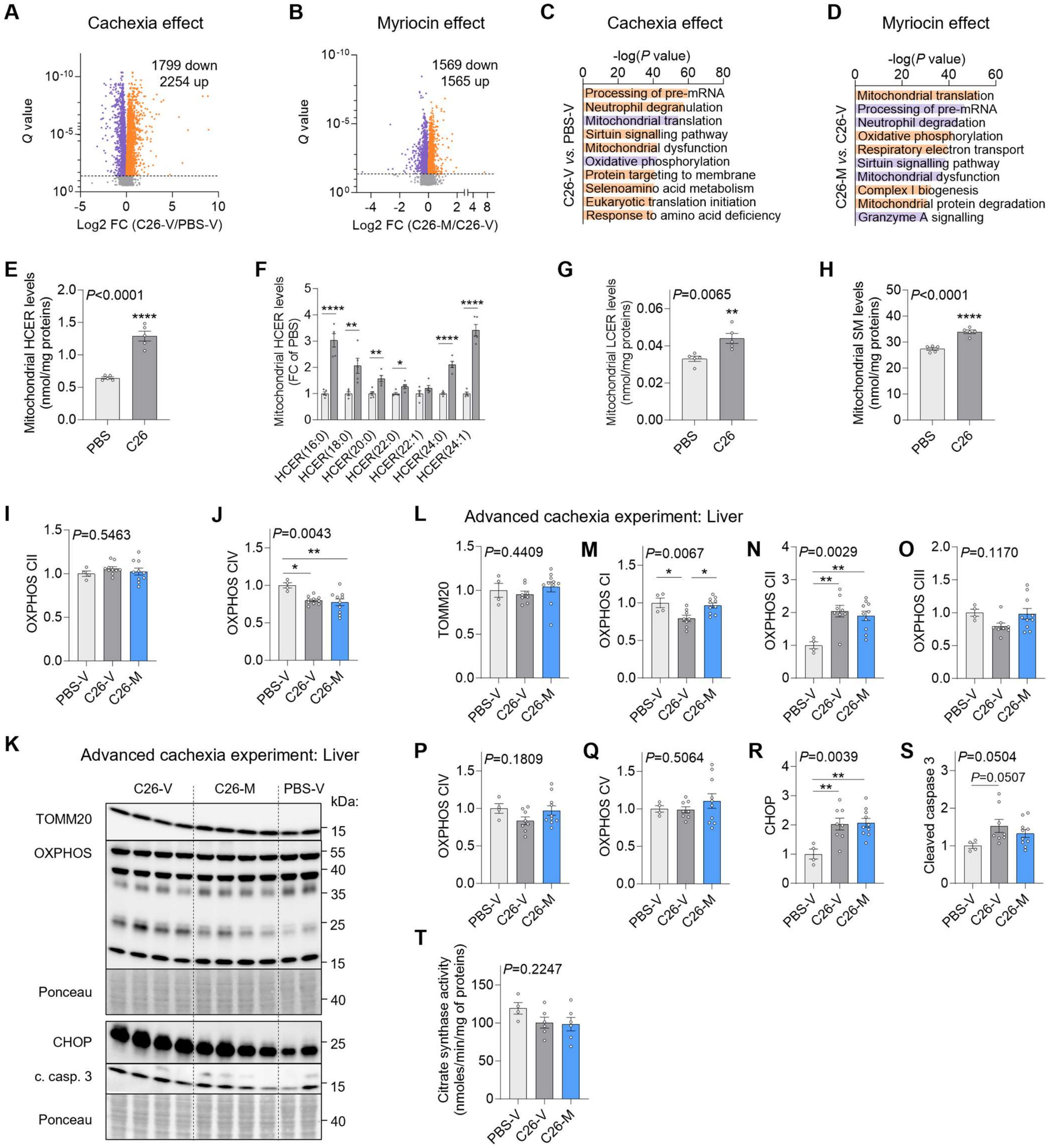

Supplemental Figure 6. CERs promote mitochondrial dysfunction in livers of cachectic animals. (A-D, I-J) Early cachexia experiment. See **Supplemental Figure 4**. C26-V: tumor mice, vehicle-treated; C26-M: tumor mice, myriocin-treated; PBS-V: tumor-free mice, vehicle treated. **(A-D) Early cachexia experiment:** liver proteomics (PBS-V, n=4; C26-V, -M, n=5 animals). Significantly altered proteins were defined by a *Q* value < 0.05 (horizontal dotted line). **(A** and **B)** Volcano plots of detected proteins and **(C** and **D)** Ingenuity Pathway Analysis (Qiagen)

showing the top pathways affected by cachexia (**A, C,** C26-V *vs.* PBS-V) and myriocin (**B, D,** C26-M *vs.* C26-V). Purple: pathway predicted as downregulated; Orange: upregulated. **(E-H)** Isolated crude mitochondria from livers of PBS-injected (healthy controls) and cachectic C26-tumor mice (n=5 animals per group). Total HCER **(E)**, LCER **(G)** and SM **(H)** levels, and **(F)** HCER composition relative to PBS controls. **(I** and **J)** Early cachexia experiment: OXPHOS complex II **(I)** and IV **(J)** protein levels in liver (fold change of PBS-V; PBS-V, n=4; C26-V, -M, n=10 animals). **(K-T) Advanced cachexia experiment. (K-S)** Western blots of mitochondria-related proteins in liver of PBS-V (n=4), C26-V (n=8) and C26-M (n=10) animals (See Figure 2): TOMM20 (**L**), OXPHOS (from top to bottom: CV, CIII, CIV, CII, CI; **M-Q**), CHOP (**R**) and cleaved caspase 3 (**S**). Ponceau as loading control. Quantifications relative to PBS-V. **(T)** Citrate synthase activity in liver. Data are mean ± s.e.m. *$P < 0.05$, ** $P < 0.01$, **** $P < 0.0001$. Statistical analysis: unpaired two-tailed *t* test (E-H), Mann and Whitney's test (F), unpaired one-way ANOVA with Tukey's post-hoc tests (I-J, M-R, T) or Kruskal-Wallis with Dunn's post-hoc tests (L, S).

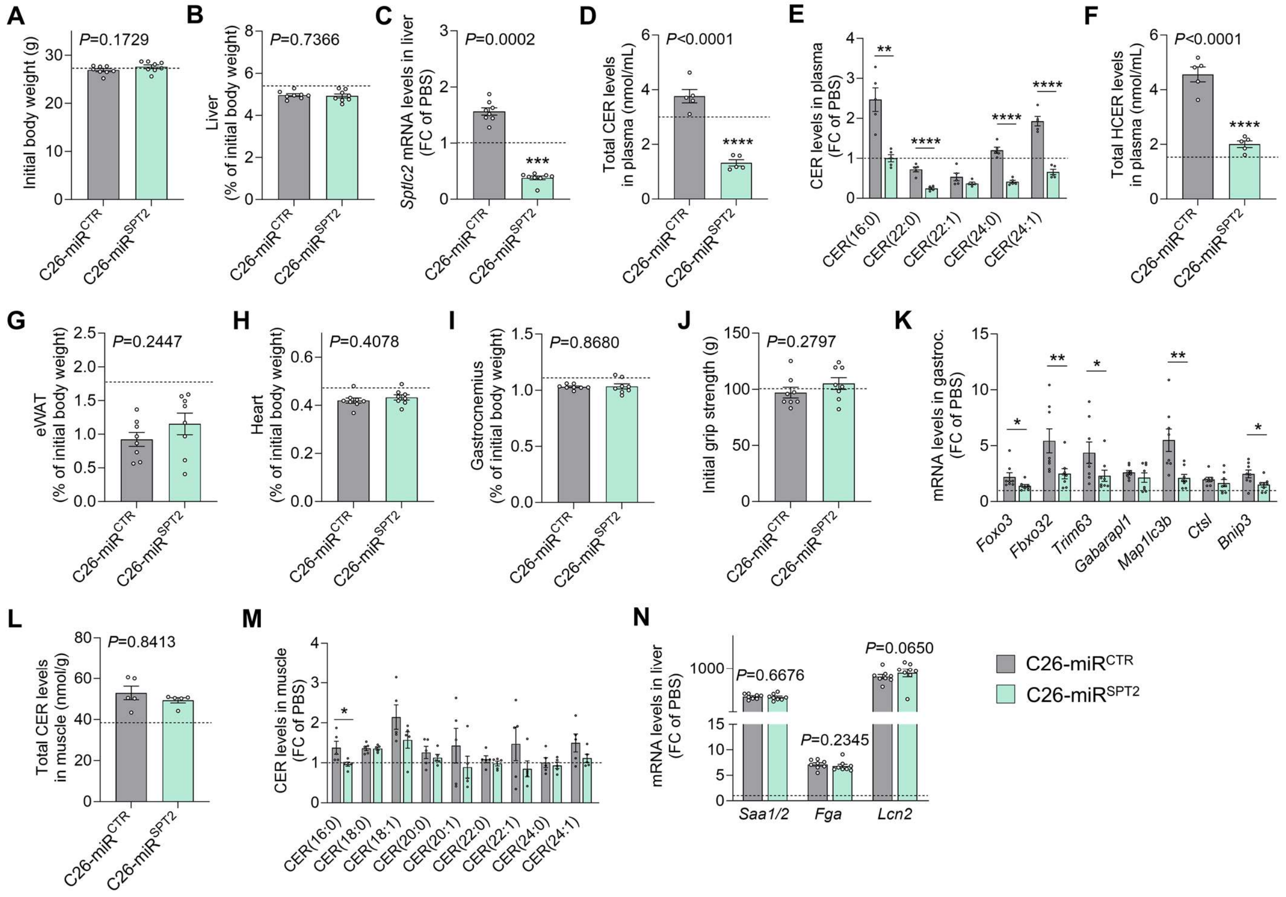

Supplemental Figure 7. Liver-specific invalidation of CER synthesis improves cachectic phenotypes and mitochondrial function in livers of cachectic animals. See **Figure 4**. Mice injected with hepatocyte-specific AAVs expressing either a control miRNA (miR^{CTR}) or a miRNA against *Sptlc2* (miR^{SPT2}), and with cachexia-inducing C26 carcinoma cells (n=8 animals per group, unless stated otherwise). Experiment was ended once control animals lost > 10 % of body weight (early cachexia experiment). Horizontal dotted lines show reference levels of healthy PBS-injected controls (n=4-7 animals). **(A)** Initial body weight. **(B)** Liver weight. **(C)** mRNA expression of *Sptlc2* in liver. **(D-F)** Total CER **(D)** and HCER **(F)** levels in plasma, and plasma CER **(E)** composition relative to PBS controls (n=5 animals per group). **(G-I)** Epididymal adipose tissue **(G)**, heart **(H)** and gastrocnemius muscle **(I)** weights. **(J)** Initial grip strength. **(K)** Atrogen and autophagy marker mRNA expressions in gastrocnemius muscle. **(L and M)** Total CER **(L)** and CER composition relative to PBS controls **(M)** in gastrocnemius muscle (n=5 animals per group). **(N)** mRNA expression levels of acute-phase response genes in liver. Data are mean ± s.e.m. *$P < 0.05$, ** $P < 0.01$, *** $P < 0.001$, **** $P < 0.0001$. Statistical analysis: unpaired two-tailed *t* test (A-B, D-J, K, M-N) or Mann and Whitney's test (C, K-N).

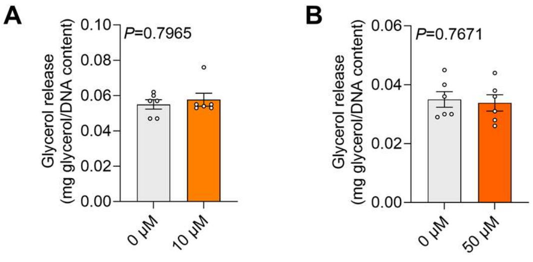

Supplemental Figure 8. CERs do not affect adipocyte wasting *in vitro.* **(A** and **B)** 3T3-L1 adipocytes were treated with different doses (**A,** 10μM; **B,** 50μM) of the CER analogue CER(6:0) for 24h. Measurement of glycerol released in cell culture media (n=6 replicates per group). Data are mean ± s.e.m. Statistical analysis: unpaired two-tailed *t* test (B) or Mann and Whitney's test (A).

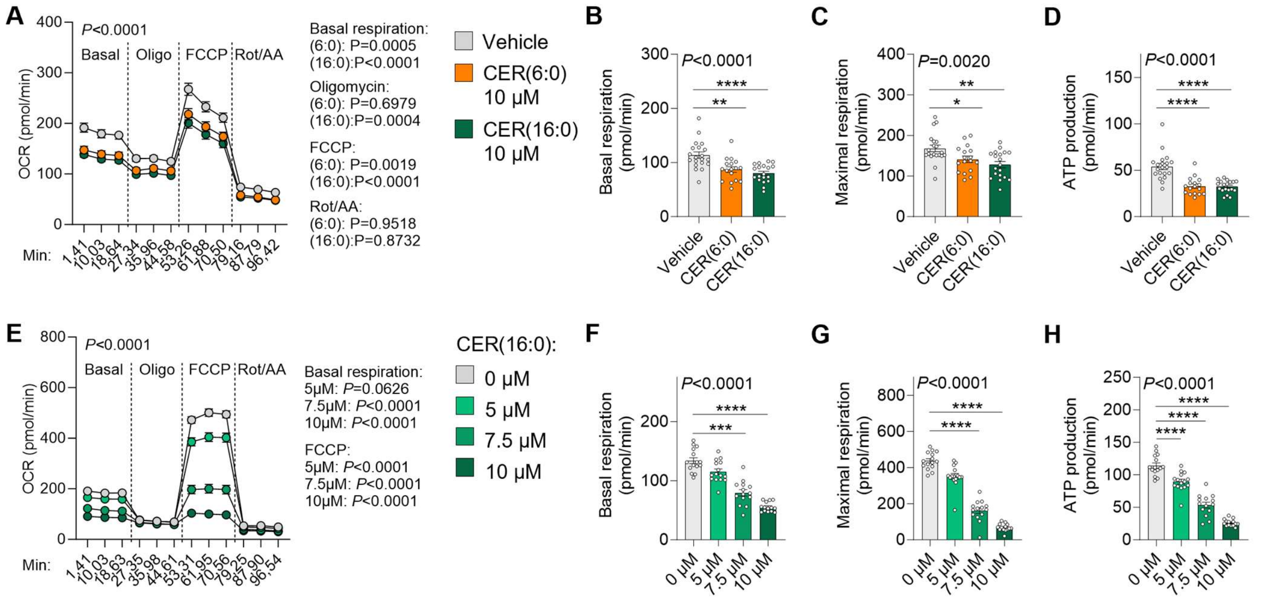

Supplemental Figure 9. CER(16:0) also leads to mitochondrial dysfunction in primary hepatocytes and C2C12 myotubes. (A-D) Primary hepatocytes and **(E-H)** C2C12 myotubes treated with the naturally occurring CER(16:0) or the CER analogue CER(6:0) for 16h. Mitochondrial respiratory measurements (normalized to protein content). **(A, E)** Oxygen consumption rate throughout the assay (hepatocytes: n=17-20 replicates; myotubes: n=14-15 replicates per group). **(B, F)** Basal and **(C, G)** maximal respiration, **(D, H)** ATP production. Data from vehicle (0 µM condition) and CER(6:0) in A-D are similar to the ones presented in Figure 5 G-J; data from the 0 µM condition in E-H are similar to the ones in Figure 5 M-P. Data are mean ± s.e.m. *$P < 0.05$, ** $P < 0.01$, *** $P < 0.001$, **** $P < 0.0001$ *vs.* 0µM condition. Statistical analysis: paired two-way ANOVA with Sidak's (A) or Dunnett's (E) post-hoc tests, unpaired one-way ANOVA with Dunnett's post-hoc tests (B-C, H), Kruskal Wallis with Dunn's post-hoc tests (D, F-G).

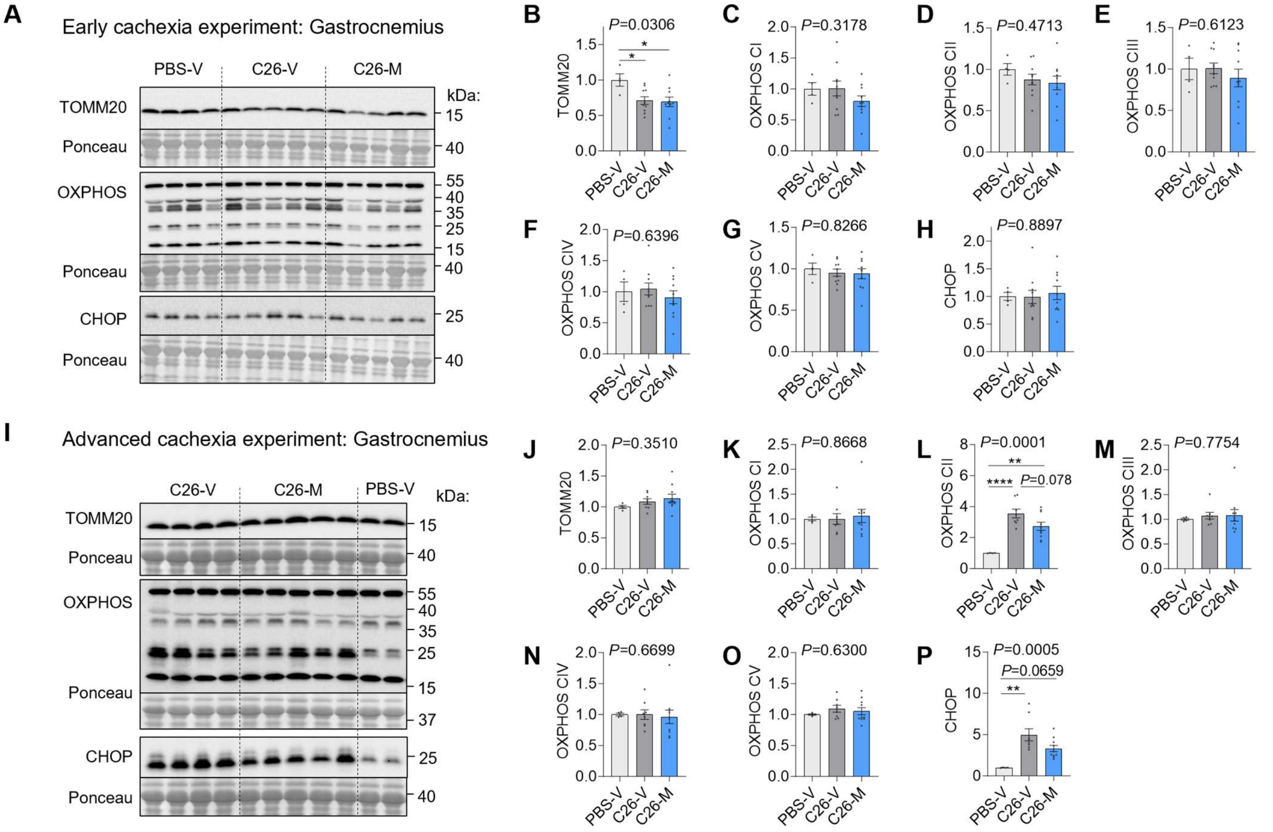

Supplemental Figure 10. Inhibition of CER synthesis improves mitochondrial phenotypes in skeletal muscle of cachectic animals. See Figure 6. Western blots of mitochondria-related proteins in gastrocnemius muscle of PBS mice treated with vehicle (PBS-V), C26-tumor mice treated with vehicle (C26-V), and C26-tumor mice treated with myriocin (C26-M): TOMM20, OXPHOS complexes (from top to bottom: CV, CIII, CIV, CII, CI), CHOP and cleaved caspase 3. Ponceau as loading control. **(A-H)** Mice from the **early cachexia experiment** (see **Supplemental Figure 4**; PBS-V, n=4; C26-V, -M, n=10 animals). **(I-P)** Mice from the **advanced cachexia experiment** (see **Figure 2**; PBS-V, n=4; C26-V, n=8; C26-M, n=10 animals). **(A, I)** Representative blots and **(B-H, J-P)** quantifications (relative to PBS-V). Ponceau in (A) for TOMM20 is the same as in Supplemental Figure 5C for AKT. The three Ponceau in (A) are from the same animals and sample preparations, but not from the same blots. Ponceau in (I) for OXPHOS is the same as in (I) for CHOP. Ponceau in (I) (TOMM20, CHOP) is from the same animals and sample preparations as in Figure 2V, but not from the same blots. Data are mean ± s.e.m. *$P < 0.05$, ** $P < 0.01$, **** $P < 0.0001$. Statistical analysis: unpaired one-way ANOVA with Tukey's post-hoc tests (B-H, J, L, O) or Kruskal-Wallis with Dunn's post-hoc tests (K, M-N, P).

	C26-V	C26-M	Statistical analysis	
iBW (g)	28.7 ± 0.6	27.3 ± 0.3	0.0552	Unpaired t test
			ANCOVA corrected for iBW	
Liver (mg)	1319.6 ± 53.6	1420.8 ± 29.3	0.7454	Interaction
			0.0579	Treatment effect
			0.3107	Effect of iBW
**eWAT (mg)**	51.7 ± 15.4	82.3 ± 21.9	0.7493	Interaction
			0.0061**	Treatment effect
			0.0048**	Effect of iBW
iWAT (mg)	46.0 ± 14.3	53.2 ± 14.6	0.6240	Interaction
			0.4047	Treatment effect
			0.3813	Effect of iBW
**Heart (mg)**	99.5 ± 2.2	102.7 ± 2.3	0.4296	Interaction
			0.0315*	Treatment effect
			0.0123*	Effect of iBW
Gastroc (mg)	205.1 ±7.8	223.7 ± 10.0	0.0882	Interaction
			0.0618	Treatment effect
			0.1418	Effect of iBW
**Soleus (mg)**	11.3 ± 0.7	12.4 ± 0.6	0.6783	Interaction
			0.0153*	Treatment effect
			0.0109*	Effect of iBW
**Tibialis (mg)**	58.3 ± 1.9	66.3 ± 2.4	0.6597	Interaction
			0.0011**	Treatment effect
			0.0113*	Effect of iBW
**EDL (mg)**	11.9 ± 0.9	13.2 ± 0.6	0.1992	Interaction
			0.0142*	Treatment effect
			0.0117*	Effect of iBW

Supplemental Table 1. Absolute values of tissue weights from C26-tumor-bearing mice treated with myriocin (advanced cachexia experiment). Mice injected with C26 cells and treated with vehicle (C26-V, n=7-8 animals) or myriocin (C26-M, blue, n=10 animals). Experiment ended once control animals lost > 20 % of their initial body weight (see Figure 2). As initial body weights (iBW) were almost significantly different between the C26-V and C26-M groups at treatment start (7 days post cancer cell injection), statistical analysis was performed using ANCOVA corrected for iBW. *P* values are displayed for interaction (homogeneity of the slopes), the effect of treatment (*i.e.* myriocin) and correction for iBW. * $P < 0.05$, ** $P < 0.01$.

	C26-V	C26-M	Statistical analysis	
iBW (g)	27.1 ± 0.5	27.4 ± 0.4	0.8743	Unpaired t test
			ANCOVA corrected for iBW	
Liver (g)	1.319 ± 0.025	1.329 ± 0.025	0.8444	Interaction
			0.8799	Treatment effect
			<0.0001****	Effect of iBW
eWAT (g)	0.271 ± 0.032	0.299 ± 0.034	0.5893	Interaction
			0.6882	Treatment effect
			0.2060	Effect of iBW
**Heart (g)**	0.109 ± 0.002	0.117 ± 0.002	0.4267	Interaction
			**0.0006****	Treatment effect
			<0.0001****	Effect of iBW
**Gastroc (g)**	0.269 ± 0.005	0.282 ± 0.003	0.5726	Interaction
			**0.0321***	Treatment effect
			<0.0001****	Effect of iBW

Supplemental Table 2. Absolute values of tissue weights from C26-tumor-bearing mice treated with myriocin (early cachexia experiment). Mice injected with C26 cells and treated with vehicle (C26-V) or myriocin (C26-M) (n=16 animals per group). Experiment ended once control animals lost > 8 % of their initial body weight (see Supplemental Figure 4). Statistical analysis was performed using ANCOVA corrected for iBW. *P* values are displayed for interaction (homogeneity of the slopes), the effect of treatment (*i.e.* myriocin) and correction for iBW. * $P < 0.05$, *** $P < 0.001$, **** $P < 0.0001$.

	C26-miR^{CTR}	C26-miR^{SPT2}	Statistical analysis	
iBW (g)	26.9 ± 0.3	27.6 ± 0.4	0.1729	Unpaired t test
			ANCOVA corrected for iBW	
Liver (g)	1.335 ± 0.026	1.359 ± 0.034	0.8137	Interaction
			0.5600	Treatment effect
			0.0028**	Effect of iBW
eWAT (g)	0.250 ± 0.030	0.317 ± 0.044	0.1575	Interaction
			0.5422	Treatment effect
			0.3641	Effect of iBW
Heart (g)	0.113 ± 0.002	0.119 ± 0.003	0.3901	Interaction
			0.0976	Treatment effect
			0.7971	Effect of iBW
Gastroc (g)	0.277 ± 0.004	0.285 ± 0.006	0.1799	Interaction
			0.6884	Treatment effect
			0.0634	Effect of iBW

Supplemental Table 3. Absolute values of tissue weights from C26-tumor-bearing mice with liver-specific *Sptlc2* silencing. Mice injected with hepatocyte-specific AAVs expressing either a control miRNA (miR^{CTR}) or a miRNA against *Sptlc2* (miR^{SPT2}), and with cachexia-inducing C26 carcinoma cells (n=8 animals per group). Experiment ended once control animals lost > 10 % of body weight (see Figure 4). Statistical analysis was performed

using ANCOVA corrected for iBW. *P* values are displayed for interaction (homogeneity of the slopes), the effect of treatment (*i.e.* miR^{SPT2}) and correction for iBW.

	No cachexia	Mild cachexia	Cachexia	Severe cachexia	ANOVA
	**BWL<2% or BWL>2% w/o sarcopenia**	**BWL>2% + sarcopenia**	**BWL>5%**	**BWL>10%**	***P* value**
**Patients (n)**	8	6	12	11	
Males (n)	5	4	4	8	
Females (n)	3	2	8	3	
**Age (years)**	65.1 ± 5.0	70.8 ± 6.6	68.3 ± 2.4	66.9 ± 2.5	*P=0.5123*
**Cancer type**					
Pancreatic ductal adenocarcinoma	5	5	8	6	
Colorectal adenocarcinoma	2	0	1	2	
Esophageal cancer	0	1	1	2	
Gastric cancer (n)	1	0	0	0	
Others (n)	0	0	2	1	
**Cancer stage (UICC)**					
1	0	1	3	1	
2	2	3	4	1	
3	2	0	4	4	
4	4	2	1	3	
**Body mass change**					
BMI 6 months before (kg/m²)	25.8 ± 0.8	23.9 ± 0.9	26.4 ± 1.0	26.3 ± 1.2	*P=0.4331*
Current BMI (kg/m²)	25.5 ± 0.8	23.1 ± 0.9	24.7 ± 0.9	22.6 ± 1.0	*P=0.1384*
BW 6 months before (kg)	76.1 ± 5.0	73.3 ± 4.1	73.9 ± 3.9	79.0 ± 5.8	*P=0.8425*
Current BW (kg)	75.2 ± 4.9	71.0 ± 4.0	69.1 ± 3.8	67.8 ± 5.0	*P=0.6992*
%BWL in past 6 months	1.2 ± 0.6	3.2 ± 0.2	6.6 ± 0.4**	14.0 ± 0.7****	*P<0.0001*
Sarcopenia (n)	3	6	6	6	

Supplemental Table 4. Clinical data of patients with gastrointestinal cancer. Patients were stratified based on their cachectic phenotype (Fearon's definition of cachexia (1)). No cachexia: patients with a weight loss up to 5% in the past 6 months without sarcopenia, or patients with sarcopenia and a weight loss < 2% (n=8 individuals); Mild cachexia: patients exhibiting a weight loss from 2 to 5 % with sarcopenia (n=6); Cachexia: weight loss from 5 to 10% with or without sarcopenia (n=12); Strong cachexia: patients with a weight loss > 10% with or without sarcopenia (n=11). UICC, Union Internationale Contre le Cancer. BMI: body mass index. BWL: body weight loss. Data are mean ± s.e.m. Statistical analysis: unpaired one-way ANOVA with Dunnett's post-hoc tests (BMI 6 months before, current BMI, BW 6 months before, current BW) or Kruskal Wallis with Dunn's post-hoc tests (age, % BWL in the past 6 months). ** $P < 0.01$, **** $P < 0.0001$ *vs.* No cachexia group.

Supplemental methods

Animal experiments

Generation of AAV vectors. *Sptlc2* miRNA sense and antisense sequences targeting gene accession number NM_011479.4 were designed using the Invitrogen BLOCK-iTTM RNAi Designer (F: 5'-TGCTGAATACCTGGTGTTCTCAGCCAGTTTTGGCCACTGACTGACTGGCTGAGCACCAGGTATT-3'; R: 5'-CCTGAATACCTGGTGCTCAGCCAGTCAGTCAGTGGCCAAAACTGGCTGAGAACACCAGGTATTC-3'), annealed and ligated into the linearized pcDNA™6.2-GW/± EmGFP-miR vector using T4 DNA ligase according to the manufacturer's instructions (Invitrogen). Ligated vectors were transformed into DH5 alpha bacteria (Life Technologies #ECO112) and positive transformants selected by resistance to spectinomycin. Plasmids were amplified and purified from overnight cultures by miniprep (Sigma-Aldrich #PLN70-1KT). Cloned miRNAs were sequenced and knock-down efficiency tested in HEK293T cells (ATCC #CRL-11268) transiently overexpressing a C-terminal flag-tagged SPTLC2 protein. To do so, cells (in 6-well plates) were transfected with 400 ng of *Sptlc2*-Flag overexpression plasmid and 5 to 200 ng of *Sptlc2*-miRNA plasmids using PEI (Polysciences #24765-1) according to manufacturer's instructions. Knockdown efficiency was compared against matching amounts of pcDNA™6.2-GW/± EmGFP miR-neg Control plasmid (Life Technologies). Cells were collected after 48 h and protein levels were determined by western blot using an anti-Flag antibody (Cell Signaling Technology #2368). *Sptlc2*-miR had a knock-down efficiency of 50 % (lowest miRNA dose) to 70% (highest dose). Control and *Sptlc2*-miRNA sequences were then subcloned into the AAV transfer vector pdsAAV2-LP1-GFPmut-miRNA using FastDigest restriction enzymes SalI and BglII (Thermo Scientific). These AAV vectors, which drive miRNA expression specifically in hepatocytes (2, 3) were transformed into SURE2 Supercompetent bacteria (Agilent #200152). Plasmids were purified from ampicillin-resistant clones with miniprep kits as described above. Clones carrying preserved viral ITR sequences (as confirmed by restriction digestion) were amplified using plasmid Mega prep kits (Qiagen, EndoFree Plasmid Mega kit #12381). Additional plasmids required for AAV production included the pDGΔVP helper plasmid (4) and the p5E18-VD2/8 expression vector (5) encoding AAV2 rep and a mutated AAV8 cap protein.

AAV production. AAV production was performed by seeding HEK293T cells into 10-stacks (Life Technologies #14000, #140400) for each virus condition. Cells were grown in high-glucose Dulbecco's modified Eagle's medium (DMEM) with pyruvate (Life Technologies #41966052), supplemented with 10% heat-inactivated fetal bovine serum

(FBS, Sigma-Aldrich #F7524) and 1% penicillin–streptomycin (Thermo Fisher Scientific #15140122). Once cells reached 70-80 % confluency, cells were transfected with AAV transfer, helper and rep/cap plasmids in a 1:1:1 molar ratio using PEI (7.5 mM) as transfection agent. Cells were harvested 48 h post transfection by using trypsin 0,25% EDTA (Invitrogen #25200-056), spun down at 2000 rpm for 10 minutes at 4 °C and resuspended in sterile 150 mM NaCl / Tris HCl pH 8.5. Cell suspensions were snap frozen and stored at -80 °C until virus purification.

AAV purification and titration. Lysis was completed by two cycles of thawing/snap-freezing. Cell debris were pelleted at 3,500 xg for 10 min at 4 °C and supernatants containing viruses were collected. The cell pellet was again resuspended in lysis buffer and underwent another round of lysis as previously described. Supernatants were again collected and pooled with the ones collected previously. Remaining cell pellets were further extracted in lysis buffer by incubating with 5mM MgCl2 and 50 U/mL benzonase (Sigma-Aldrich #E1014-25KU) at 37 °C for 30 min. Cell debris were once again centrifuged and supernatants were collected and pooled with the previous ones. Supernatant were then syringe filtered through 5 µm and 0.8 µm filters. Crude viral extracts were then layered on top of iodixanol density gradients (15-60% OptiPrep, Sigma-Aldrich). Ultracentrifuge tubes (Beckman Coulter #342414) were sealed, loaded into a 50.2Ti rotor and spun at 50,000 rpm for 2.5 h at 10 °C in an Optima XPN-80 Ultracentrifuge (Beckman Coulter). A 20G needle was inserted on top of the 60 % layer for ventilation and the 40 % layer containing AAVs were collected. AAVs were aliquoted and stored at -80 °C. For virus titration, 5 µL of virus was mixed with 5µL water and 10 µL 2M NaOH, and incubated at 56 °C for 30 min. After incubation, 10µL 2M HCl and 970 µL water were added (200 X dilution). 5 µL of this lysate was used for qPCR. Standard curves were obtained by serial dilution of the pdsAAV plasmid vector assuming that 63.9 ng/µL equals 10^{13} viral genomes/mL. qPCR using Taqman Gene Expression Master Mix (Life Technologies #4369510) was next performed with a primer/probe set targeting the EGFP sequence upstream of the miRNAs in the recombinant AAV genomes (forward primer 5’-GAGCGCACCATCTTCTTCAAG-3’, reverse primer 5’-TGTCGCCCTCGAACTTCAC-3’, probe 5’-FAM-ACGACGGCAACTACA-Tamra-3’). Virus concentrations were extrapolated from standard curves.

Primary hepatocyte isolation. Briefly, mice were anaesthetized (ketamine/xylazine), both abdominal walls were opened, and the liver was perfused through the vena cava with EGTA-containing HEPES/KH buffer for 6 min, followed by a collagenase-containing HEPES/KH buffer for 12–15 min until liver digestion was visible. The perfused liver was cut out and placed into a suspension buffer-containing dish and hepatocytes were gently washed out. After

filtering the cells through a 100-nm pore mesh, cells were centrifuged and washed twice and resuspended in suspension buffer. For a detailed isolation protocol including pictures, please see (6). The culture of primary hepatocytes is described in further details in the section *Cell culture*.

Metabolic cages

Food intake, water intake, physical activity, respiratory exchange ratio, oxygen consumption, carbon dioxide production and energy expenditure were measured in individually housed animals in PhenoMaster cages (TSE Systems, Germany) at 22 °C.

Assessment of insulin sensitivity

Insulin tolerance tests were performed one and two weeks post cancer cell injection, and once animals started to develop cachexia (body weight loss between 5 and 10%). Animals were fasted for 6 h before receiving a dose of 0.75 U/kg of insulin diluted in sterile saline solution intraperitoneally. Glycemia was recorded *via* a micro-puncture of the tail vein and a glucometer (Accu-Check #Performa). Body composition was assessed by EchoMRI™.

In another mouse cohort, as soon as C26 tumor-bearing mice had started to develop a clear cachectic phenotype (weight loss between 8 and 10%), animals were fasted for 4 h and injected with a single dose of saline solution or a bolus of 0.75 U/kg insulin. Animals were necropsied after exactly 15 min and tissues were immediately collected and snap-frozen to assess insulin signalling.

This experiment was approved by and performed in accordance with the guidelines of the local ethics committee from the UCLouvain, Belgium. Housing conditions were as specified by the Belgian Law of 29 May 2013, regarding the protection of laboratory animals.

Assessment of insulin sensitivity

Male CD2F1 mice (7 week-old, CRL Italy) were kept in specific pathogen-free conditions and housed in individually ventilated cages with a 12 h light/dark cycle and fed an irradiated chow diet (AO4-10, Safe, France). After one week acclimatization, either a saline solution or C26 cells (1×10^6 cells in 0.1 ml saline) were subcutaneously injected. Food intake and body weight were recorded. Eight mice were randomly assigned in each group based on their body weight on the day of cell injection. C26 + IL6-nAB group was treated with 300 µg monoclonal rat anti-murine IL-6

antibody (clone MP5-20F3, BioXCell, NH, USA); C26 + IgG group received 300 μg rat IgG1 isotype control (catalogue # BE0088, BioXCell, NH, USA). Treatments consisted of subcutaneous injections on days 7 and 9 after cancer cells administration, as previously described (7). Liver samples were kindly provided by Laure Bindels (Metabolism and Nutrition Research Group, Louvain Drug Research Institute, UCLouvain, Brussels, Belgium).

Cell culture

C26 cancer cells. C26 cells were kindly provided by Laure Bindels (Metabolism and Nutrition Research Group, Louvain Drug Research Institute, UCLouvain, Brussels, Belgium). Cells were grown in high-glucose DMEM with pyruvate (Life Technologies #41966052), supplemented with 10% heat-inactivated FBS (Sigma-Aldrich #F7524) and 1% penicillin–streptomycin (Thermo Fisher Scientific #15140122). Cells were seeded at a density of 13,500 cells/cm^2 and trypsinized (Thermo Fisher Scientific #25300054) every 2–3 days, once they reached 80% confluence.

Primary hepatocytes. Isolated primary mouse hepatocytes were seeded at a density of 200,000 cells per well in collagen-coated 24-well plates and 10,000 cells per well in collagen coated 96-well seahorse plates. The cells were seeded using William's Medium E supplemented with 10% FBS (PAN-Biotech), 5% penicillin-streptomycin, and 100 nM dexamethasone, and maintained at 37°C and 5% CO2. After 1 hour, the wells were washed with PBS and replenished with fresh William's Medium E containing 10% FBS and 5% penicillin-streptomycin. The medium was replenished next day and the experiments were performed within 48 hours after seeding.

C2C12 myotubes. C2C12 cells (ATCC #CRL-1772) were seeded at a density of 5,000 cells/cm^2 and grown in high-glucose Dulbecco's modified Eagle's medium (DMEM) with pyruvate and L-glutamine (Thermo Fisher Scientific #41966052) supplemented with 10 % heat-inactivated FBS (Sigma-Aldrich #F7524) and 1 % penicillin–streptomycin (Thermo Fisher Scientific #15140122) until they reached 80–100 % confluence. Media were then switched to 2 % FBS for 5 days to promote myotube differentiation. After 4-5 days, long contractile myotubes were used for experiments.

3T3-L1 adipocytes. 3T3-L1 cells (ATCC #CL-173) were seeded at a density of 6250 cells/cm2 and grown in high-glucose DMEM with pyruvate supplemented with 10% heat-inactivated FBS and 1% penicillin–streptomycin for 4 days until they reached 100% confluence. At Day 0, differentiation was initiated by adding 1 μg/mL insulin (Sigma-Aldrich #I2643), 0.25 μM dexamethasone (Sigma-Aldrich #D4902), 0.5 mM 3-isobutyl-1-methylxanthine (IBMX,

Sigma-Aldrich #I5879), 50µg/mL L-ascorbate (Sigma-Aldrich #A5960), 1µM Biotin (Sigma-Aldrich #B4639), and 17µM D-pantothenate (Sigma-Aldrich #P5155) to the media. Dexamethasone and IBMX were removed from the media after 4 days, insulin, ascorbate, biotin and pantothenate after 6 days of differentiation. The cells were differentiated for 3 extra days in normal media before use.

Adipocyte lipolysis

After 24 h of treatment, glycerol released into the medium by adipocytes was measured using a commercially available kit (Sigma-Aldrich #F6428). Data were normalized to initial glycerol content in the media and to DNA content of adipocytes present in the well (assessed *via* Hoechst staining) to account for cell number.

Real-time quantitative PCR analysis of mouse and human tissues

Pieces of mouse frozen tissues were homogenized in TRIzol (Life Technologies #15596018) using tissue lyser (Qiagen's TissueLyser II # 85300). RNA was then isolated by adding chloroform. After centrifugation, the upper phase containing RNA was mixed with a volume of 0.6 absolute ethanol. RNA samples were then loaded onto an Econospin column (Econospin # 1920-250), washed three times with RPE buffer (Qiagen #1018013), dried and eluted by adding RNA/DNAse-free water. RNA concentration was determined using Thermo Fisher Scientific #Nanodrop 2000. RNA was treated with DNase I (Thermo Fisher Scientific #18068-015) and reverse transcribed into cDNA (Life technologies #4368814) according to manufacturers' instructions. Real-time quantitative PCR of mouse samples was performed using Applied Biosystems QuantStudio 6 Flex Real-Time PCR System (Applied Biosystems, #4485691) and Takyon™ Low ROX Probe 2X MasterMix dTTP blue (Eurogentec #UF-LPMT-B0710) or Takyon™ Low ROX SYBR 2X MasterMix blue dTTP (Eurogentec #UF-LSMT-B0701). Mouse Taqman gene expression assays (Thermo Fisher Scientific #4331182): *Tbp* (Mm01277042_m1), *Hprt* (Mm03024075_m1), *Foxo3* (Mm01185722_m1), *Fbxo32* (Mm00499523_m1), *Trim63* (Mm01185221_m1), *Gabarapl1* (Mm00457880_m1), *Map1lc3b* (Mm00782868_sH), *Ctsl* (Mm00515597_m1), *Bnip3* (Mm01275600_g1), *Sptlc1* (Mm00447343_m1), *Sptlc2* (Mm00448871_m1), *Cers2* (Mm00504086_m1), *Cers5* (Mm00510998_m1), *Cers6* (Mm00556165_m1), *Asah1* (Mm00480021_m1), *Smpd1* (Mm00488318_m1), *Ugcg* (Mm00495925_m1), *St3gal5* (Mm00488237_m1), *Lcn2* (Mm_01324470_m1). Specific primers: *eGFP* (F: 5'-GAGCGCACCATCTTCTTCAAG-3'; R: 5'-TGTCGCCCTCGAACTTCAC-3'; Probe: 5'- ACGACGGCAACTACA-3'), *Saa1/2* (F: 5′-GGAGTCTGGGCTGCTGAGAAAA-3′; R: 5′-TGTCTGTTGGCTTCCTGGTCAG-3′), *Fga* (F: 5'-

GGATTCTAACTCACTGACCAGGA-3'; R: 5'-CCTCAGGATCTCAATTCTGCGC-3'). Gene expression was normalized to *Tbp* or *Hprt* mRNA levels. Gene expression $2^{-\Delta Ct}$ values were used for statistical analysis.

RNA from patients' tissue samples was isolated using TRIzol reagent and the MirVana™ miRNA Kit according manufacturer's instructions (Thermo Fisher Scientific). The amount and integrity of isolated RNA was assessed using a Bioanalyzer (Agilent Technologies, USA). RNA was reverse transcribed into cDNA (Life technologies #4368814) according to manufacturers' instructions. Real-time quantitative PCR of human samples was performed using LC480 Real-Time PCR system (Roche, Basel, Switzerland). mRNA levels were determined using Maxima SYBR Green Master Mix (Thermo Fisher Scientific #K0221). Human specific primers: *GAPDH* (F: 5'-GATCATCAGCAATGCCTCCTGC-3'; R: 5'-ACAGTCTTCTGGGTGGCAGTGA-3'), *SPTLC1* (F: 5'-GCAGTGTTGAAGGAAAAGTGCGG-3'; R: 5'-CAGTGCTCTCTTCCAGTTGTAGG-3'), *SPTLC2* (F: 5'-CCAGACTGTCAGGAGCAACCAT-3'; R: 5'-TTCGTGTCCGAGGCTGACCATA-3'), *KDSR* (F: 5'-CGGTTTCACAGCCTACTCTGCA-3'; R: 5'-GTTTTCTTCGGCAAAGCCAGGTG-3'), *CERS5* (F: 5'-GGTCACCATTGGGCTTATCTCC-3'; R: 5'-GTGTCACAGAGCCGCTGATACT-3'), *CERS6* (F: 5'-GACGCAATCAGGAGAAGCCAAG-3'; R: 5'-GGTAGTTGTACCAGCAATGCCTC-3'), *DEGS1* (F: 5'-CCAACATTCCTGGAAAAAGTCTTC-3'; R: 5'-GCCTCTTCATTCTTGAGTAGGGA -3'), *DEGS2* (F: 5'-GCCTCTCAACTGGATCACCTTC-3'; R: 5'-TGCGGCAGGTGGTCGTAGTAC-3'), *SMPD1* (F: 5'-GCTGGCTCTATGAAGCGATGGC-3'; R: 5'-AGAGCCAGAAGTTCTCACGGGA-3'), *ASAH1* (F: 5'-CTTTGCTGGCTATGTGGGCATG-3'; R: 5'-TGAGGAACCCTATCCACATGGC-3'), *UGCG* (F: 5'-CTTGGTTCACGGGCTGCCTTAC-3'; R: 5'-GAAACCAGTTACATTGGCAGAGAT-3'), *ST3GAL5* (F: 5'-AGAGCCTCAGTCAAGGTTCTGG-3'; R: 5'-GAGGTCATATCCAAAACCCGCC-3'). Gene expression was normalized to *GAPDH* mRNA levels. Gene expression $2^{-\Delta Ct}$ values were used for statistical analysis.

Western blot analysis

Proteins were extracted from frozen cells and tissues in RIPA (Sigma Aldrich # R0278) or ice-cold lysis buffer (50 mM Tris/HCl pH 7.2, 1 mM EDTA, 1 mM DTT, 0.15 M NaCl, 1% NP-40, 10 mM NaF, 2 mM Na3VO4) supplemented with 1 X cOmplete protease inhibitor cocktail and 1 X PhosSTOP cocktail (Sigma-Aldrich (Roche) # 11836170001, # 4906837001) using tissue lyser (Qiagen's TissueLyser II # 85300). Protein lysates were centrifuged at maximal speed for 20 min to pellet debris, and supernatants were used for western blot analysis. Protein extracts

were separated on 4–20% tris-glycine gels (Invitrogen #XP04205BOX), blotted onto nitrocellulose membranes (Cytiva #10600002) by using the Trans-Blot Turbo Transfer System (Bio-Rad). After Ponceau staining (Sigma-Aldrich #P7170) and blocking in 5% milk for 1 h, membranes were incubated overnight with the following primary antibodies: SPTLC2 (ABClonal, #A11716, polyclonal, 1/1000), ubiquitin (Cell Signaling Technology #3936, clone P4D1, 1/1,000), TOMM20 (Abcam #ab78547, polyclonal, 1/1,000), OXPHOS (Abcam #ab110413, polyclonal, 1/5,000), CHOP (Cell Signaling Technology #2895S, clone L63F7, 1/1,000), cleaved caspase 3 (Cell Signaling Technology #9664, clone 5A1E, 1/1,000), pS473-AKT (Cell Signaling Technology #4060, clone D9E, 1/1,000), total AKT (Cell Signaling Technology #9272, polyclonal, 1/1,000), pS9-GSKβ (Cell Signaling Technology #5558, clone D85E12, 1/1,000), total GSKβ (Cell Signaling Technology #9315, clone 27C10, 1/1,000), vinculin (Abcam # ab129002, clone EPR8185, 1/10,000). Anti-rabbit (Cell Signaling Technology #2729, polyclonal, 1/10,000) or anti-mouse IgG (Bio-rad #1706516, polyclonal, 1/10,000) coupled to horseradish peroxidase were used as secondary antibodies, and immunoreactive proteins were determined by chemiluminescence using Pierce ECL Western Blotting Substrate (Thermo Fisher Scientific #32209) or SuperSignalTM West Femto Maximum Sensitivity Substrate (Thermo Fisher Scientific #34095) and the ChemiDoc MP System (Bio-Rad). Blots were stripped between antibodies (Thermo Fisher Scientific #46430) in Supplemental Figures 5A-C. Protein expression was quantified by using the Image Lab software from Bio-Rad. Protein expression was normalized to vinculin or ponceau.

Isolation of crude mitochondria for lipidomics

300 mg pieces of fresh livers were collected from the big lobe and lysed in 1mL of ice-cold isolation buffer (250 mM mannitol, 75 mM sucrose, 100 µM K-EDTA, 10 mM KHEPES, 500 µM K-EGTA pH 7.4) using tissue lyser (Qiagen's TissueLyser II # 85300) set up at 30 Hz for 30 sec. Tissue lysates were centrifuged at 1,000 *xg* for 10 min at 4 °C to pellet unbroken cells and nuclei. Supernatants were transferred to new tubes and centrifuged again at 1,000 *xg* for 10 min at 4 °C twice. Cleared supernatants were transferred to new tubes and centrifuged at 9,000 *xg* for 10 min at 4 °C to pellet mitochondria. Supernatants containing plasma membranes, lysosomes, microsomes and cytosol were discarded, and pellets were gently washed in 1mL of ice-cold isolation buffer supplemented with 0.5 % fatty acid, protease-free BSA (Sigma-Aldrich #A7030) by gently pipetting up and down. Samples were again centrifuged at 10,000 *xg* for 10min at 4°C and the washing process was repeated two more times. Crude mitochondria pellets were then resuspended into a final volume of 150 µL ice-cold isolation buffer without BSA. Protein concentration

was determined using Pierce™ BCA protein assay kit (Thermo Fisher Scientific #23225). Samples were diluted into ice-cold isolation buffer at a fixed concentration of 3,3µg/µL. 80µL of mitochondrial suspension were used for lipidomics.

Determination of CER levels

Lipid analyses of the human cohort study as well as the mice fasting experiment (Figure 1 and 7) were performed using the Sciex Lipidyzer™ as described in (8). All further mice lipid analyses were carried out using the Differential Ion Mobility Shotgun Lipidomics Assay (DMS-SLA) (9). Sample homogenization, lipid extraction, lipidomics measurements, and lipidomics data processing were performed as extensively described in (10) and (11).

Briefly, frozen tissue samples (muscle, liver) were weighed into homogenization tubes with ceramic beads (1.4mm) and a cooled mixture (4 °C) of ethanol/phosphate buffer (85/15, v/v) was added to a final concentration of 0.33 mg/µL tissue. Tissue samples were subsequently homogenized using a Precellys 24 homogenizer (PEQLAB Biotechnology GmbH, Germany). 15 µL (equivalent to 5 mg) of the tissue homogenates or plasma, and 80 µL of crude mitochondrial fraction isolated from liver (3.3 mg/mL protein) were transferred into 1.5 mL glass vials together with 85 µL of MilliQ water (H2O). For accurate quantification, 25 µL of a mix of 77 deuterated internal standards were then added to the samples (Ultimate SplashOne, dFA 18:1, dFA 20:4, dCer d18:0/13:0, Glu Cer(d18:1-d7/15:0), dLacCer d18:1/15:0, 15:0-18:1-d7-PA, and Cardiolipin Mix I (Avanti Polar Lipids, Alabaster, AL, USA)).

Lipids were extracted 2x using a modified Matyash extraction protocol with methyl *tert*-buthyl ether (MTBE, LC grade) and methanol (MeOH, MS grade). The dried organic phases were reconstituted in 275 µL running solvent (10mM ammonium acetate in dichloromethane:MeOH (50:50, v/v)). For quality control purposes, pool samples of each tissue type were prepared in triplicates as described above.

For all samples analyzed using the DMS-SLA method, measurements were performed on a SCIEX Exion UHPLC system connected to a SCIEX QTRAP 6500+ mass spectrometer. The system was equipped with a SelexION differential ion mobility interface and operated using Analyst 1.6.3 software (SCIEX, Darmstadt, Germany). 75 µL of the re-dissolved sample were injected using the running solvent at an isocratic flow rate of 8 µL/min.

Sciex wiff files were converted to mzml using the Proteowizard msconvertGUI tool (v3.0.22074; https://proteowizard.sourceforge.io/download.html). The converted files were subsequently processed using the SLA

software (SLA.v1.5; https://github.com/syjgino/SLA/tree/v1.5-keyV4). Lipid species concentrations were corrected for Type-II isotopic overlap using lipid specific correction factors in ISOcorrectlistV4_1.31 and are reported in nmol/g for tissue, nmol/mL for plasma, and nmol/mg protein for mitochondria fraction.

Data were processed using R (version 4.4.1) and filtered according to a) missingness (<35% in pool samples, 50% per condition in biological samples), b) coefficient of variation (<25% in pool samples), and dispersion ratio (<50%) (12). Missing values were imputed using the GSimp imputation approach (13).

Proteomics

Lysis and digest. Frozen mouse livers were lysed in pre-cooled sodium deoxycholate (SDC) lysis buffer containing 4 % (w/v) SDC and 100 mM Tris -HCl (pH 8.5) and transferred into tubes filled with 1.4 mm ceramic beads (Soft tissue homogenizing CK14, – 0.5 mL; Bertin, Montigny-le-Bretonneux, FR) for tissue disruption. The samples were homogenized by shaking twice for 20 seconds at 5500 cycles per minute in a Precellys 24 homogenizer (Bertin, Montigny-le-Bretonneux, FR). Lysates were centrifuged for 10 minutes at 10 000 *xg* and supernatant transferred into a fresh tube. After tissue disruption, the mixture was boiled for 5 minutes at 95 °C. Following snap-freezing, samples were sonicated for 5 intervals with 1 second pulses, 5 seconds off and an intensity of 80 (Probe Sonicator EppiShear, ActiveMotif, Carlsbad, USA). Reduction and alkylation were performed at 45°C for 10 minutes using CAA and TCEP (Bond-Breaker 0.5M, Thermo Fisher Scientific). Over-night in-solution digestion was performed in a ThermoMixer at 2000 rpm at 37 °C with a protein to enzyme ratio for LysC and Trypsin of 1 to 100.

Sample cleanup by SDBRPS stage tipping. For all proteomic analyses, the digested peptides were acidified to a final concentration of 1% trifluoroacetic acid (TFA). The peptide solution was cleared by centrifugation and loaded onto activated (30% methanol, 1% TFA) double layer styrenedivinylbenzene–reversed phase sulfonated STAGE tips (SDB-RPS; 3 M Empore) (Kulak et al., 2014) with minor adjustments. The STAGE tips were first washed with 100µl 1% TFA in ethyl acetate, 100µl 1% TFA in isopropanol and 150µl 0.2% TFA. The peptides were eluted with 5% NH4OH in 80% acetonitrile. Samples were dried completely in a SpeedVac at 45°C and stored at -20°C until MS measurement.

Mass spectrometric measurement. The MS data were acquired in DIA mode on a Q Exactive HF mass spectrometer (Thermo Fisher Scientific). Equal amounts of peptides were automatically loaded to the online coupled RSLC (Ultimate 3000, Thermo Fisher Scientific) HPLC system. A Nano-Trap column was used (300-µm inner diameter

(ID) × 5 mm, packed with Acclaim PepMap100 C18, 5µm, 100 Å from LC Packings, Sunnyvale, CA, USA, before separation by reversed-phase chromatography (Acquity UPLC M-Class HSS T3 Column 75µm ID × 250 mm, 1.8µm from Waters, Eschborn, Germany) at 40°C. Peptides were eluted from the column at 250 nl/min using increasing ACN concentration in 0.1% formic acid from 3 to 40% over a 90-min gradient. The DIA method consisted of a survey scan from 300 to 1650 m/z at 120000 resolution and an automatic gain control (AGC) target of 3e6 or 100 ms maximum injection time. Fragmentation was performed via higher-energy collisional dissociation with a target value of 3e6 ions determined with predictive AGC. Precursor peptides were isolated with 37 variable windows spanning from 300 to 1650 m/z at 30000 resolution with an AGC target of 3e6 and automatic injection time. The normalized collision energy was 28, and the spectra were recorded in profile type.

Data analysis of MS spectra. DIA files were processed with Spectronaut (Version 19, Biognosys) as direct DIA analysis against a SwissProt mouse database (17081 sequences; release 2020_02 including spike proteins), using BSG factory settings for Pulsar with variable modifications set to acetylation of protein N-terminus, deamidation (NQ) and Oxidation (M). For DIA analysis, default settings were applied with the following changes: for quantification, precursor filtering was set on *Q* value and proteotypicity filter was set on protein group specific, LFQ method was set to automatic, quantity MS level was MS2, quantity type was area, cross run normalization was set automatic and major group quantity was calculated on protein group ID. Unpaired *t*-test was performed for differential abundance testing. Protein ratios are built from median peptide ratios per respective protein. Candidates filter was set to *Q* value confidence 0.05.

Ingenuity pathway analysis. Pathway analysis of liver proteomics was performed with the Ingenuity Pathway Analysis software (IPA, Qiagen). Tables containing protein names and Log2 fold change of protein expression with significant *Q* values (*Q* values < 0.05) were loaded into the software. Pathway analysis was based on Log2 fold change data.

Measurement of citrate synthase and OXPHOS complexes activities

Liver and muscle samples from PBS-V, C26-V, C26-M were snap-frozen in liquid nitrogen and homogenized in 10 mM of potassium phosphate buffer (pH = 7.4). The spectrophotometric activity of respiratory chain complexes I, II, III and IV, as well as citrate synthase, was measured as described (14). For complex I activity, 1–5 µl of tissue homogenate were pre-incubated for 10 min at 30 °C in a buffer containing 20 mM KH_2PO_4 buffer pH 8.0, 0.2 mM

NADH, 1 mM NaN_3, 1 mg/ml BSA (in EDTA 10 mM pH 7.4). Reactions were started by the addition of 50 µM CoQ_1 and NADH oxidation followed at 340 nm for 2 min. Rotenone was used to subtract any rotenone insensitive activity.

Complex II activity was measured by adding 1–5 µl of tissue homogenate to a mix containing 50 mM KH_2PO_4 buffer pH 7.0, 1.5 mM NaCN, 0.1 mM DCIP (2,6-dichlorophenolindophenol). Reactions were started by the addition of 50 µM CoQ_1 and the reduction of DCIPIP followed at 600 nm for 2 min.

Complex III activity was measured by adding 1–5 µl of tissue homogenate to a mix containing 50 mM KH_2PO_4 buffer pH 7.4, 2 mM NaN_3, 1 mg/ml BSA (in EDTA 10 mM pH 7.4), 50 µM cytochrome c, 50 µM reduced decylubiquinone. Cytochrome c reduction was measured at 30 °C and 600 nm for 2 min.

Complex IV activity was measured by adding 1–5 µl of tissue homogenate to a reaction mixture containing reduced cytochrome *c* (1.3 mg/mL) in 50 mM KH_2PO_4 buffer pH 7.0. The rate of oxidation of cytochrome c was recorded for 3 min (extinction coefficient of 27.2 mM^{-1} cm^{-1}) at 37 °C.

Citrate synthase activity was measured at 30 °C by using 1–5 µl of homogenate in a reaction mixture containing 75 mM Tris–HCl pH 8.0, 100 µM DTNB (5,5′-dithiobis(2-nitrobenzoic acid), 0.1 % Triton X-100 and 400 µM acetyl coenzyme A. The reaction was initiated by the addition of 500 µM oxaloacetate, and DTNB reduction at 412 nm measured for 2 min. The mitochondrial respiratory chain activities were expressed as nmoles/min/mg of protein.

Citrate synthase activity in livers of PBS, C26-miR^{CTR} and C26-miR^{SPT} was determined using a commercial assay (Abcam # ab239712), according to manufacturer's instructions. Data were normalized to protein content.

Serum analyzer

Mouse plasma levels of glucose, ASAT, ALAT, LDH were measured using a Beckman Coulter AU480 Chemistry Analyser.

Plasma glycerol and NEFA levels

Mouse plasma levels of glycerol and non-esterified fatty acids shown in Supplementary Figure 1 were measured using commercially available kits (Sigma-Aldrich #F6428, FUJIFILM Wako #994-91801, #990-91901) according to manufacturer's instructions.

SAA and IL6 ELISAs

Circulating levels of SAA and IL-6 were assessed using specific ELISA kits (R&D Systems #MSAA00, #M6000B) according to manufacturer's instructions. Plasma samples were diluted 1:15,000 (SAA) and 1/5 (IL6).

Supplemental references

1. Fearon K, Strasser F, Anker SD, Bosaeus I, Bruera E, Fainsinger RL, et al. Definition and classification of cancer cachexia: an international consensus. *Lancet Oncol.* 2011;12(5):489-95.
2. Nathwani AC, Gray JT, Ng CY, Zhou J, Spence Y, Waddington SN, et al. Self-complementary adeno-associated virus vectors containing a novel liver-specific human factor IX expression cassette enable highly efficient transduction of murine and nonhuman primate liver. *Blood.* 2006;107(7):2653-61.
3. Rose AJ, Berriel Diaz M, Reimann A, Klement J, Walcher T, Krones-Herzig A, et al. Molecular control of systemic bile acid homeostasis by the liver glucocorticoid receptor. *Cell Metab.* 2011;14(1):123-30.
4. Grimm D, Kern A, Rittner K, and Kleinschmidt JA. Novel tools for production and purification of recombinant adenoassociated virus vectors. *Hum Gene Ther.* 1998;9(18):2745-60.
5. Gao GP, Alvira MR, Wang L, Calcedo R, Johnston J, and Wilson JM. Novel adeno-associated viruses from rhesus monkeys as vectors for human gene therapy. *Proc Natl Acad Sci U S A.* 2002;99(18):11854-9.
6. Godoy P, Hewitt NJ, Albrecht U, Andersen ME, Ansari N, Bhattacharya S, et al. Recent advances in 2D and 3D in vitro systems using primary hepatocytes, alternative hepatocyte sources and non-parenchymal liver cells and their use in investigating mechanisms of hepatotoxicity, cell signaling and ADME. *Arch Toxicol.* 2013;87(8):1315-530.
7. Bindels LB, Neyrinck AM, Loumaye A, Catry E, Walgrave H, Cherbuy C, et al. Increased gut permeability in cancer cachexia: mechanisms and clinical relevance. *Oncotarget.* 2018;9(26):18224-38.
8. Morigny P, Zuber J, Haid M, Kaltenecker D, Riols F, Lima JDC, et al. High levels of modified ceramides are a defining feature of murine and human cancer cachexia. *J Cachexia Sarcopenia Muscle.* 2020;11(6):1459-75.
9. Su B, Bettcher LF, Hsieh WY, Hornburg D, Pearson MJ, Blomberg N, et al. A DMS Shotgun Lipidomics Workflow Application to Facilitate High-Throughput, Comprehensive Lipidomics. *J Am Soc Mass Spectrom.* 2021;32(11):2655-63.
10. El Bounkari O, Zan C, Yang B, Ebert S, Wagner J, Bugar E, et al. An atypical atherogenic chemokine that promotes advanced atherosclerosis and hepatic lipogenesis. *Nat Commun.* 2025;16(1):2297.
11. Riols F, Witting M, and Haid M. Differential Mobility Spectrometry-Based Cardiolipin Analysis. *Methods Mol Biol.* 2025;2855:373-85.
12. Broadhurst D, Goodacre R, Reinke SN, Kuligowski J, Wilson ID, Lewis MR, et al. Guidelines and considerations for the use of system suitability and quality control samples in mass spectrometry assays applied in untargeted clinical metabolomic studies. *Metabolomics.* 2018;14(6):72.
13. Wei R, Wang J, Jia E, Chen T, Ni Y, and Jia W. GSimp: A Gibbs sampler based left-censored missing value imputation approach for metabolomics studies. *PLoS Comput Biol.* 2018;14(1):e1005973.
14. Bugiani M, Invernizzi F, Alberio S, Briem E, Lamantea E, Carrara F, et al. Clinical and molecular findings in children with complex I deficiency. *Biochim Biophys Acta.* 2004;1659(2-3):136-47.
