# Supplementary material for: Inhibition of ceramide synthesis ameliorates body wasting in a cancer cachexia model
Source: J Clin Invest. 2026 May 15;136(10):e194687. doi: 10.1172/JCI194687 (PMC13178653; doi:10.1172/JCI194687)

Full unedited blots for:

Figure 2V: Ubiquitin

180 kDa
25 kDa

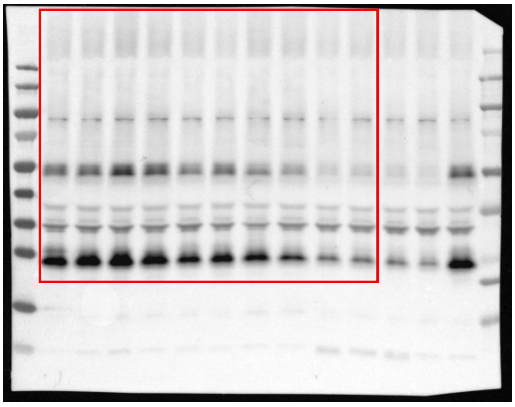

Figure 2V: Ponceau (Ubiquitin)

40 kDa
35 kDa

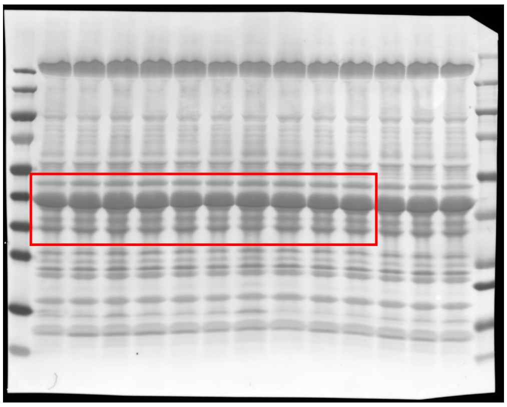

Figure 3N: TOMM20

15 kDa

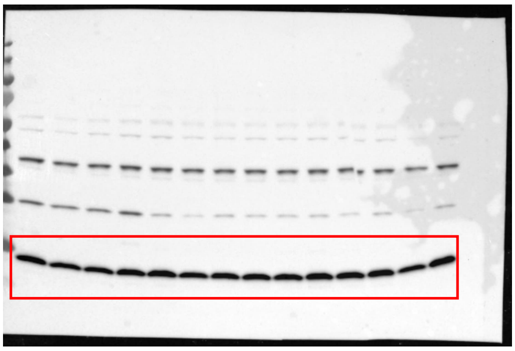

Figure 3N: Vinculin (TOMM20)

130 kDa
100 kDa

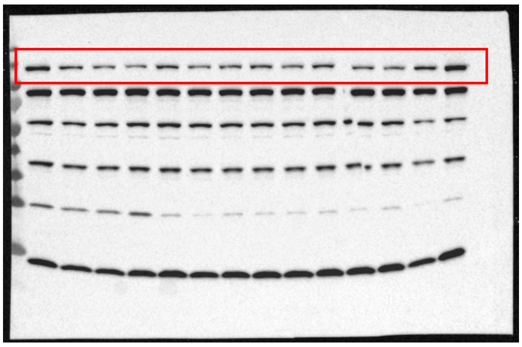

Figure 3N: OXPHOS

55 kDa
40 kDa
35 kDa
25 kDa
15 kDa

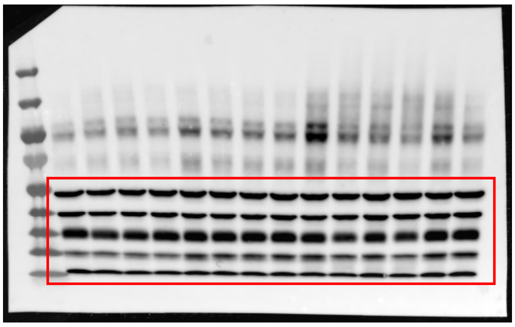

Figure 3N: Vinculin (OXPHOS)

130 kDa
100 kDa

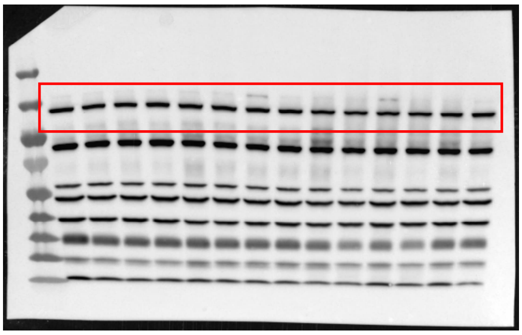

Figure 3N: CHOP

25 kDa
15 kDa

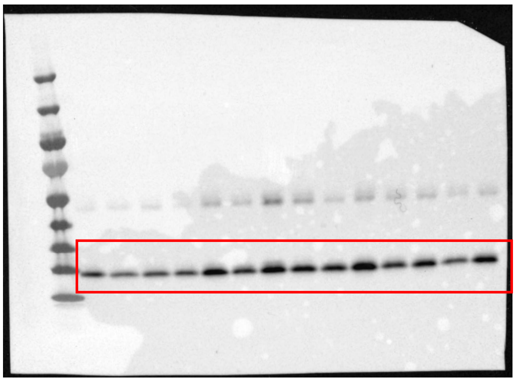

Figure 3N: Vinculin (CHOP)

130 kDa
100 kDa

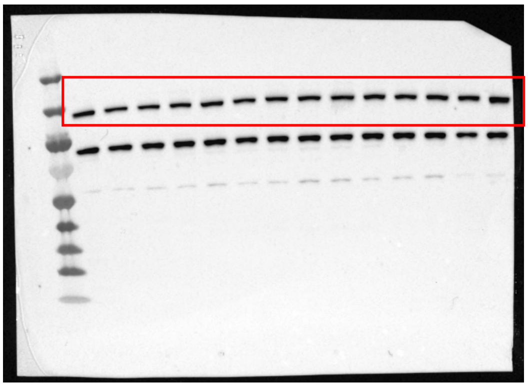

Full unedited blots for:

Figure 3N: Cleaved caspase 3

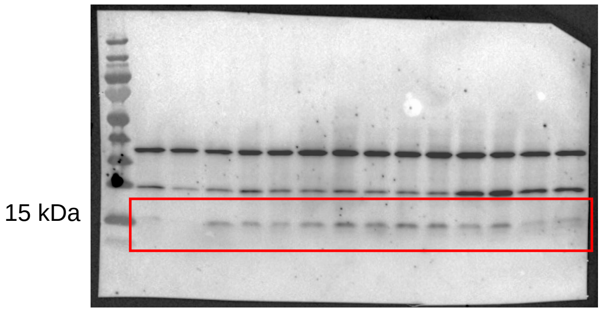

Figure 3N: Vinculin (c-casp. 3)

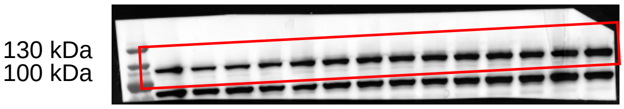

Figure 5E: OXPHOS

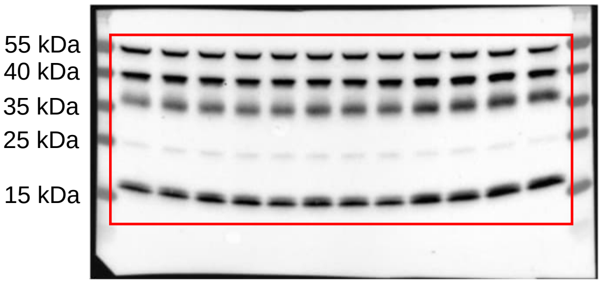

Figure 5E: Ponceau (OXPHOS)

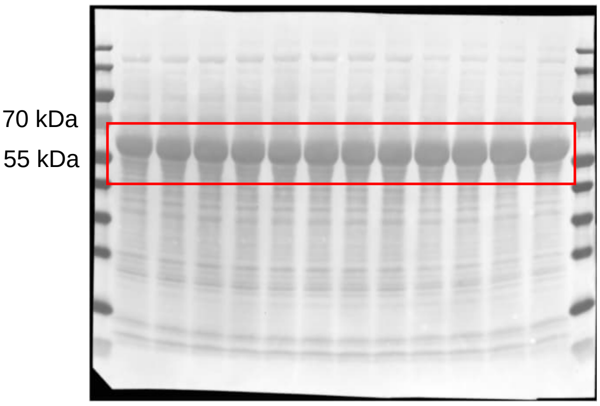

Figure 5E: Cleaved caspase 3

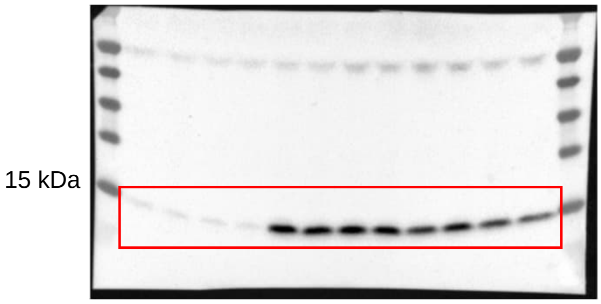

Figure 5E: Ponceau (c-casp. 3)

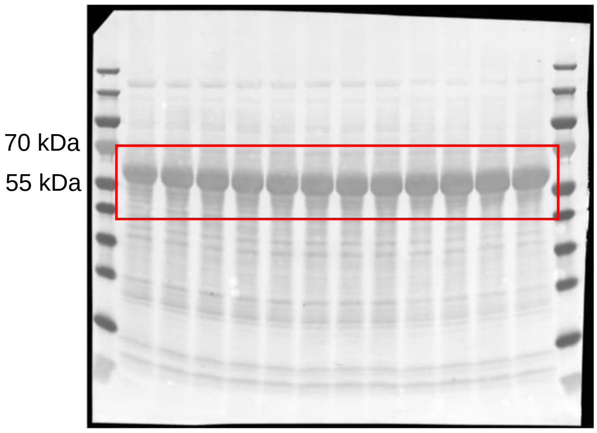

Figure 5K: OXPHOS

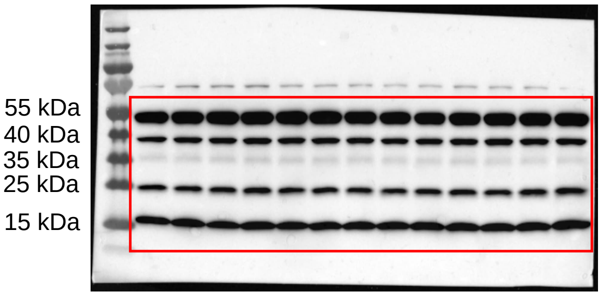

Figure 5K: Ponceau (OXPHOS)

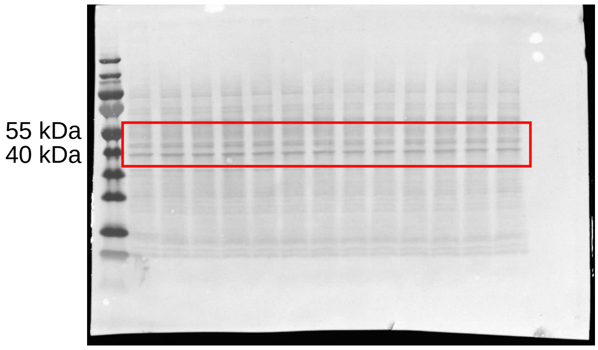

Full unedited blots for:

Figure 5K: Cleaved caspase 3

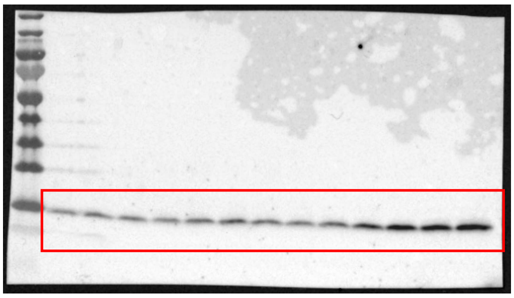

15 kDa

Figure 5K: Ponceau (c-casp. 3)

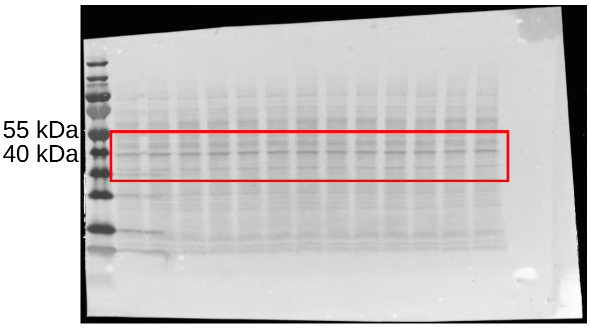

55 kDa
40 kDa

Supplemental Figure 1I: SPTLC2

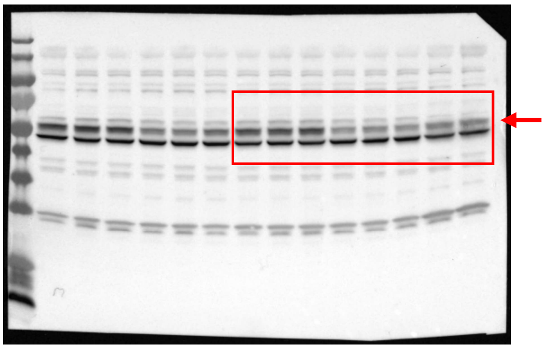

70 kDa
55 kDa

Supplemental Figure 1I: Vinculin

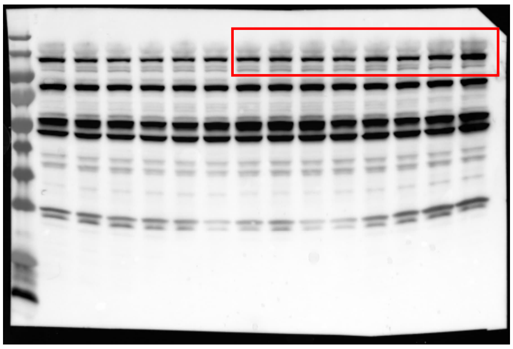

130 kDa
100 kDa

Supplemental Figure 5A: pS473-AKT

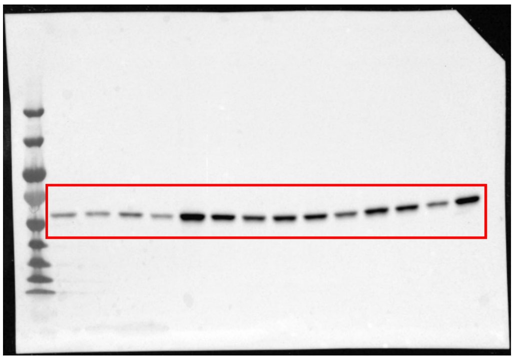

70 kDa
55 kDa

Supplemental Figure 5A: Total AKT

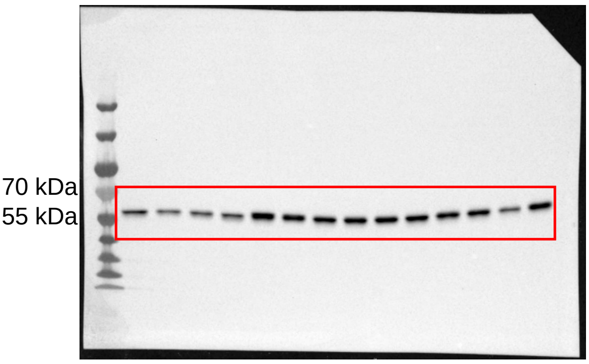

70 kDa
55 kDa

Supplemental Figure 5A: Vinculin

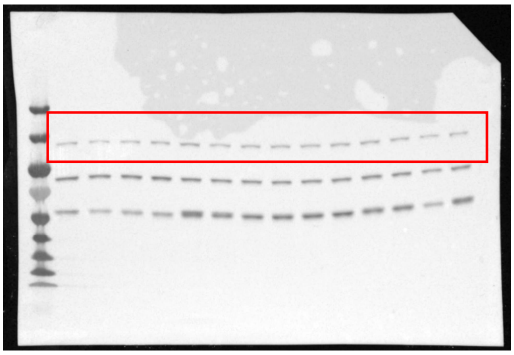

130 kDa
100 kDa

Supplemental Figure 5A: Ponceau

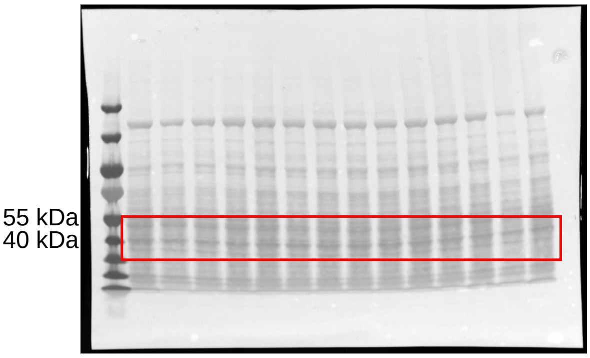

55 kDa
40 kDa

Full unedited blots for:

Supplemental Figure 5B: pS9-GSK3β

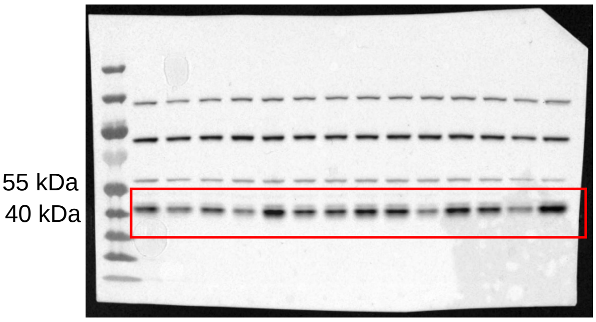

Supplemental Figure 5B: Total GSK

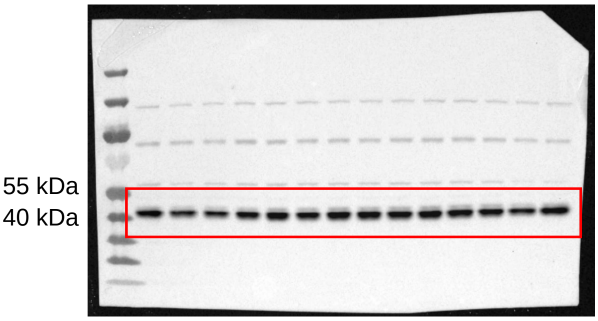

Supplemental Figure 5B: Vinculin

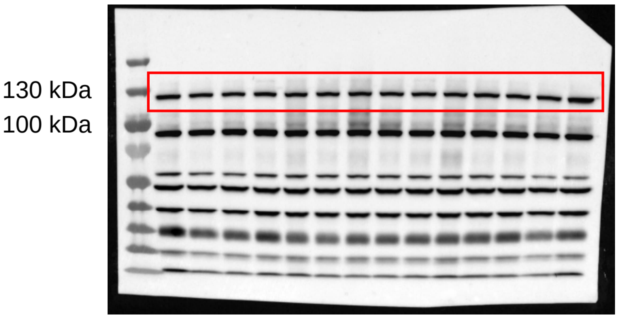

Supplemental Figure 5B: Ponceau

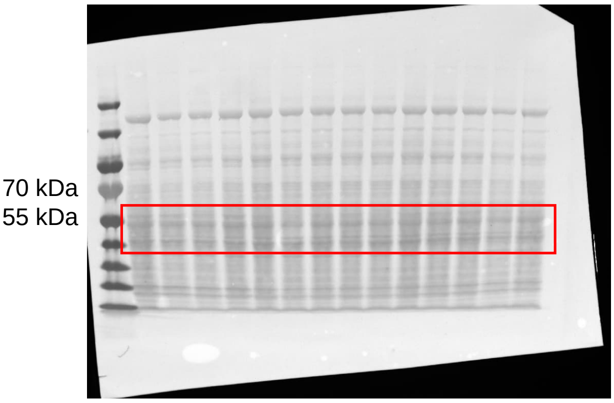

Supplemental Figure 5C: pS473-AKT

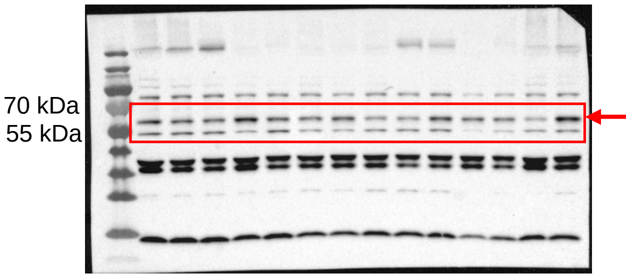

Supplemental Figure 5C: Total AKT

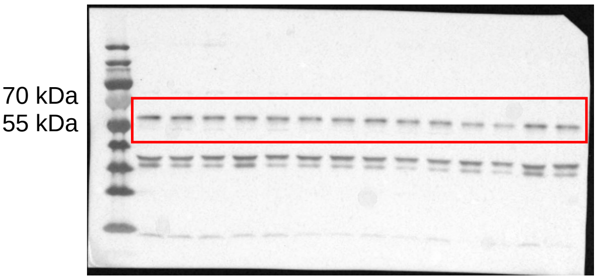

Supplemental Figure 5C: Ponceau

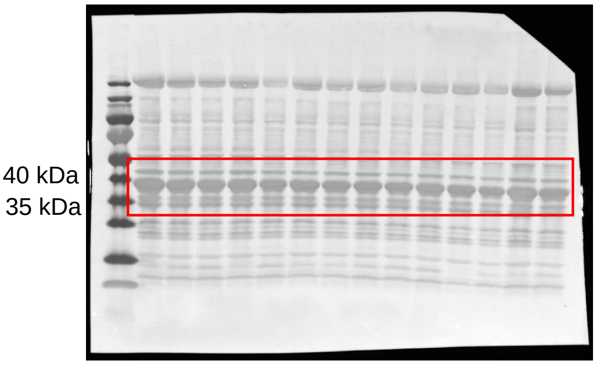

Full unedited blots for:

Supplemental Figure 5P: pS473-AKT

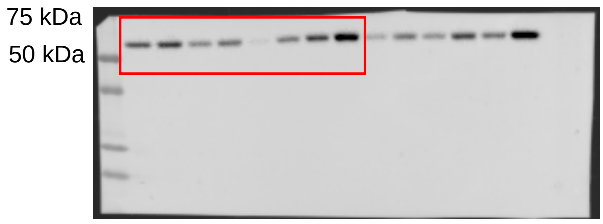

Supplemental Figure 5P:
Vinculin (pS473-AKT)

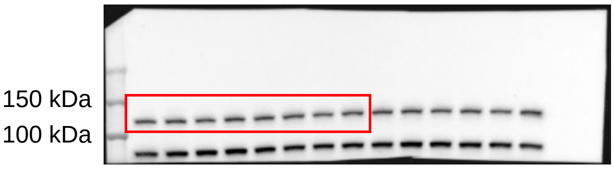

Supplemental Figure 5P:
Ponceau (pS473-AKT)

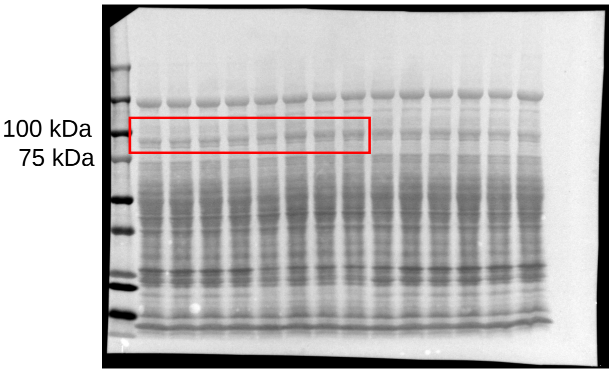

Supplemental Figure 5P: Total AKT

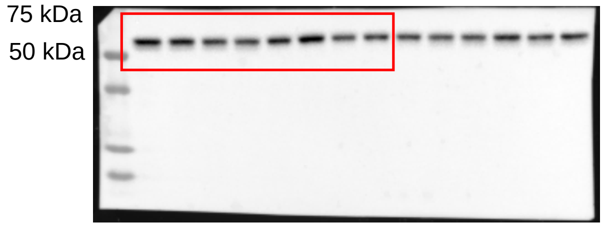

Supplemental Figure 5P:
Vinculin (Total AKT)

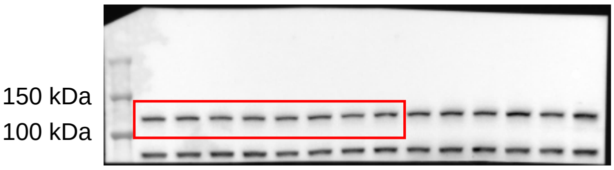

Supplemental Figure 5P:
Ponceau (Total AKT)

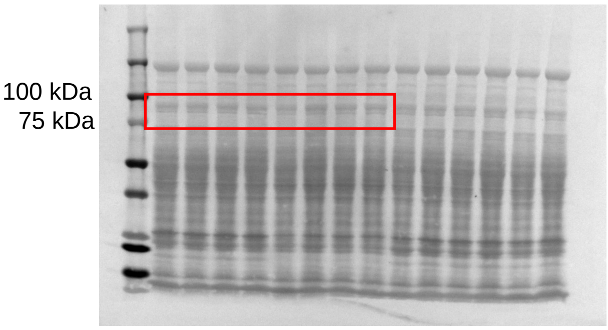

Full unedited blots for:

Supplemental Figure 5R:
pS473-AKT

75 kDa
50 kDa

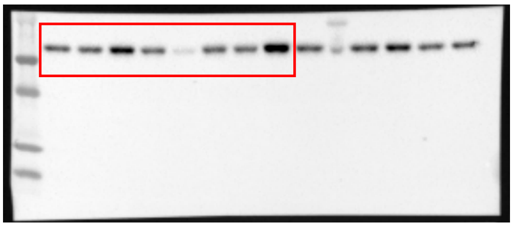

Supplemental Figure 5R:
Ponceau (pS473-AKT)

100 kDa
75 kDa

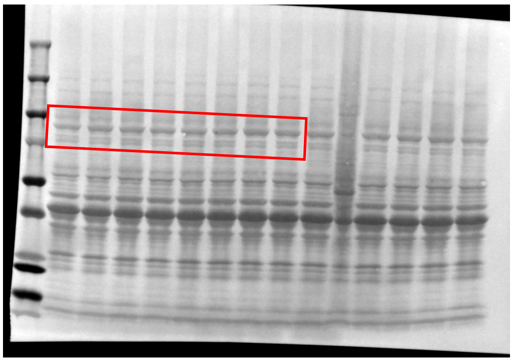

Supplemental Figure 5R:
Total AKT

75 kDa
50 kDa

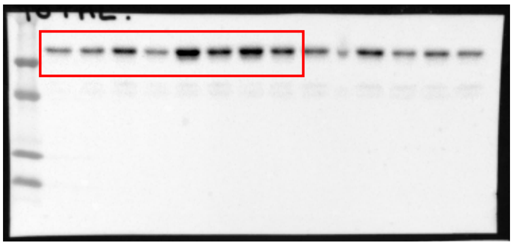

Supplemental Figure 5R:
Ponceau (Total AKT)

100 kDa
75 kDa

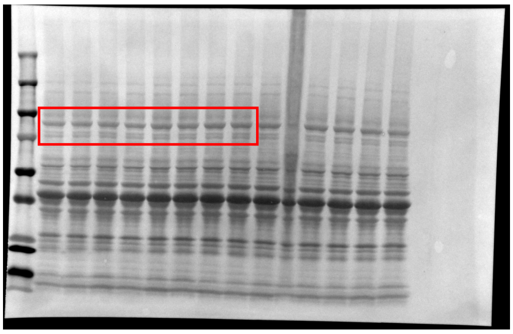

Supplemental Figure 6K: TOMM20

15 kDa

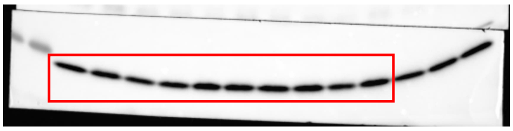

Supplemental Figure 6K: OXPHOS

55 kDa
40 kDa
35 kDa
25 kDa
15 kDa

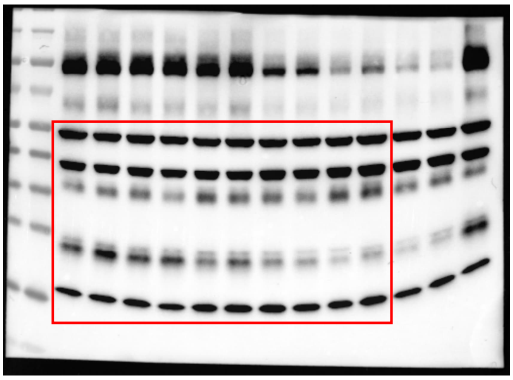

Supplemental Figure 6K:
Ponceau (TOMM20, OXPHOS)

70 kDa
55 kDa
40 kDa

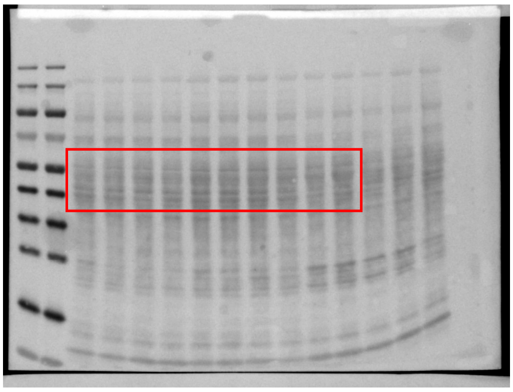

Full unedited blots for:

Supplemental Figure 6K:
CHOP

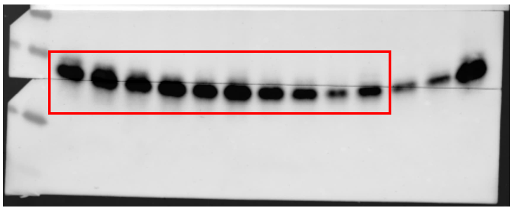

Supplemental Figure 6K:
Cleaved caspase 3

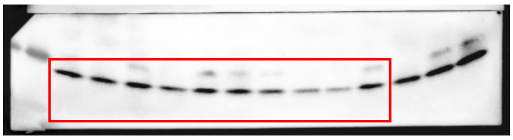

Supplemental Figure 6K:
Ponceau (CHOP, cleaved caspase 3)

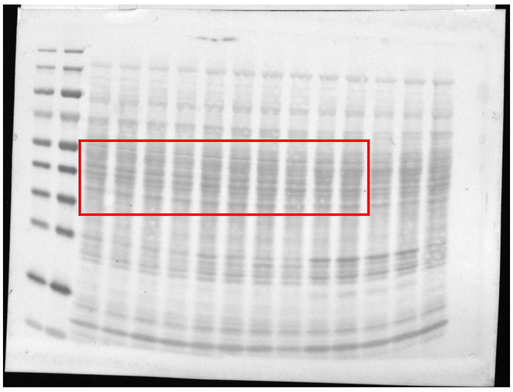

Supplemental Figure 10A:
TOMM20

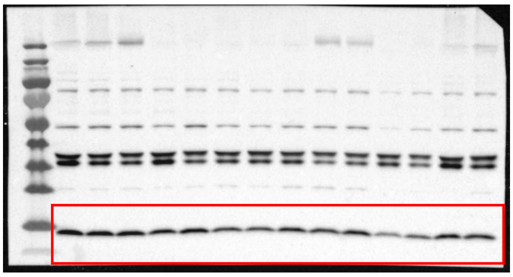

Supplemental Figure 10A:
Ponceau (TOMM20)

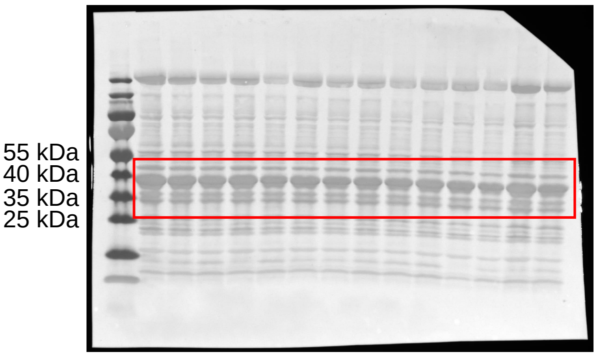

Supplemental Figure 10A:
OXPHOS

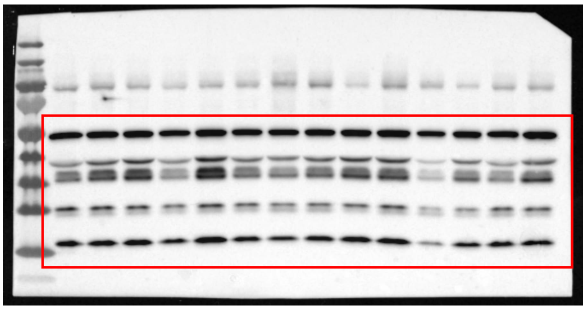

Supplemental Figure 10A:
Ponceau (OXPHOS)

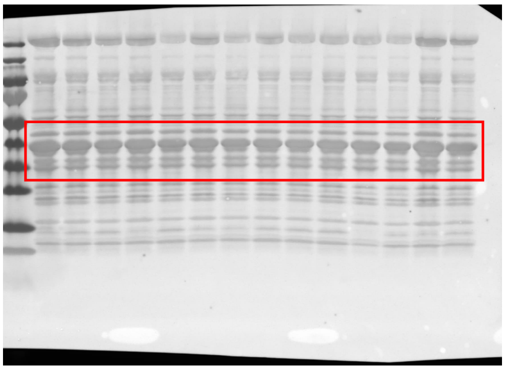

Full unedited blots for:

Supplemental Figure 10A:
CHOP

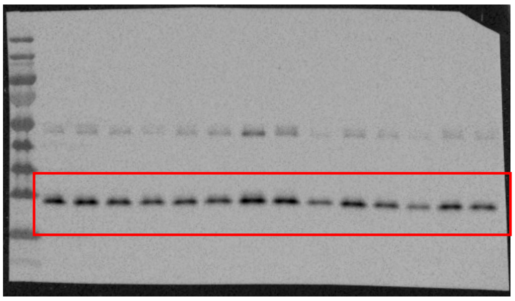

Supplemental Figure 10A:
Ponceau (CHOP)

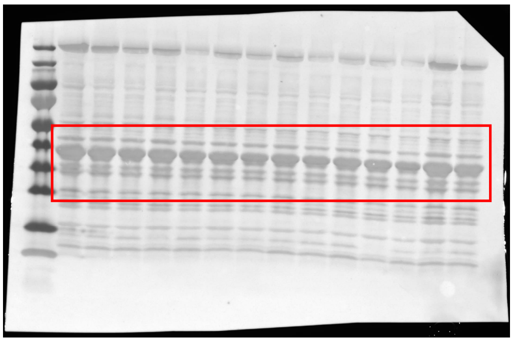

Supplemental Figure 10I:
TOMM20

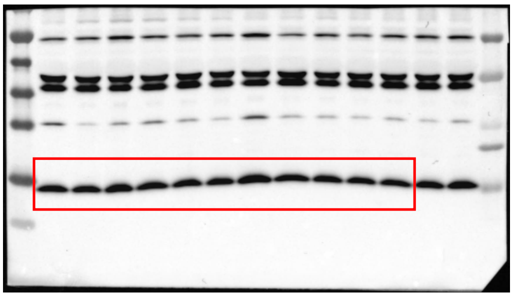

Supplemental Figure 10I:
Ponceau (TOMM20, CHOP)

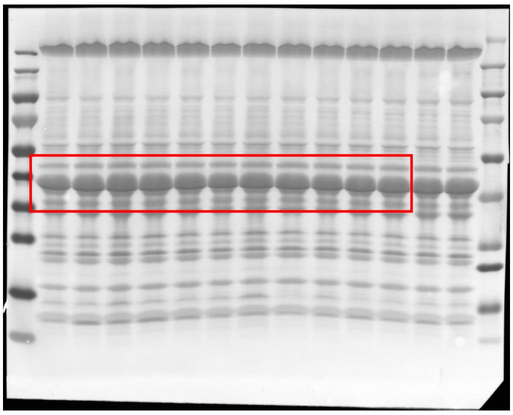

Supplemental Figure 10I:
CHOP

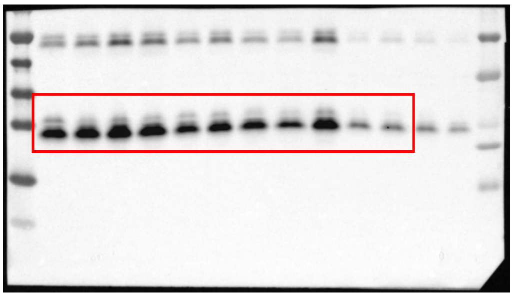

Supplemental Figure 10I:
OXPHOS

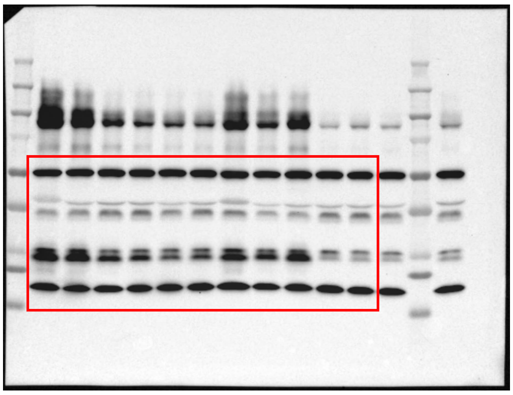

Supplemental Figure 10I:
Ponceau (OXPHOS)

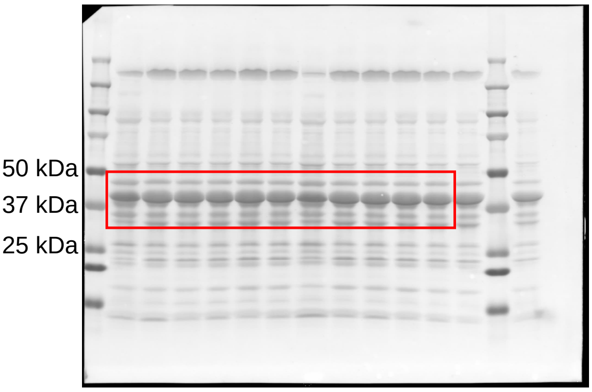

Supplement: Unedited blot and gel images [file jci-136-194687-s038.pdf]
